# Transition Metal Catalysis Controlled by Hydrogen Bonding in the Second Coordination Sphere

**DOI:** 10.1021/acs.chemrev.1c00862

**Published:** 2022-05-20

**Authors:** Joost N. H. Reek, Bas de Bruin, Sonja Pullen, Tiddo J. Mooibroek, Alexander M. Kluwer, Xavier Caumes

**Affiliations:** † Homogeneous and Supramolecular Catalysis, Van’t Hoff Institute for Molecular Sciences, University of Amsterdam, Science Park 904, 1098 XH Amsterdam, The Netherlands; ‡ InCatT B.V., Science Park 904, 1098 XH Amsterdam, The Netherlands

## Abstract

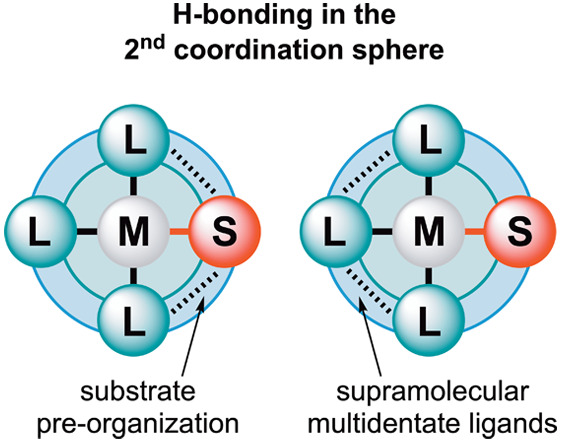

Transition metal catalysis is of utmost importance for the development of sustainable processes in academia and industry. The activity and selectivity of metal complexes are typically the result of the interplay between ligand and metal properties. As the ligand can be chemically altered, a large research focus has been on ligand development. More recently, it has been recognized that further control over activity and selectivity can be achieved by using the “second coordination sphere”, which can be seen as the region beyond the direct coordination sphere of the metal center. Hydrogen bonds appear to be very useful interactions in this context as they typically have sufficient strength and directionality to exert control of the second coordination sphere, yet hydrogen bonds are typically very dynamic, allowing fast turnover. In this review we have highlighted several key features of hydrogen bonding interactions and have summarized the use of hydrogen bonding to program the second coordination sphere. Such control can be achieved by bridging two ligands that are coordinated to a metal center to effectively lead to supramolecular bidentate ligands. In addition, hydrogen bonding can be used to preorganize a substrate that is coordinated to the metal center. Both strategies lead to catalysts with superior properties in a variety of metal catalyzed transformations, including (asymmetric) hydrogenation, hydroformylation, C–H activation, oxidation, radical-type transformations, and photochemical reactions.

## 1 Introduction

Homogeneous catalysis using metal complexes provides tools for effective and selective chemical transformations, which are crucial for the chemical process industry from both an economic and sustainability point of view. The field of homogeneous catalysis has been developed to an impressive level in the past 50 years, underscored by three awards for the Nobel prize in 2001,^1^ 2005,^2^ and 2010^3^ as well as the many applications of homogeneous catalysts found in industry.^4−6^ As the demands for the chemical industry are continuously changing, by the pressure to make the chemical industry more sustainable and based on renewable feedstocks as well as stimulated by product innovations in society, the demand for new catalysts is very high. As such, the research field of homogeneous catalysis is very active and many new catalytic conversions and novel concepts have been reported in recent years.

The properties of metal complexes that are used as catalysts are controlled, to a large extend, by the ligands that are coordinated to them, and therefore, traditionally a strong focus has been on ligand development. To aid the rational development of metal catalysts, many ligand parameters have been developed over the years.^7^ Rational development of catalysts started^7^ with primitive models to describe the electronic properties (Tolman’s χ-parameter)^8^ and size of the ligand (Tolman’s cone angle)^8^ and, somewhat later, the ligand bite angle.^9^ Nowadays, very sophisticated models can be used with the available computational tools.^10^ Currently, new machine learning strategies are also being developed to further aid the development of novel catalysts.^11−13^

**Figure 1:**
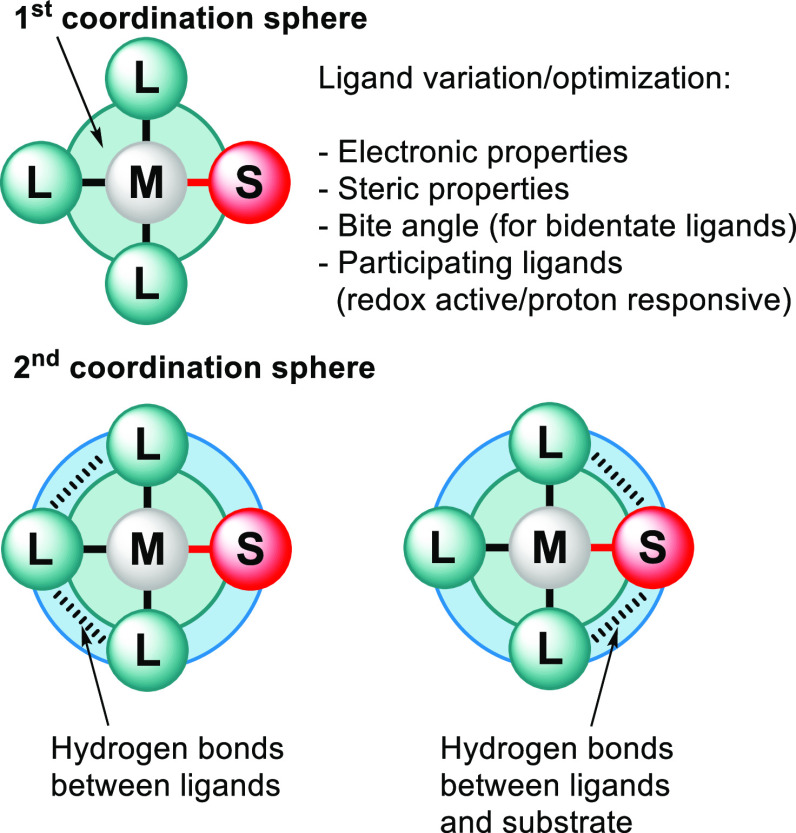
Changing catalyst parameters via the “first coordination sphere” implies changing the properties of the ligands that are coordinated to the metal complex (top) and control of catalyst properties via hydrogen bonds in the second coordination sphere (bottom). M = transition metal, L = ligand, S = substrate.

In developing new metal complexes for catalysis, activity, selectivity, and stability are crucial parameters. Especially the selectivity that a catalyst displays can be hard to control. Both enantioselectivity and regioselectivity can be very difficult to achieve, as precise control of the reaction pathways is required. Energy differences in the competing pathways induced by ligand effects as small as 3 kcal·mol^–1^ already lead to sufficient selectivity, and such effects are, thus, very subtle. Although computational techniques are far advanced, rational design of ligands for selective catalytic processes, in general, remains challenging, although some interesting examples have been reported.^14^ As a result, the search for selective catalysts often relies on trial-and-error approaches, which are facilitated by high throughput experimentation.^15^ The availability of catalyst libraries of sufficient size and diversity is required to allow rapid screening methodologies, which means that ligands preferably need to be prepared in a modular fashion using relatively simple steps.^16,17^ In practice, the search for new catalysts may be based on a combined combinatorial and rational design approach, depending on the respective challenge to be solved. What all these approaches have in common is that the properties of the catalyst are controlled via the “first coordination sphere”, i.e. tuning the properties of the ligands that coordinate to the metal, as illustrated in Figure 1. The combination of different metals from the d-block of the periodic table and a variety of ligands that are diverse in electronic properties and steric size already gives enormous potential to control catalyst properties, hence the success of metal complexes in homogeneous catalysis. Despite the successes, there remain many challenges that have not been solved. As such, new tools to control catalyst properties are continuously being developed.

One of the more recent strategies to control catalyst properties focuses on the “second coordination sphere”, i.e. the interactions beyond metal–ligand coordination that are important. These include noncovalent interactions between the ligands themselves, interactions with the ligand(s) and the substrate(s) (Figure 1), and/or interactions with the environment. Arguably, this approach is inspired by natural systems as enzymatic conversions also rely, to a very large extent, on control via residues and cofactors not *directly* involved in the chemistry of a catalytic cycle. Substrates that are docked in the pocket of enzymes near the active center are well preorganized to give the proper activation and selectivity.^18^ In redox enzymes, electrons, protons, and substrates are usually preorganized to facilitate rapid conversions.^19^ Such preorganization strategies have been intensively explored in the field of proton reduction catalysis by using ligands with internal basic functions,^20^ a topic that will be covered in this thematic issue by others. In the field of supramolecular chemistry, which mostly developed in parallel with the field of metal-based catalysis, enzymes have been a source of inspiration in the development of supramolecular catalysts.^21−26^ However, supramolecular catalysis has traditionally been very focused on relatively simple conversions, such as hydrolysis and Diels–Alder reactions, to prove that some of the concepts found in nature can be mimicked by synthetic analogues. More recently, the implementation of supramolecular strategies into homogeneous catalysis with metal complexes has been explored and demonstrated to be very powerful.^27,28^ This has resulted in novel tools to control metal catalyzed processes via the second coordination sphere, including the self-assembly of bidentate ligands using ligand building blocks, substrate orientation at the metal center via additional supramolecular interactions, and the use of molecular cages around the metal center to control catalytic reactions.

This review focuses on the application of hydrogen bonds (HBs) to control catalyst properties via the second coordination sphere (Figure 1). The organization of this review is as follows: In section 2, we provide some fundamental information on HBs and factors that influence their utility. Next, in section 3, we review the use of self-assembled bidentate ligands via HBs. Section 4 reviews the use of hydrogen bonding between the substrate and the catalyst as a way to control catalytic conversions. In transfer hydrogenation reactions, as pioneered by Noyori,^29^ the reaction can also proceed via an outer sphere mechanism in which hydrogen bonding is crucial. In these types of reaction mechanisms the HB donor typically also actively participates in the reaction by delivering the proton to the substrate. As this is a different use of the HB, it is beyond the scope of this review, and we refer the interested reader to well-established and recently published reviews.^30−41^ Also the HB-assisted activation of small molecules in redox reactions, such as the reduction of oxygen and carbon dioxide, is beyond the scope of the current review, yet we want to stress that also for these types of conversions HB interactions in the second coordination sphere can greatly affect the performance of the catalyst.^42^ Finally, we provide a summary and an outlook (section 5).

## 2 Fundamentals of Hydrogen Bonding

### 2.1 Definitions and Characteristics of Simple HBs

The concept of a hydrogen bonding interaction is more than a century old,^43,44^ has a well-documented history,^45,46^ and has been extensively reviewed.^46−49^ Some of the key characteristics of simple HBs are summarized in Table 1. A HB is typically understood as an attractive interaction between a covalently bound and positively polarized hydrogen atom and an electronegative entity. In its most simple form, a HB can be written as X–H···Y, where X stands for the donor atom and Y for the acceptor.^46,50^ In order for the hydrogen atom to be positively polarized, the donor atom must be more electronegative, which is the case for most main group elements. The acceptor moiety has to be electron rich and typically involves a lone pair of electrons from an atom or anion.^46,50−52^ Other less-typical^53,54^ HB acceptors include π-electrons,^55−60^ some transition metals,^61−64^ and even hydrides.^65^

**Table 1:** Some Guideline Characteristics of a Single [X–H···Y] Hydrogen Bonding Interaction Based on the Review in Section 2.1 and Adapted from Ref (48)

	Strong	Moderate	Weak
Δ*E* (kcal·mol^–1^)^a^	15–45	4–15	<4
Directionality	Strong	Moderate	Weak
X–H vs H···Y distance^b^	X–H ≈ H···Y	X–H < H···Y	X–H ≪ H···Y
X–H···Y angle (deg)^c^	170–180	>130	>90
IR Δ*ν̃*_X–H_^red-field^ (% of cm^–1^)	>25	10–25	<10
^1^H NMR Δδ^downfield^ (ppm)	14–22	<14	
Typical driving force	Orbital and/or electrostatic interaction	Electrostatics	Dispersion

a Estimated values from calculations in the gas phase, roughly synonymous with the enthalpy of formation, Δ*H* (entropy is often ignored; see also section 2.2).

b Exact values of these distances are highly dependent on the van der Waals radii of the elements involved.

c Values only apply to singular HBs, not bifurcated and more complex structures.

The strength of a simple HB thus depends on the nature of X and Y, and the bond is strengthened when X is more electron withdrawing and/or when Y is more electron dense. Charge-assisted HBs, where X–H is cationic and/or Y is anionic, are particularly strong.^66,67^ It is thus no surprise that the interaction energies of simple hydrogen bonding interactions can vary greatly. The interaction energy between the very weakly polarized C–H of methane and the π-bond of, e.g., ethylene is approximately −0.7 kcal·mol^–1^.^48,55,68^ Such weak interactions represent the under-boundary of what can be interpreted as a hydrogen bonding interaction and are typically driven by dispersion.^55,68−70^ While an energy of −0.7 kcal·mol^–1^ is very small, it must be noted that a difference of 0.5 kcal·mol^–1^ in a transition state has been reported to impact selectivity in catalysis.^71^ HBs with more polarized hydrogens, such as in amines, amides, and alcohols, are much stronger and more common. For example, the interaction energy of the water dimer is about −5 kcal·mol^–1^.^72,73^ Such interactions are typically driven by electrostatics, and their strength can also be anticipated by a simple inspection of the molecular electrostatic potential maps (MEPs) of the HB donor and acceptor.^74−78^ The MEP of a molecule can even be obtained with computationally very cheap semiempirical calculations for a qualitative estimation, and such calculations have been correlated to more accurate interaction energies.^79^ Electrostatic interactions are often dominant in HBs, and HBs have often been recognized as a particular type of “σ-hole” interaction,^75,80,81^ much like halogen-,^82^ chalcogen-,^83^ pnictogen-,^84^ and tetrel bonding^85^ interactions.

In its most extreme form, the outcome of a hydrogen bonding interaction can be a proton transfer reaction, where the donor donates a proton to the acceptor via a formally hypervalent [X–H–Y] species.^86,87^ In the end result of such a reaction, the original donor has become the acceptor and *vice versa*. As can thus be expected, the p*K*_a_ of the donor and p*K*_b_ of the acceptor are correlated with the energy of the HB formed between them.^79,88^ The proton transfer component also rationalizes the linear directionality of HBs^58,59,74,89−95^ as well as the elongation of the X–H and shortening of the H···Y bond in strong HBs observed in crystal structures.^96−101^ The orbital component of HBs can be seen as donation of the electron density of a lone-pair (*n*) on Y into the antibonding orbital of an X–H σ bond, typically written as *n* → σ*.^81,86^

In rare cases where the HB is very strong and symmetrical, the hypervalent species might actually be a stable compound. For example, the [F–H–F]^−^ anion has a “hydrogen bonding” interaction energy of approximately −40 kcal·mol^–1^,^86^ which is on par with a weak covalent bond such as the peroxide σ bond in (CH_3_)_3_CO–OH (−47 kcal·mol^–1^)^102^ and represents the upper-boundary of a single HB.

The bonding strength of a simple HB actually correlates well with the covalent character of the interaction, which can be deduced from the electron density of the H···Y bond.^46,70,103−107^ This density can be calculated and is often expressed by using the density of bond critical points of Baders’ quantum theory of atoms in molecules (QTAIM),^108^ which indeed correlates well with the binding energy of a HB.^95,109−111^

Information about HBs can be gathered using various computational tools (energies and geometries, mostly in the gas phase),^70,74,86,87,112^ and data from crystal structures provide direct evidence for geometric characteristics and directional preferences.^58,59,68,90,91,94,113−117^ In solution, which is most relevant to catalysis, infrared (IR)^48,60,118−122^ and nuclear magnetic resonance (NMR)^48,119,122−129^ spectroscopy are common techniques to evaluate the presence and binding strength of a HB. The IR spectroscopy stretching vibration of the X–H bond (*ν̃*_X–H_) is particularly informative and typically displays a red-shift, broadening and/or intensifying when involved in a HB. In strong HBs, the shift of *ν̃*_X–H_ can be as large as 2,500 cm^–1^.^118,120^ Formation of a HB also has a significant effect on the NMR spectroscopic properties of a proton (and the atoms it is in contact with, X and Y). ^1^H NMR spectroscopy is, therefore, routinely used to evaluate hydrogen bonding, and downfield shifts exceeding Δδ = 20 ppm have been observed for strong HBs.^125^

### 2.2 Factors Influencing the Utility of a HB

A most obvious manner to influence the interaction energy of a simple X–H···Y HB is to adjust the electronic properties of X and/or Y.^46,50^ In general, for a stronger HB, X has to be more electron withdrawing and Y more electron rich. The properties of X and Y can partially be tuned by choosing the atoms; a nitrogen is more polarizing than a carbon. The hybridization of the donor atom and its further chemical context can also have a large effect on the electrostatic potential on H in the X–H donor. For example, amides are far better HB donors than amines, and carbonyls are superior HB acceptors compared to alcohols.^130−133^ Using electron withdrawing groups (e.g., a nearby positive charge) can even render an otherwise fairly unpolarized C–H bond into a functional HB donor.^56,57,134−143^

Computations under idealized gas-phase conditions indicate that the interaction energies of a single HB can be up to −45 kcal·mol^–1^.^48,144^ However, such computed interaction energies are best seen as *de facto* enthalpies (Δ*H*), not Gibbs free energies (Δ*G*). Indeed, solution-phase experimental evidence reveals that the Δ*G* of a single HB is at most approximately −10 kcal·mol^–1^.^122^ This 4–5 factor difference between computed (gas-phase) interaction energies and observed Gibbs free energies can in part be understood by the role of the solvent on binding.^79,145−149^ For example, charges that strengthen a HB in the gas phase are more diffuse when in solution, with the consequence that gas-phase calculations overestimate the reinforcement of a charge on the enthalpy of a HB.^150−154^ Furthermore, in solution both the HB donor and acceptor will be solvated and association into the intended HB complex will have to overcome this solvation. For example, the interaction energy of perfluoro-*tert*-butanol hydrogen bonded with tri-*n*-butylphosphine oxide *in vacuo* can be calculated at −19 kcal·mol^–1^ (DFT/B3LYP-D3/def2-TZVPPD), while Δ*G* has been measured as merely −4.68 kcal·mol^–1^ in CDCl_3_ (*K*_a_ = 2700 M^–1^).^145^ Hydrogen-bonded complexes are thus typically strongest in apolar aprotic solvents such as alkanes and weakest in polar protic media such as water or methanol.^79,149^

In addition to the solvent effects on the enthalpy, the entropy component (−*T*Δ*S*) can have a profound impact on the Gibbs free energy. There is an obvious translational entropy penalty of bringing two entities together to form a HB complex, and the magnitude of such a bimolecular association has been estimated to be about 3–9 kcal·mol^–1^ for simple molecules in solution.^79,155−157^ Moreover, the conformational freedom can be expected to diminish when a HB complex is tightly bound.^158,159^ Such a reasoning can explain the often observed inverse proportional relationship between the enthalpy and entropy of formation measured for an adduct.^158−164^ While often observed, this “enthalpy–entropy compensation” cannot be considered a general feature of molecular associations.^165−168^ In some instances, entropy can be a substantial driving force of binding, especially when a guest can replace several entropically confined solvent molecules from a binding site.^169−172^ A similar rationale can be applied to the entropy component of the hydrophobic effect.^170,173,174^

The entropy component of a HB can be markedly different when a HB is established within the same molecule, as there is no loss of translational entropy.^175−177^ It is thus unsurprising that *intra*molecular HBs can be stronger than *inter*molecular HBs with a similar donor and acceptor, especially if the donor and acceptor are nearby in a conformationally rigid molecule.^178−181^ Entropy aside, intramolecular HBs have very similar characteristics as their more frequently studied intermolecular counterparts. For example, solvation also tends to weaken intermolecular HBs,^147,182^ they display similar directionality,^95^ and computational analysis has shown that the electronic density of the H···Y bond correlates with the strength of the bond.^110,183^

**Scheme 1:**
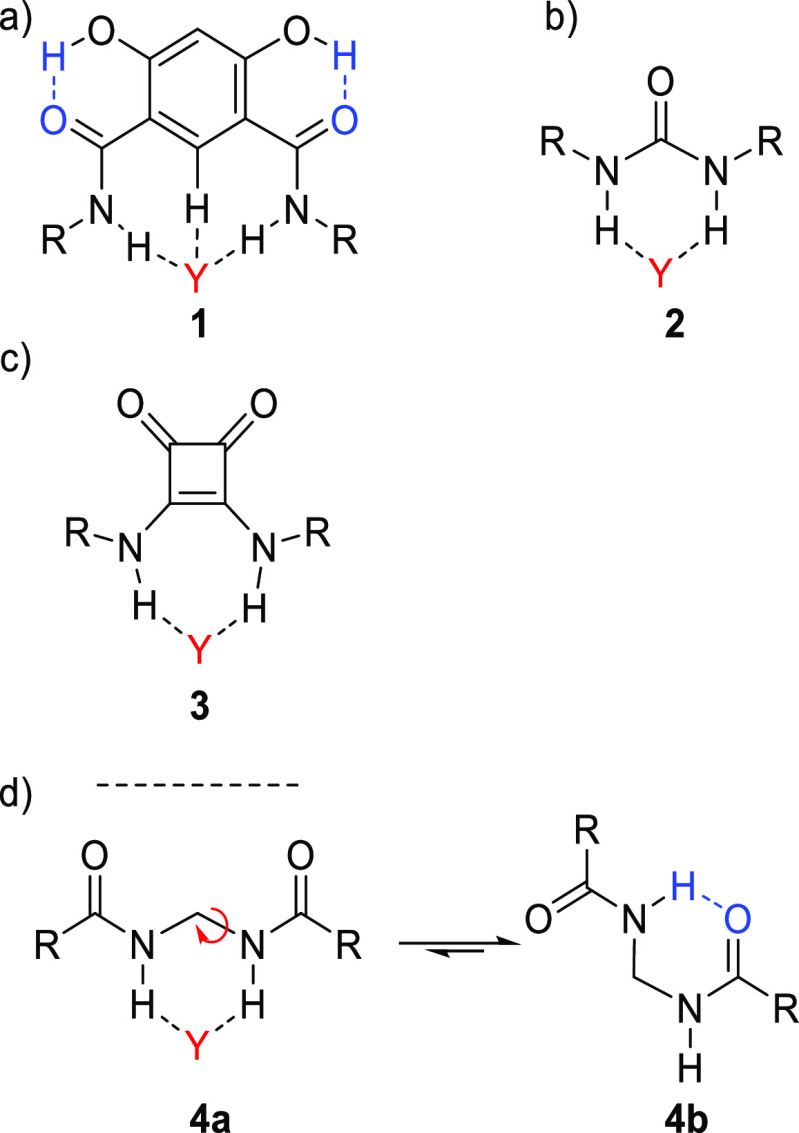
Example of Intramolecular HBs (in Blue) and Multipronged HB Donor Groups: (a) an Isophthalamide Derivative (**1**) Where Both Amides Have Been Preorganized by an Intramolecular O–H···O HB;^193−195^; (b) General Structure of a Urea Bifurcated HB Donor (**2**);^196,197^ (c) General Structure of a Squareamide Bifurcated HB Donor (**3**);^198,199^ (d) Diamide **4a**, Which Might Be a Bifurcated HB Donor Similar to a Urea but Where an Intramolecular HB Will Preorganize the Diamide into a Different Energy Minimum Conformer (**4b**)^140^ The isophthalamide can also be seen as a trifurcated HB donor (include the CH). R can be any substituent. Y stands for the acceptor.

A useful feature of intramolecular HBs is the possibility to program the conformation of a molecule to steer its structural (pre)organization. This option has been copiously exploited by nature in, for example, protein folding^57,184,185^ and in the stabilization of transition states that often make proteins such good catalysts.^22,24,186−189^ The concept of intramolecular preorganization with HBs has also been utilized in crystal engineering,^182^ in medicinal chemistry,^190^ and in the design of receptor binding pockets.^67,177,191−193^ For example, as is illustrated in Scheme 1a, the amides in isophthalamide (**1**) can be preorganized by intramolecular O–H···O HBs (in blue).^193−195^ This preorganized structure was shown to bind an order of magnitude more strongly to halide anions compared to an analogue that lacks the two alcohols and is thus not preorganized.^193^

The isophthalamide structure shown in Scheme 1a also illustrates that multipronged^200^ HBs will lead to more stable adducts compared to a simple single X–H···Y HB.^48,201−205^ In this instance, isophthalamide (**1**) can be seen as a “bifurcated” HB donor when counting only the amidic H’s but as a “trifurcated” HB donor when also counting the central CH as a HB donor. Other well-known and often used examples of bifurcated HB donors are the ureas (**2**)^196,197^ and squareamides (**3**)^198,199^ shown in Scheme 1b and c, respectively.

It must be noted that preorganization using intramolecular HBs can also be a disadvantage. For example, as is illustrated in Scheme 1d, one might envisage that a decent bifurcated HB donor such as **4a**—very similar to a urea—can be obtained, if two amides are N-linked by a methylene. However, such a motif will result in structure **4b**, which is stabilized by intramolecular hydrogen bonding, and the anticipated bifurcated motif will not be the most stable conformer.^140^ The detrimental effect that intramolecular hydrogen bonding can have on the preorganization of a binding pocket is well-documented, for example in cholic acid derived anion binders.^206^

**Scheme 2:**
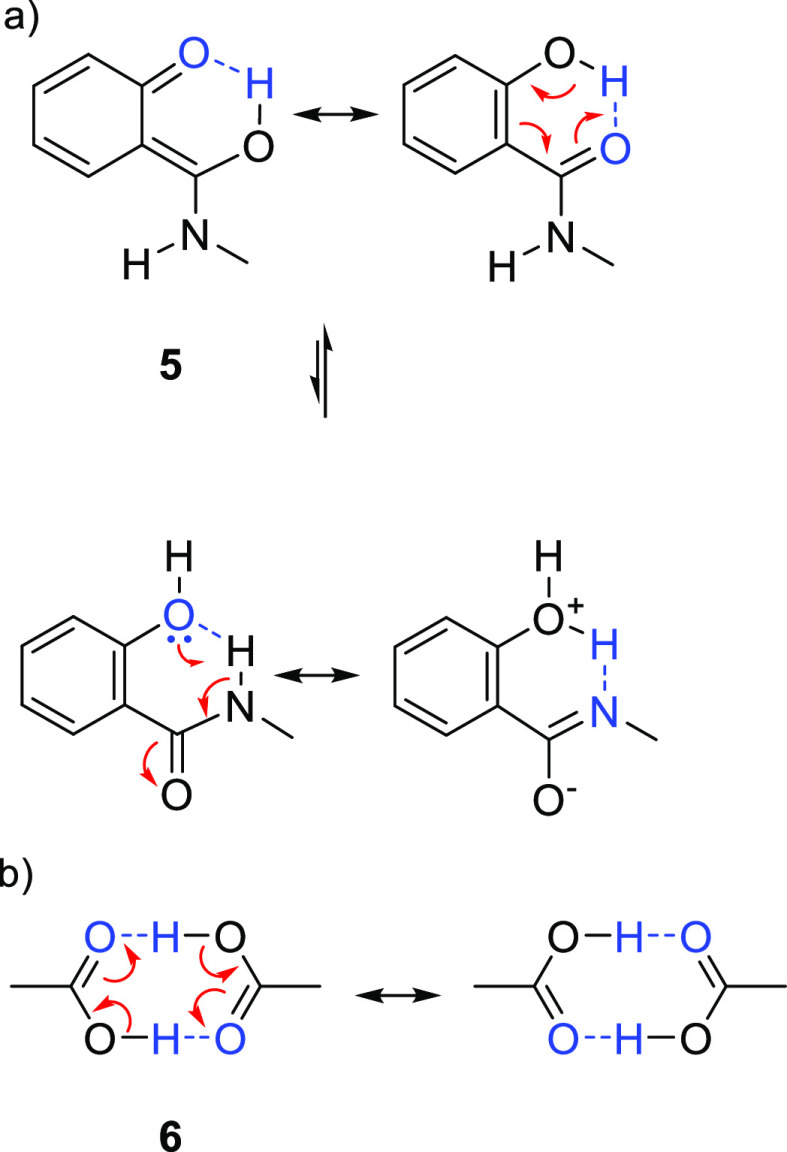
Examples of Resonance Stabilized HBs: (a) 2-Hydroxy-*N*-methylbenzamide (**5**) Is Stabilized by an Intramolecular Resonance-Assisted HB;^198^ (b) an Interesting Feature of Both Intermolecular and Multipronged HBs Is That They Form a Ringlike Structure as Found for Carboxylic Acids (**6**)

For many multipronged HBs as well as for some intramolecular HBs, it is possible to envisage tautomers based on simple Lewis structures. It has been noted that the possibility of resonance structures can have a stabilizing effect on inter-^207^ and intramolecular HBs.^208^ For example, drawing tautomers of 2-hydroxy-*N*-methylbenzamide (**5**) (Scheme 2a) can rationalize why the intramolecular O–H···O=C HB conformer (top) is about 6.4 kcal·mol^–1^ more stable^198^ than the N–H···OH HB conformer (bottom): in the latter, proton transfer would lead to a species with a formal separation of charges.^70^*Inter*molecular resonance-assisted HBs include carboxylic acid dimers (**6**) (Scheme 2b).^207,209^ The phenomenon has also been described as a stabilizing factor for the secondary structure of proteins^210^ and in base pairs.^211,212^

**Figure 2:**
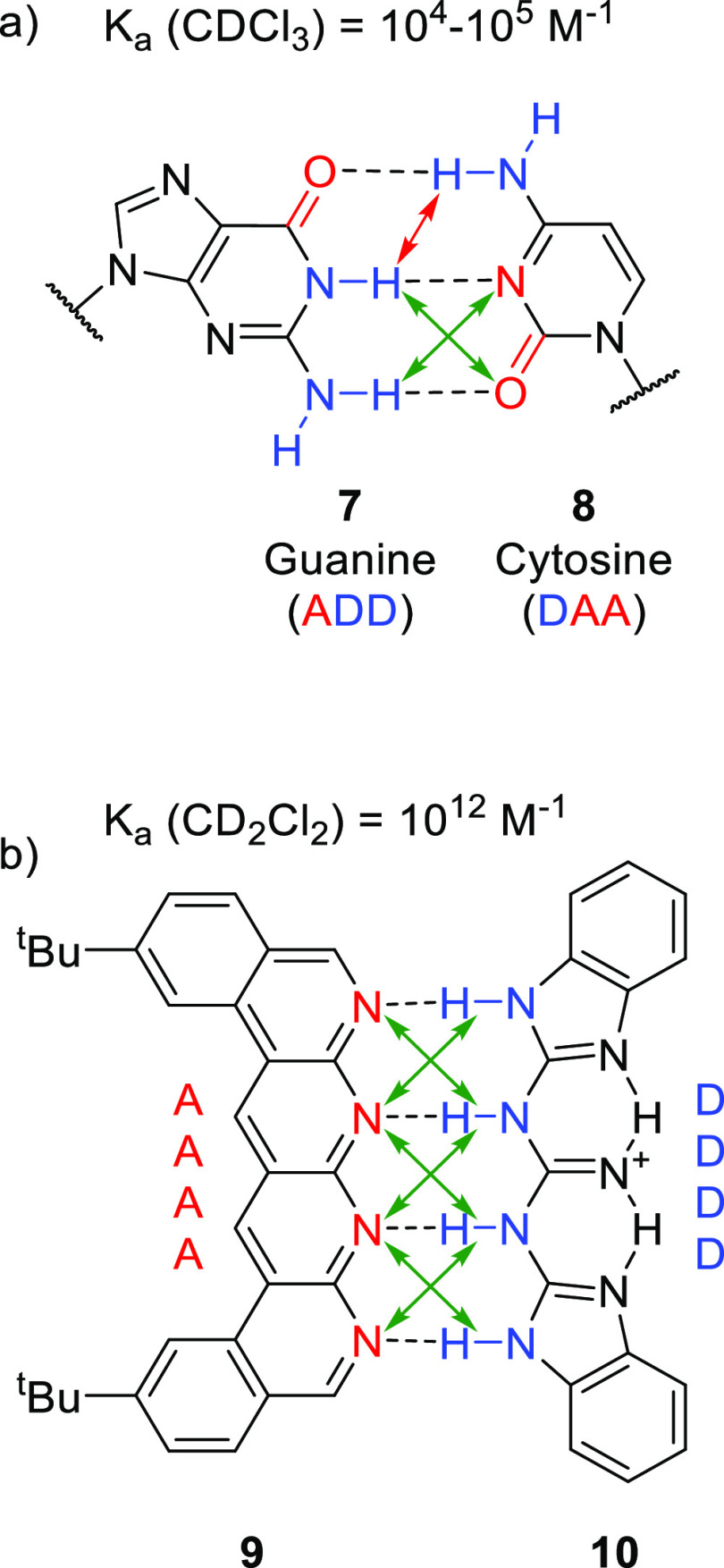
HB arrays with indication of attractive (D↔A) and repulsive (can be D↔D or A↔A) secondary interactions (a) found in nature between the base pairs guanidine (**7**) and cytosine (**8**)^213,214^ or (b) as implemented in a synthetic system from **9** and **10** with exceptionally high binding affinity exceeding 10^12^ M^–1^.^217^

When multiple HB donors and/or acceptors form an array, such as for carboxylic acid dimers (**6**) (Scheme 2b), there can be secondary electrostatic interactions^70,213^ between adjacent donor (D) and acceptor (A) moieties. Note that such arrays are distinct from multipronged HBs, as each HB donor is complemented by one HB acceptor. This is illustrated for the guanine-cytosine base pairs (7–**8**)^213,214^ in Figure 2a, where the repulsive secondary interactions are indicated with red arrows (D↔D, but could also be A↔A) and attractive interactions with green arrows (D↔A). The concept of secondary interactions has been used to design synthetic heterodimeric^215−217^ and homodimeric^218,219^ systems (e.g., self-complementary AADD^218^ or ADAD^219^ HB arrays). As an example, Figure 2b shows an exceptionally stable AAAA–DDDD quadruple HB array (**9**–**10**) (*K*_a_ > 10^12^ M^–1^ in CD_2_Cl_2_), where all the secondary interactions are attractive.^217^

**Scheme 3:**
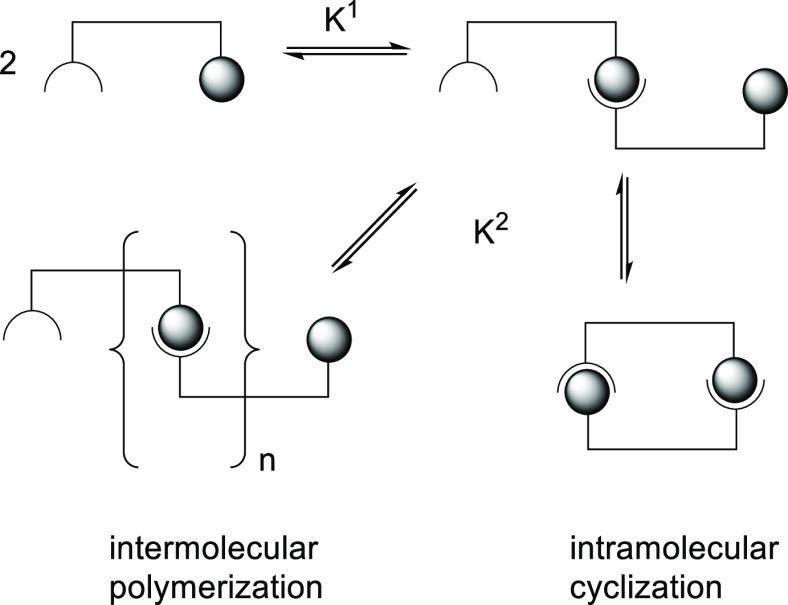
Illustration of So-Called “Chelate-Cooperativity” That Occurs in Multivalent Molecules

Cooperativity can be defined as the interplay between two or more interactions that cause a system as a whole to behave differently than what might have been anticipated based on the properties of isolated individual interactions.^220^ Several elements of cooperativity relevant for a binding site consisting of HBs have already been introduced: multipronged HBs, resonance stabilization, HB arrays, and secondary interactions. However, when a molecule consists of multiple separate binding sites, the molecule can be described as multivalent.^221^ Multivalency can lead to an additional type of cooperativity, which has been referred to as “chelate-cooperativity”.^220,222−224^ A most basic example is illustrated in Scheme 3 and involves a bivalent self-complementary molecule that can form a dimer or polymer, depending on the preference for intra- or intermolecular bonding of the second binding event (*K*^2^).^220−222^ The space in between the two binding sites is known to have a large effect on the intra- versus intermolecular association by virtue of the enlarged “effective concentration”^221^ that the second binding event enjoys.^221,224^ When designing a binding pocket, such self-complementarity is best avoided. Multivalent cooperativity has been used to generate a large variety of structures based on HB assemblies,^225,226^ and it has been exploited to make supramolecular polymers.^218,227−229^ The cooperativity of multivalent binding is copiously exploited in nature,^230^ also using HBs, such as in the canonical double helical Watson–Crick structure of DNA,^207^ in protein folding,^57,184,185,189^ and in the cooperative effects that have been noticed in water clusters.^231^

### 2.3 HBs to Control the Second Coordination Sphere

In this section we have detailed the characteristics of basic HBs and the factors that influence their utility, including more complicated HB structures. The utility of HBs to control the catalyst properties in the second coordination sphere will be detailed in the next two sections. From these surveys, it will become apparent that most examples deploy rather typical HBs of a classical type.

That this presents a clear opportunity is underscored by the fact that some of the most successful examples, whether intended or not, actually rely on one or more of the fine-tuning parameters highlighted in the previous section. For example, the success of P=O bonds (e.g., section 3.1.1, Figure 6, and Scheme 39) can be seen as an example of electronic tuning as P=O bonds are highly polarized and are thus among the best known (neutral) HB acceptor groups.^79,145^ The impact of solvation and entropy on the utility of HBs to control the second coordination sphere is reflected in the typical choice for noncompetitive (apolar aprotic) solvents such as dichloromethane (DCM), tetrahydrofuran (THF), and alkanes found throughout this review. At the same time, some systems can operate in much more competitive solvents and at higher temperatures (e.g.,Scheme 301, and section 3.2.2.1). It is no surprise that such systems actually rely on strong *intra*molecular HBs. Similarly unsurprising is the success of catalyst control using multipronged HB donor units derived from ureas (section 3.2.1 and Figure 16), “DIMPhos” (Figure 9), and acyl guanidine (Figure 15). The utility of HB arrays is evident by the examples highlighted throughout this review (e.g., Schemes 22, 23, 29, 33–38, and 62 and Figures 20 and 21), although it must be noted that most of the secondary interactions in these examples are repulsive (which provides a clear opportunity to redesign these structures). Resonance stabilized structures have been used, although sparingly, such as the “6-DPPon” structure (**135** shown in Scheme 29). The concepts of structure preorganization with HBs, multivalency, and cooperativity do not yet seem to have been utilized in the catalyst control of the second coordination sphere.

The presence of HBs in a metal complex, between the ligand building blocks or between the ligand and the substrate, can be established by a variety of techniques, including X-ray analysis and NMR and IR spectroscopy. Typically, the signals of the hydrogen atoms involved in hydrogen bonding are shifted in both the NMR spectrum and the IR spectrum, and the same holds for the HB acceptor. These signals can be used as a probe, and from titration studies, the HB strength under the used conditions can be established. To what extent the HB in the second coordination sphere controls the activity and the selectivity is more difficult to probe, and typically this information is obtained by comparison of catalysis results with proper control experiments. Job plot analysis is frequently used in supramolecular chemistry to reveal the stoichiometry of the complex, and this could also be used to get insight into HB-containing metal complexes. Extension to information on the active species has been explored by kinetic Job plots, in which the reaction rate is plotted against the fraction of components.^232^ To the best of our knowledge, such an approach has not been used to evaluate supramolecular bidentate ligands (section 3) or supramolecular catalyst systems that operate via substrate orientation (section 4), but such experiments may provide additional information. Related experiments that have been reported involve a supramolecular bidentate ligand, in which one of the two components is added in increasing amounts, probing the activity and selectivity. These experiments showed in this particular case that the supramolecular ligand was present, even in the presence of an excess of one of the building blocks.^233^

The above identified examples (detailed in the sections to come) underscore that there is much potential to improve the manipulation and study of hydrogen bonding phenomena in the second coordination sphere. It is thus interesting to keep in mind while reading the next sections that the full potential of hydrogen bonding interactions, using all the known tricks that influence their utility, has not been used yet. For example, HB arrays such as those displayed in Figure 2 could be used to generate supramolecular bidentate ligands. Such motifs will lead to strong bonds between the ligand building blocks, and it is anticipated that this will lead to robust systems that can be applied in polar competitive solvents. The synthesis of such building blocks, however, may be challenging.

## 3 Hydrogen-Bonded Supramolecular Multidentate Ligands

Bidentate ligands hold a privileged place in most homogeneous transition metal catalyzed reactions as they often yield higher activity and selectivity. However, preparation of large and diverse libraries of bidentate ligands often requires tedious synthetic efforts. To circumvent this challenge, supramolecular bidentate ligands can be used which are functionalized monodentate ligands that self-assemble *in situ* into a bidentate ligand using noncovalent interactions. Among the different noncovalent interactions, such as coordination and ionic bonds, hydrogen bonding has been frequently used, as it has several favorable characteristics, including (a) predictability, (b) directionality, (c) dynamic bonding, (d) tunability, and (e) various HB donor and acceptor synthons being synthetically accessible.

All those factors led to the rapid development of supramolecular bidentate ligands based on hydrogen bonding in the last decades. Systems based on both single HBs and HB arrays have been reported. This part of the review deals with hydrogen-bonded supramolecular bidentate ligands used in transition metal catalysis. We focus exclusively on reports where experimental evidence is provided for the relevance of hydrogen bonding in a precatalytic state or during catalysis. It is also worth mentioning that sometimes no clear distinction between a hydrogen-bonded supramolecular bidentate ligand and substrate orientation by hydrogen bonding (section 4) can be drawn, as in some specific cases the substrate intercalates into the ligand’s HB network during the catalytic cycle. Those cases will be reviewed in both parts in line with the focus of the section.

### 3.1 Supramolecular Ligands Using a Single HB

#### 3.1.1 Secondary Phosphine Oxides

**Scheme 4:**
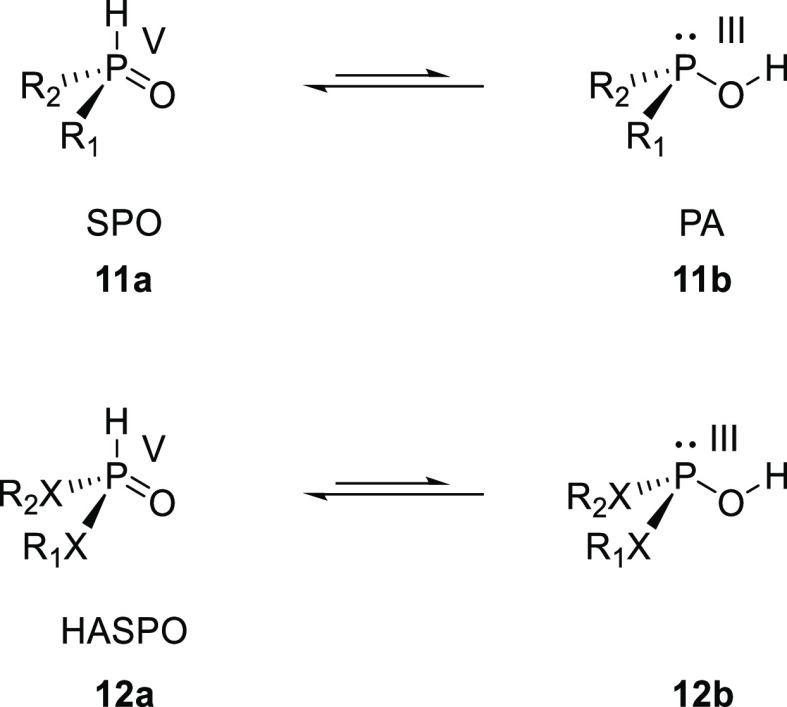
Tautomerism of (HA)SPO Ligands: (**11**) R_1_, R_2_ = alkyl or aryl; (**12**) X = O or NR

Secondary phosphine oxides (SPOs) (**11a**) are weak acids and subject to tautomerism (see Scheme 4). SPO (**11a**) is a pentavalent phosphorus oxide, while its tautomer is a trivalent phosphinous acid (PA) (**11b**). The equilibrium depends on the electronic properties of the SPO, and strongly electron withdrawing substituents on the phosphorus shift the equilibrium toward the trivalent PA form. As the pentavalent oxide form normally predominates, the SPO ligands are air and moisture stable. At the same time, metal coordination can shift the equilibrium toward the trivalent PA tautomer, forming metal–phosphinous acid complexes with a metal-to-phosphorus bond of comparable strength as typical metal–phosphine bonds. The ability to coordinate with both the phosphorus and oxygen creates a rich coordination chemistry of mononuclear and multinuclear complexes of this class of compounds.^234^ Structural diversification of this class of ligands is possible by exchanging the alkyl or aryl groups, for alkoxy or amide groups which are referred to as heteroatom secondary phosphine oxide (HASPO) ligands (**12**).

**Scheme 5:**
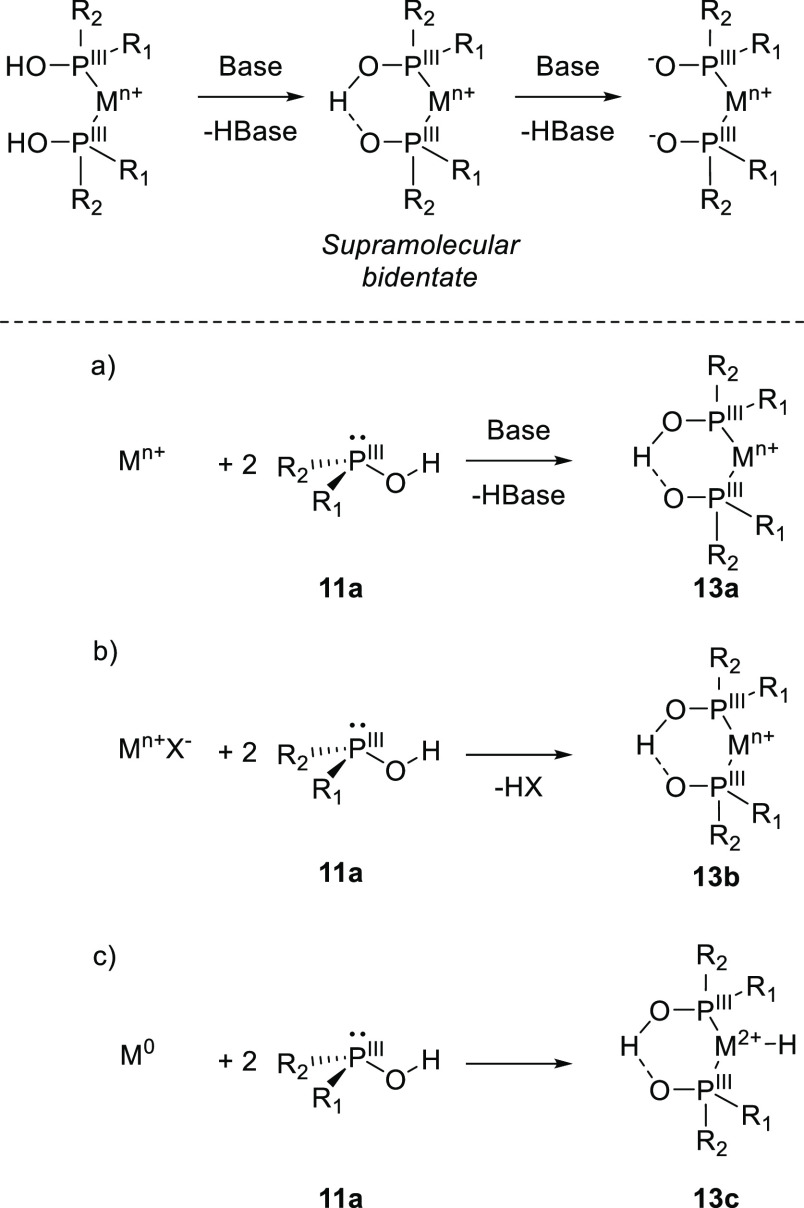
Formation of Supramolecular Hydrogen-Bonded Bidendate Ligands Using SPO Ligands Synthetic approaches involve (a) the use of an external base to deprotonate one SPO, (b) the use of an internal base, or (c) formation of an anionic SPO via oxidative addition. X^–^ can be OAc^–^ or OMe^–^. In structures **13a**–**c**, the HB exchanges between the two oxygen atoms.

One particular feature of (HA)SPO ligands is that they form hydrogen-bonded supramolecular bidentate and tridentate ligands, as first reported in 1975.^235^ In such a case, both SPO ligands are in the P(III) state and, therefore, have a lone pair of electrons to coordinate to the metal center.^236^ The addition of 1 equiv of base deprotonates one P–OH, leading to the formation of the H-bond acceptor for the anionic supramolecular bidentate (see Scheme 5). Although different Lewis structures are reported, even within single reports, X-ray crystal structures and DFT optimized structures show that both SPO ligands are in a P(III) state.^237−241^ For clarity, throughout this review the covalent bond is indicated with a solid line and the HB is indicated by a dashed line. In an anionic supramolecular bidentate of SPO ligands, the HB (and the covalent O–H bond) exchanges between the two oxygen atoms.^242^ The SPO–bidentate complexes (**13a**–**c**) [M{(PR_2_O)_2_H}] can be formed by the three different routes detailed in Scheme 5: (a) mixing 2 equiv of SPO (**11a**) and a metal precursor in the presence of base, (b) mixing 2 equiv of SPO (**11a**) and a metal precursor containing a basic ligand (such as acetate or methoxide), or (c) mixing a low valent metal precursor with 2 equiv of SPO (**11a**). For routes a and b, the metal valency remains unchanged, while in method c, the metal center is oxidized, with the concomitant formation of a catalytically relevant metal-hydride species.^243^ Such a reaction does not require any additional anions, providing additional stability of these complexes. The participation of the P–O–H···O–P six-membered cycle in bonding and reaction mechanisms shows the bifunctional nature of these catalysts, which has been reviewed elsewhere.^244^

##### 3.1.1.1 Hydroformylation

**Scheme 6:**
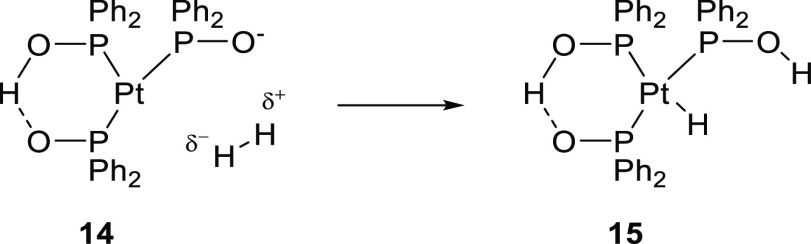
SPO-Assisted Hydrogen Activation

The first application of SPO ligands in metal catalyzed reactions dates back to the 1980s when van Leeuwen and co-workers reported the Pt/SPO-hydride catalyzed hydroformylation reaction. The active catalyst (**15**) is formed by mixing Ph_2_POH and Pt(COD)_2_ (COD = 1,5-cylooctadiene) under hydroformylation conditions, yielding [PtH(PPh_2_OH){(PPh_2_O)_2_H}] (**15**, see Scheme 6). Complex **15** catalyzes the conversion of 1- and 2-heptenes to the corresponding linear aldehydes with selectivities of 90% and 60%, respectively.^245^ These regioselectivities are in the range of traditional bidentate ligand systems such as Xantphos and demonstrate that the hydrogen-bonded bidentate structure remains intact at the reaction temperature 100 °C. Calculations indeed confirm the additional stabilization by the hydrogen-bonded bidentate by 13.0 kcal·mol^–1^.^246^ The hydroformylation of ethylene with **15** produces the typical product propanal but also, unexpectedly, the hydroacylated product pentan-3-one, formed by a second ethylene insertion into the Pt–acyl bond. The authors pointed out that the SPO ligands have peculiar properties, capable of assisting in the activation of dihydrogen, which is considered to be the bottleneck in Pt catalyzed hydroformylation (Scheme 6).^244,247^ Detailed DFT studies suggest that the supramolecular bidentate ligand is maintained throughout the catalytic cycle and that the proton in the P–O–H···O–P six-membered cycle readily migrates between the two oxygen atoms, providing fine-tuning of the electron density in the catalytic cycle at each reaction step.^244,248^

**Scheme 7:**
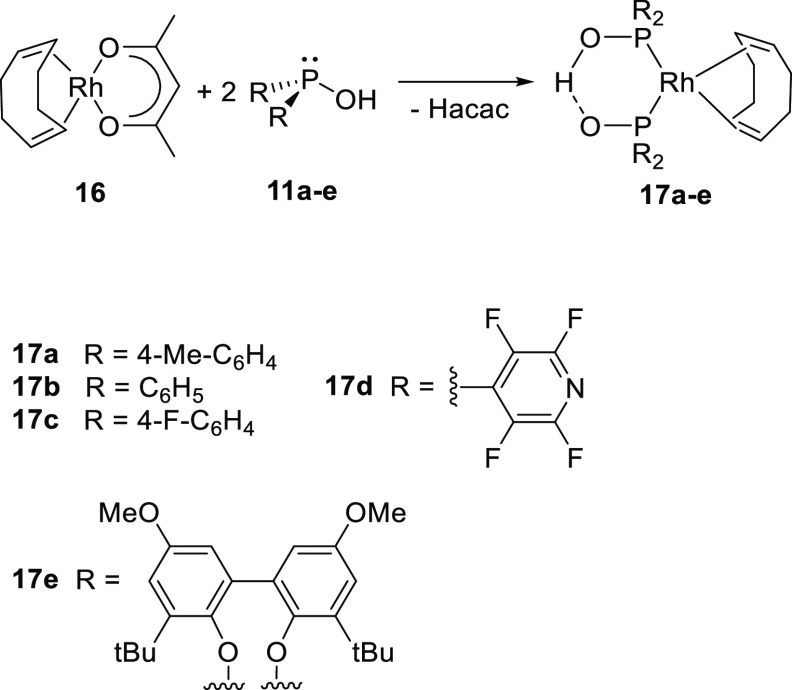
Formation of Rh-(HA)SPO Catalysts (**17a**–**e**) Used for the Hydroformylation of 1-Octene

Much later, the interest in rhodium/SPO catalyzed hydroformylation was initiated by the work of Börner and co-workers.^249^ They studied a limited library of SPO ligands with different electronic properties. The supramolecular bidentate ligand was formed by mixing [Rh(COD)(acac)] (**16**) with 2 equiv of ligand (**11a**–**e**) in which the acac ligand acts as an internal base (Scheme 7, acac = acetylacetonato). SPO ligands with electron withdrawing substituents react rapidly (at −78 °C), while the electron-rich di(*tert*-Bu)phosphine oxide yields only traces of the [Rh(COD){(*tert*-Bu_2_PO)_2_H}] complex at 80 °C, reflecting the tautomeric equilibrium (not shown in Scheme 7).^250^ Hydroformylation reactions with complexes **17a**–**d** were performed using cyclohexene and 1-octene. The substrate cyclohexene was readily converted to the aldehyde with yields up to 50%. These results are superior to the benchmark Rh/PPh_3_ catalyst, yielding only 18% under identical conditions with cyclohexene as substrate. In contrast to traditional hydroformylation catalysts that are more active when the ligands are electron poor, more electron-poor SPO ligands resulted in only 7% conversion. Using 1-octene as substrate, the aldehydes were produced in 88% yield, albeit with a moderate selectivity for linear aldehydes due to considerable olefin isomerization. The rhodium-HASPO catalyst (**17e**) was evaluated in the hydroformylation of 1-octene, providing 91% conversion to the aldehydes, of which 38% favor the linear aldehyde (in THF, 100 °C, 50 bar syngas (CO/H_2_ = 1:1), Rh/olefin = 1:8000, b/1 = 1:4).^251^

**Scheme 8:**
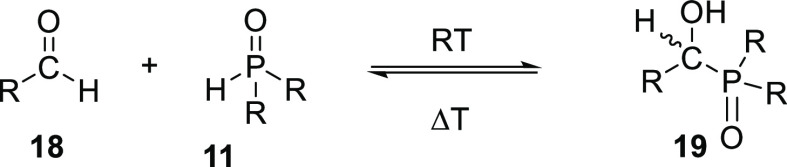
Reaction between Aldehydes **18** and SPO **11** (as the Acid Tautomer) to Produce α-Hydroxyphosphine Oxides **19**

Due to the acidic P–H, (HA)SPOs can add to olefins or aldehydes via their PA tautomer to produce α-hydroxyphosphine oxides by an Abramov or Pudovik reaction which is reversible at elevated temperatures and thus forms a reservoir of SPO ligands (**11**) (Scheme 8). It was shown that the SPO ligands are liberated during product distillation and act as a stabilizing ligand for the rhodium catalyst, improving the thermal stability and recyclability of the precious metal catalyst.^252^

##### 3.1.1.2 Hydrogenation

**Scheme 9:**
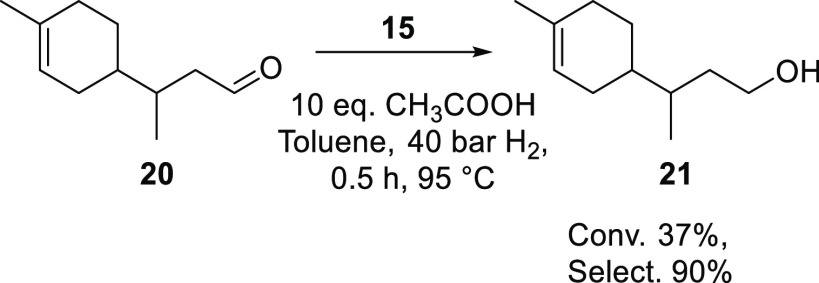
Aldehyde Reduction by Catalyst **15** (See Also Scheme 6)

The hydrogenation of aldehydes by metal-SPO catalysts has been a topic for both academic and industrial groups. An early patent by Shell describes the use of [PtH(PPh_2_OH){(PPh_2_O)_2_H}] catalyst (**15**) for the hydrogenation of a range of aldehyde and ketone substrates with high activity and high chemoselectivity.^253^*tert*-Amyl aldehyde **20** can be efficiently converted to the corresponding alcohol **21** with a turnover frequency (TOF) of 9000 mol_substrate_/mol_catalyst_·h^–1^. Interestingly, both the hydroformylation reaction and the aldehyde hydrogenation reaction are catalyzed by the same catalyst (**15**), and this potentially opens the pathway to perform the sequential hydroformylation/aldehyde reduction in a one-pot procedure (Scheme 9).

**Scheme 10:**
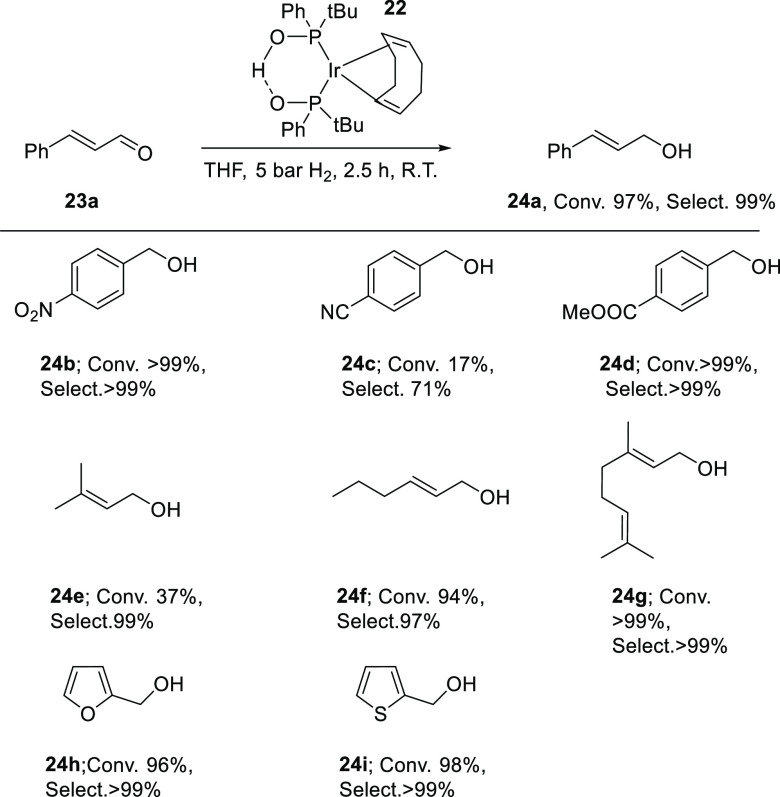
Aldehyde Reduction by Ir-SPO Catalyst (**22**)

Iridium-SPO catalysts have also been applied in the chemoselective aldehyde hydrogenation. Treatment of the (COD)(methoxy)iridium(I) dimer with 4 equiv of *tert*-butyl(phenyl) phosphine oxide and 2 equiv of water in THF at room temperature affords supramolecular bidentate complex [Ir(COD){(P(*t*-Bu)PhO)_2_H}] (**22**, Scheme 10).^254^ This complex is the precursor to a catalyst that forms under 5 bar hydrogen atmosphere leading to a mixture of monohydride, diastereomeric dihydrides, and three bridging dihydride dimer complexes.^255^ Interestingly, the oxidative addition of dihydrogen to the Ir(I)-SPO complex (which contains the achiral SPO) is highly stereoselective, as all generated Ir(III) hydride complexes are homochiral and no *meso* isomers are detected.

As is shown in Scheme 10, deploying **22** as catalyst precursor gave highly chemoselective conversions of a variety of aldehydes using mild reaction conditions (25 °C and 5 bar hydrogen pressure). The reduction of the aromatic *α,β*-unsaturated cinnamaldehyde (**23a**) to the cinnamyl alcohol (**24a**) could even be established, thus leaving the C=C double bond untouched, with >99% selectivity and a TOF > 2000 mol_substrate_/mol_catalyst_·h^–1^. *p*-Nitrobenzaldehyde (**23b**) is selectively converted to nitrobenzyl alcohol (**24b**) with perfect retention of the nitro group (selectivity >99%). Also, other groups such as nitrile (**23c**) and ester groups (**23d**) are well tolerated, as these substrates are converted with nearly perfect chemoselectivity. Aliphatic *α,β*-unsaturated aldehydes are also readily reduced to their alcohols (**24e**–**g**), as is the furan analogue (**24h**). The notoriously difficult to hydrogenate substrate 2-thiophenecarboxaldehyde (**23i**), a known poison to homogeneous catalysts, is even converted to the corresponding alcohol (**24i**), and this shows the versatility of this catalyst system. The mild reaction conditions and the absence of base invoke that the supramolecular anionic bidentate ligand is involved in ligand-assisted hydrogen splitting.

##### 3.1.1.3 Hydrogen Transfer Reactions

**Scheme 11:**
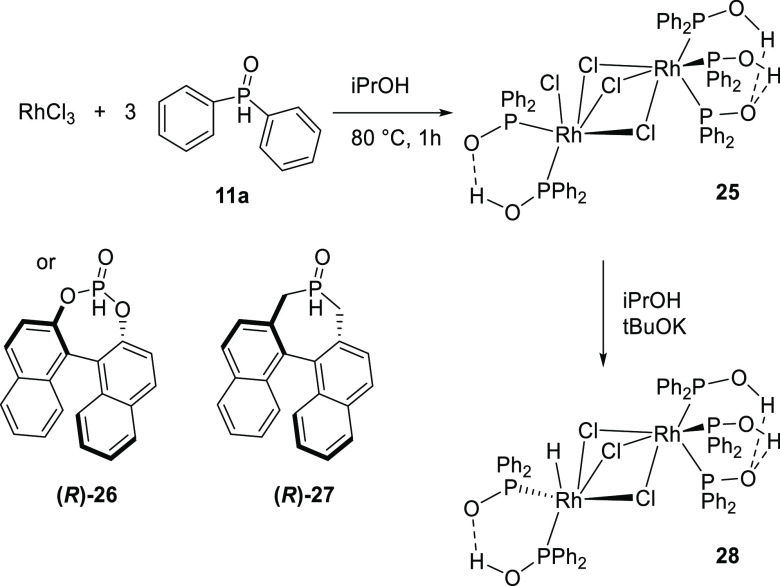
Formation of a Neutral Dimeric Complex by Reaction of Diphenyl Phosphine Oxide (**11a**) with RhCl_3_ and Subsequent Generation of the Rhodium-Hydride Species

Van Leeuwen and co-workers reported the rhodium(III) catalyzed transfer hydrogenation of ketones in isopropanol using SPOs and HASPOs, and although this is not covered in general in this review, it is briefly mentioned here in the context of this class of ligands.^238^ Out of an initial catalyst screening using various metal salts and the diphenylphosphine oxide ligand, using RhCl_3_ resulted in the highest activity using cyclohexanone and benzophenone as benchmark substrates (Scheme 11). Under optimal conditions, cyclohexanone was reduced with a 92% conversion and a TOF of 1825 mol_substrate_/mol_catalyst_·h^–1^. It turned out that the Rh/SPO ratio is crucial for good catalytic activity and was therefore optimized for every substrate. Spectroscopic studies confirmed that the neutral dinuclear complex (**25**) was formed, which interestingly bears a supramolecular bidentate ligand on one rhodium center and a supramolecular tridentate ligand on the other. The corresponding hydride complex (**28**) can be obtained by a reaction with butoxide as base. The asymmetric transfer hydrogenation of acetophenone was also studied using the chiral HASPO ligands **26** and **27**, yielding the product with an enantiomeric excess of 89%.

##### 3.1.1.4 Nitrile Hydration

**Scheme 12:**
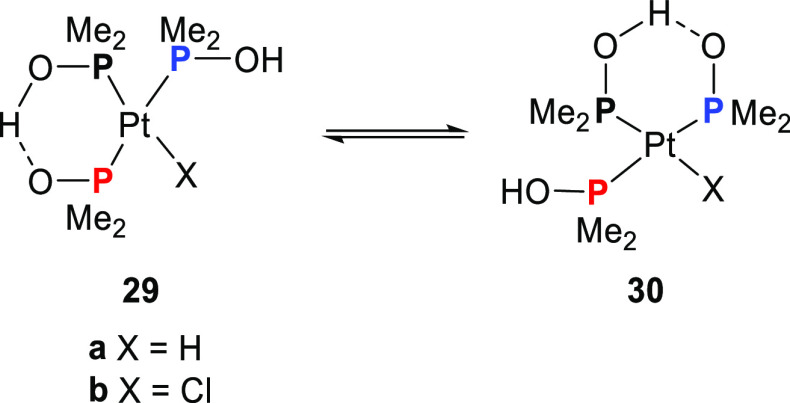
Fast Exchange between the Supramolecular Hydrogen-Bonded Bidentate and Monodentate Phosphinous Acid Ligands in Complexes Such as **29a** (X = H) and **29b** (X = Cl; See Also Scheme 13 for **29c** with X = OH)

The selective hydration of nitriles is an atom-economical and green way to produce *N*-unsubstituted amides which represent an important class of compounds. Parkins and Ghaffar showed that the platinum-hydride complex of dimethylphosphine oxide [PtH(PMe_2_OH){(PMe_2_O)_2_H}] (**29a**) and the corresponding chloride complex [PtCl(PMe_2_OH){(PMe_2_O)_2_H}] (**29b**) are active catalysts for the hydration of nitriles.^256−258^ As is illustrated in Scheme 12, in complexes such as **29** and **30** two SPO ligands are coordinated as hydrogen-bonded bidentate and one ligand is coordinated in a monodentate fashion which are in fast exchange at room temperature on the NMR spectroscopy time scale.

**Scheme 13:**
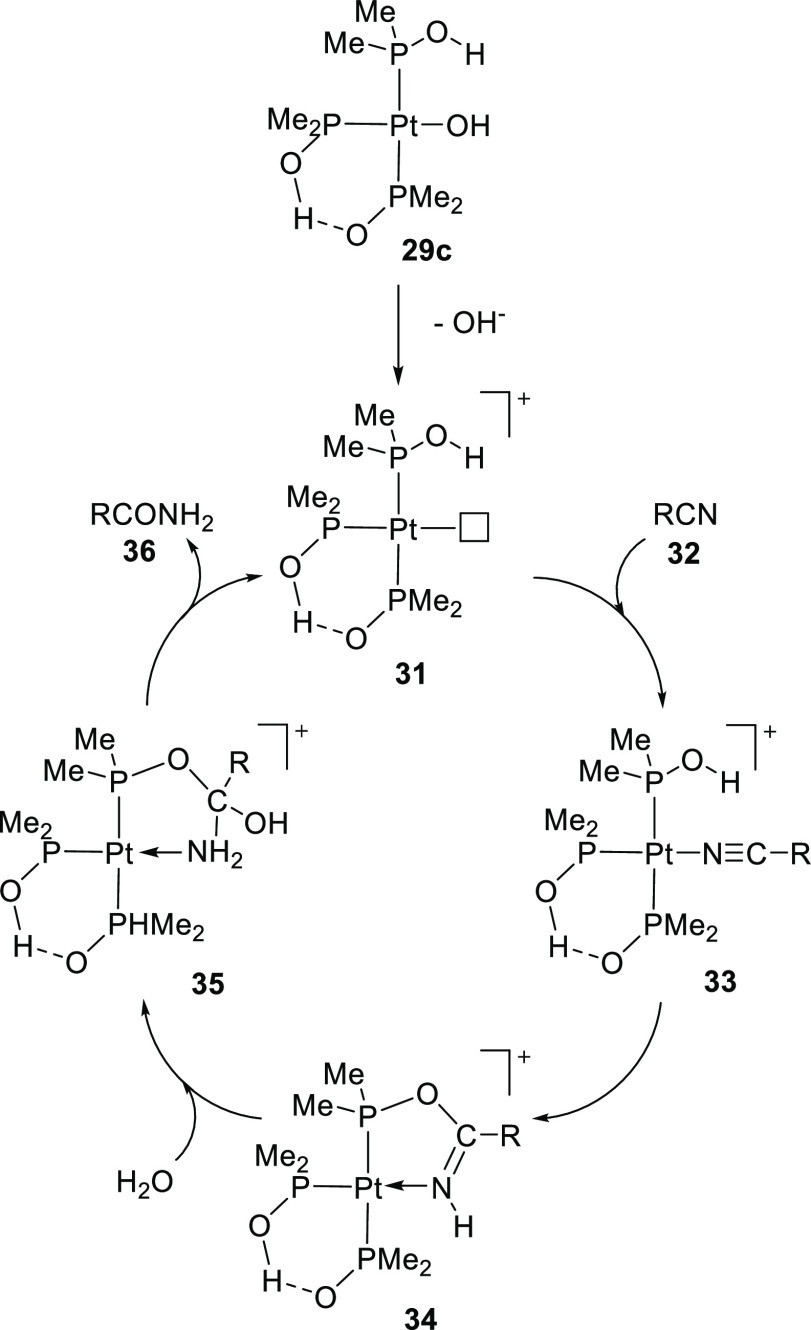
Hydration of Nitriles as Proposed by Parkins^256,259^

A plausible mechanism of nitrile hydration is given in Scheme 13 starting from **29c** where X = OH. The mechanism involves the initial coordination of the nitrile moiety (**32**) to the cationic platinum center (**31**), after which the nitrile is susceptible to nucleophilic attack.^256,259^ A key step in the suggested mechanism is the intramolecular nucleophilic attack of the hydroxy group of the coordinated phosphinous acid on the coordinated nitrile to give a five-membered ring (**34**). After attack of a water molecule, the *N*-unsubstituted amide is liberated and the cationic Pt species reforms, which completes the catalytic cycle. The size of the substituents on phosphorus has a large effect on the catalytic activity, and the highest activity was obtained with the ligand with the least sterically hindered groups studied, i.e, dimethyl phosphine oxide.

**Scheme 14:**
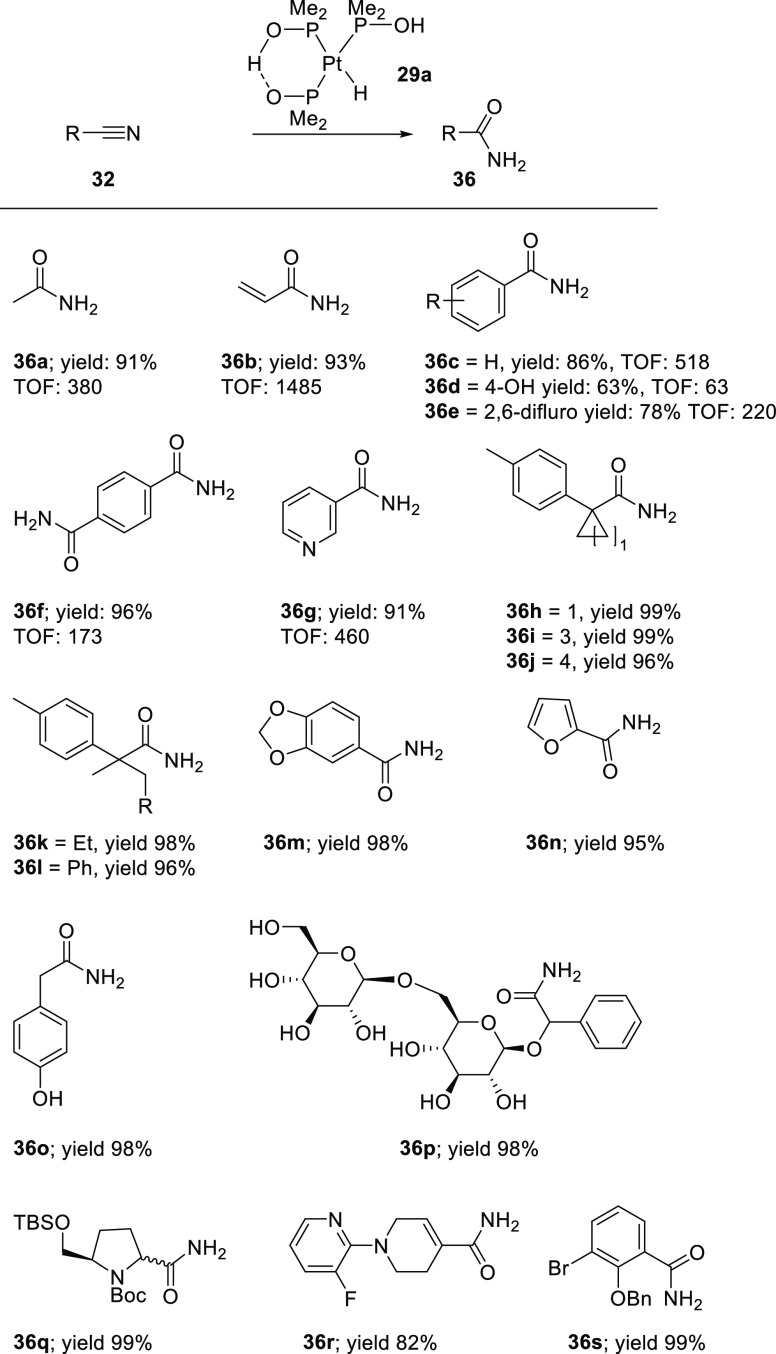
Substrate Scope of the Nitrile Hydration by the [PtH(PMe_2_OH){(PMe_2_O)_2_H}] Catalyst (**29a**)^256−258,260−263^

As is shown in Scheme 14, the [PtH(PMe_2_OH){(PMe_2_O)_2_H}] catalyst (**29a**) selectively hydrates different nitrile substrates in water, aqueous ethanol, or aqueous THF at 70–90 °C, yielding the amides as the only product in high yield with no further hydrolysis of the corresponding acids. Interestingly, nitrile hydration of acrylonitrile by **29a** proceeds smoothly with perfect chemoselectivity, leaving the carbon–carbon double bond untouched. The reaction only required 0.0013% catalyst, giving an impressive turnover number (TON) of 77.000. Also, sterically hindered tertiary nitriles and nitriles containing acid- or base-sensitive functional groups can be converted with excellent yields and chemoselectivities (see Scheme 14).^260,261^ Even the sensitive d-amygdalin (**36p**) was converted to the amide in 98% yield without racemization of any of the stereogenic centers in the sugar moieties.

Due to the exceptionally high functional group compatibility combined with the high activity, nitrile hydration has been a catalyst of choice in the synthesis of a large number of natural and biologically active products of elaborate structure.^259^ From the many examples reported in the open and patent literature summarized in Table 2, it is clear that the catalyst can tolerate a multitude of protecting groups but also tolerates strained ring systems (cyclopropyl) and activated C=C double bonds prone to Wacker oxidation and that even oxiranes are tolerated. Interestingly, the synthesis of 8-azabicyclo[3.2.1]octyl-2-hydroxybenzamide (**57**) includes a rare example of the hydration of thiocyanate (entry 17 in Table 2).

**Table 2:**
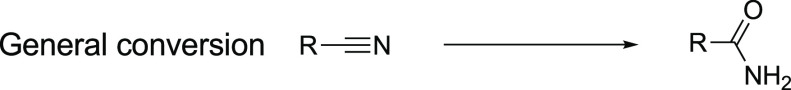

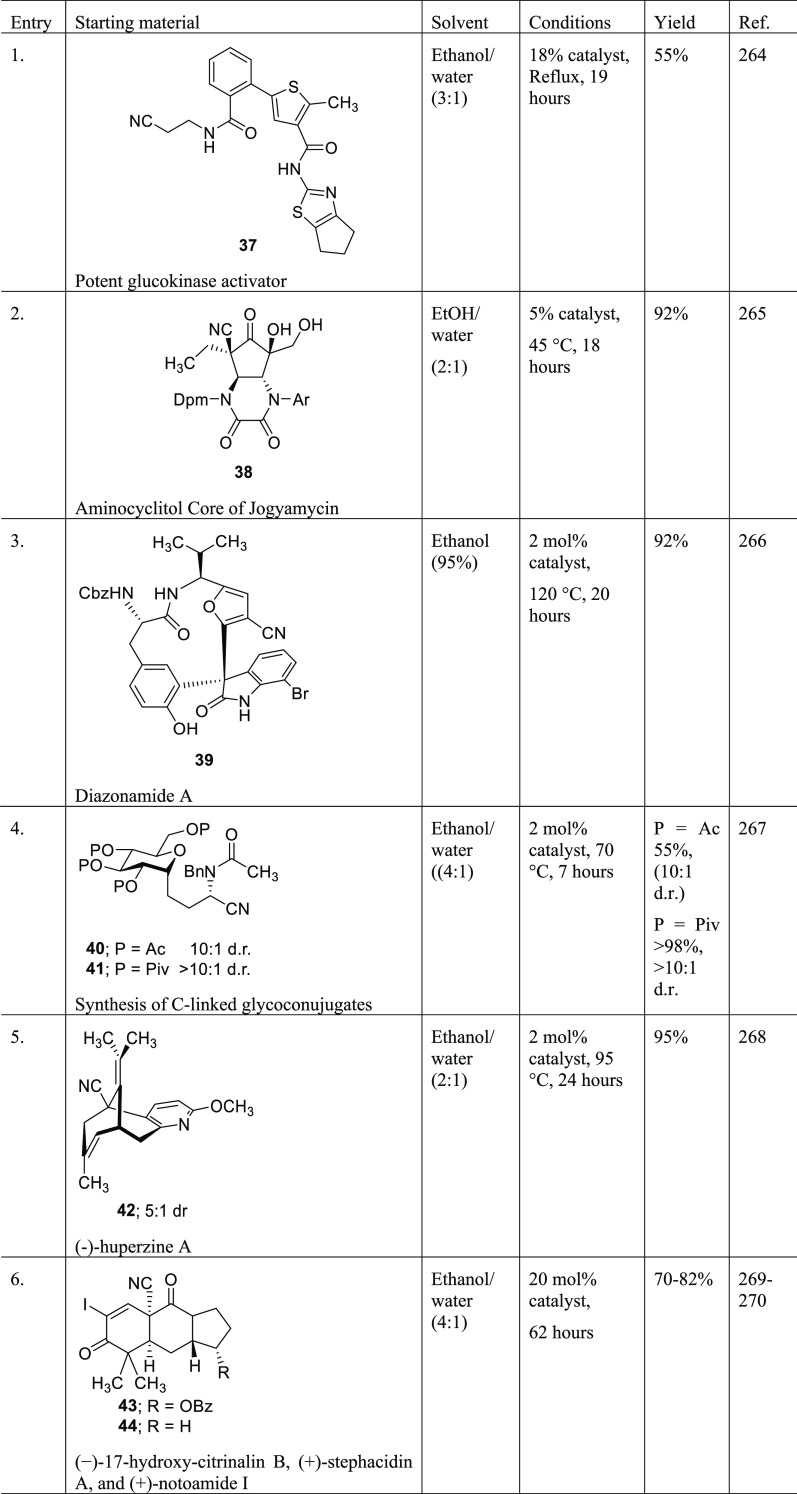

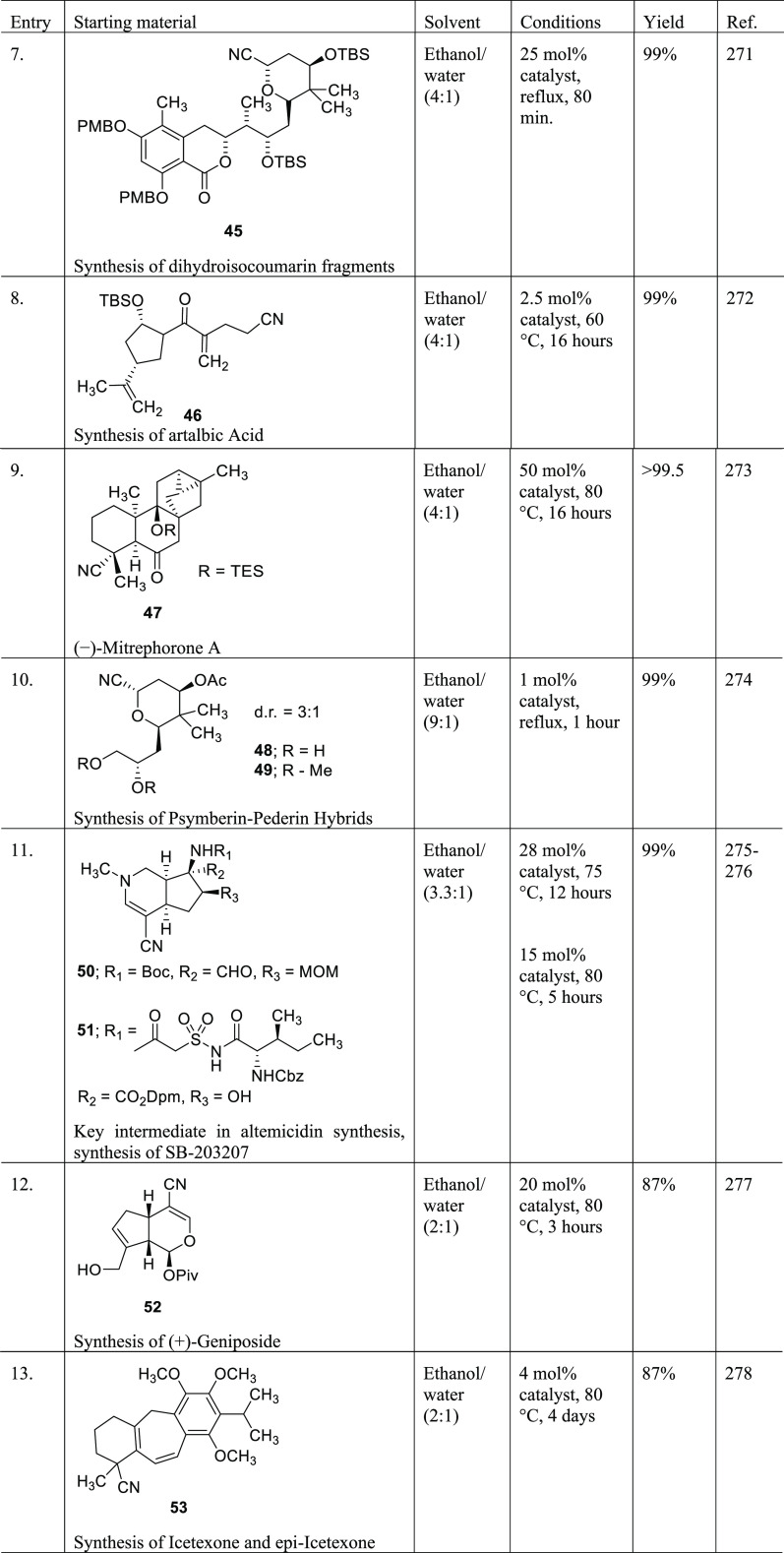

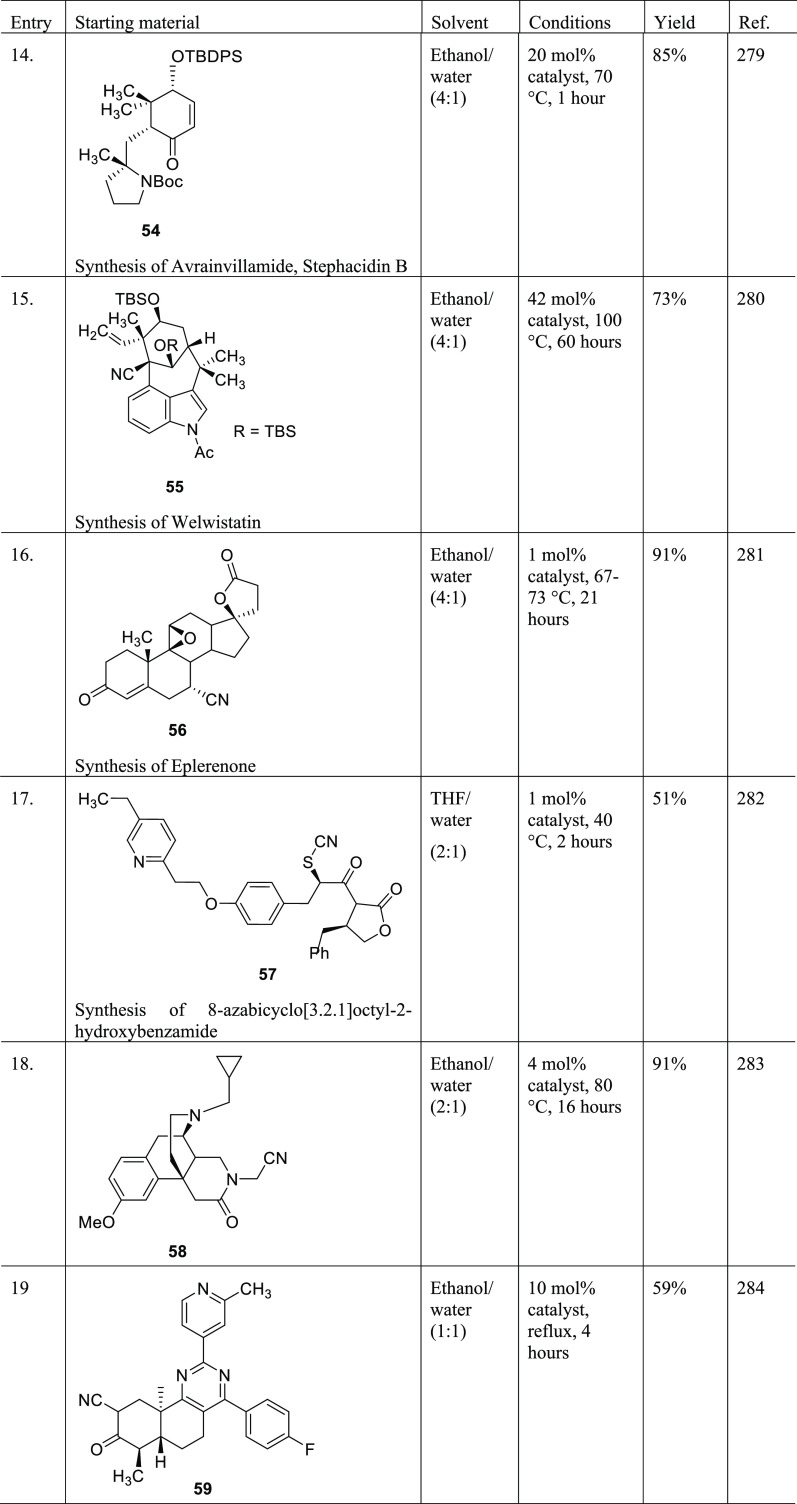
Overview of Natural Product Synthesis Using Parkins’ Hydration Catalyst [PtH(PMe_2_OH){(PMe_2_O)_2_H}] (**29a**)

**Scheme 15:**
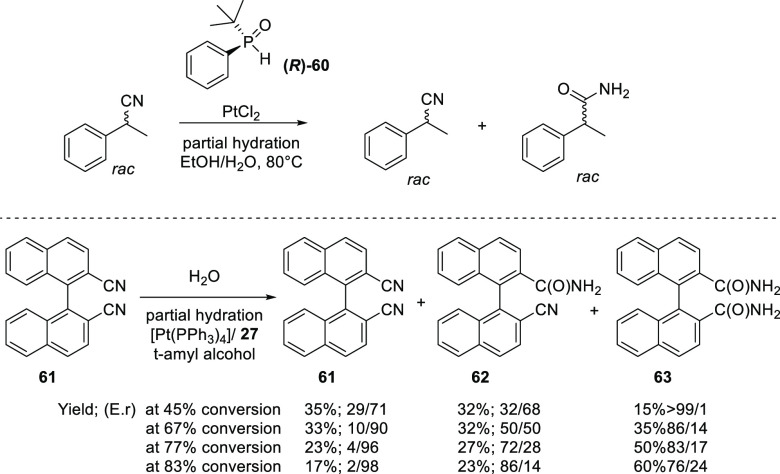
Attempted Kinetic Resolution in the Hydration of 2-Phenyl Proprionitrile Using PtCl_2_/**60** (Top) and in the Hydration of 1,1′-Binaphthalene-2,2′-dicarbonitrile (**61**) Using Pt(PPh_3_)_4_/**27** (Bottom)

Attempts have been made for the nitrile hydration in a kinetic resolution of 2-phenyl proprionitrile using (*R*)-(+)-(*t*-Bu)PhP(H)O (**60**) as chiral ligand (see Scheme 15, top).^261^ Analysis of the reaction shows that only the racemic product is formed, as the chiral SPO-ligand (**60**) was racemized under the reaction conditions. Interestingly, van Leeuwen showcased the possibility of kinetic resolution in the hydration of the racemic 1,1′-binaphthalene-2,2′-dicarbonitrile (**61**) in *tert*-amyl alcohol, illustrated in Scheme 15, bottom.^285^ The cationic platinum catalyst [Pt(PR_2_OH){(PR_2_O)_2_H}(solvent)] could be prepared *in situ* from either Pt(PPh_3_)_4_/(**27**) or Pt(COD)Cl_2_/AgNO_3_/(**27**) (for ligand **27** see also Scheme 11). In the hydrolysis reaction of **61**, only three compounds were observed in the reaction mixtures: enantioenriched dinitrile **61**, mononitrile-monoamide intermediate **62**, and the diamide **63**. With the Pt(PPh_3_)_4_/**27** catalyst at 83% conversion, the enantiomeric ratios for the three products are 2/98, 86/14, and 76/24, respectively.

Another interesting class of nitrile substrates to hydrolyze is the cyanohydrins, as it affords an atom-economical route to high value α-hydroxyamides, α-hydroxycarboxylic acids, and α-hydroxycarboxylic esters. An acid-free catalytic process is desirable as it reduces the number of side reactions and eliminates the stoichiometric formation of salts or alkyl chlorides.

**Scheme 16:**
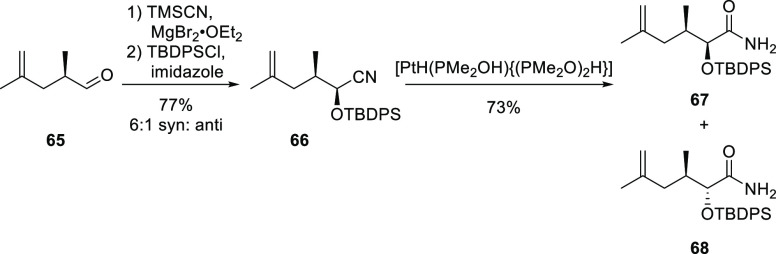
Syn-Selective Cyanide Induction of **65** to Produce the Unstable Cyanohydrin Which Was Immediately Protected by TBDPSCl (tert-Butyl(chloro)diphenylsilane) to Give **66**, Which Was Subjected to Nitrile Hydrolysis Producing **67** and **68**

Although the overall reactivity of [PtCl(PMe_2_OH){(PMe_2_O)_2_H}] (**29b**) in the hydrolysis of cyanohydrins is low, **29b** outperforms previously reported nitrile hydration catalyst Cp_2_Mo(OH)(OH)_2_ (**64**).^286^ The low reactivity of cyanohydrins is due to the liberation of hydrogen cyanide (HCN) by an undesired background reaction, which commonly leads to catalyst deactivation (cyanide coordination). As illustrated in Scheme 16, Brown and Konopelski bypassed this issue in their route to the total synthesis of Psymberin/Irciniastatin A by immediately protecting a cyanohydrin derived from **65** with a *tert*-butyldiphenylsilyl group in **66**.^287^ Subsequent nitrile hydrolysis of **66** using Parkins’ catalyst gave the separable diastereoisomeric amides **67** and **68** in 73% yields.

**Scheme 17:**
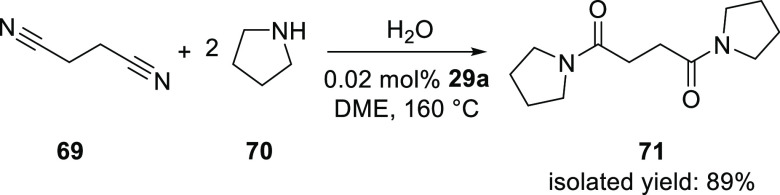
Direct Conversion of an Unactivated Dinitrile to Provide *N*-Substituted Amides Using **29a** (See Scheme 12)

The nitrile hydration has also been extended to one-pot sequential reaction methodologies. In this light, de Vries and co-workers reported the direct conversion of a number of unactivated nitriles to *N*-substituted amides with both primary and secondary amines, as exemplified in Scheme 17.^288^ The reaction is initially very fast and can be performed with catalyst amounts as low as 0.02 mol %. However, the ammonia produced by a reaction of the primary unsubstituted amide product with the amine leads to an appreciable reduction of the reaction rate as the reaction progresses. Primary and secondary amines work equally well; albeit, more forcing conditions are required for high conversions. The reaction of succinonitrile with pyrrolidine and water in DME at 160 °C catalyzed by [PtH(PMe_2_OH)(PMe_2_O)_2_H] (**29a**) gave the corresponding substituted amide in 89% isolated yield after 24 h.

The next example of the sequential reaction methodology that involves the nitrile hydration is the facile formation of 1-alkoxyisoquinolines and (2H)-isoquinolones by an intramolecular 6-*endo-dig* cyclization of *o*-alkynylbenzonitriles catalyzed by [PtH(PMe_2_OH){(PMe_2_O)_2_H}] (**29a**).^289^ An overview of the scope of this reaction is provided in Table 3. Substrates bearing both electron donating and electron withdrawing substituents at the *para*-position with respect to the alkenylphenyl moiety (R_1_ in Table 3) gave the cyclized products with comparable yields (entries 2 (**72b**) and 3 (**72c**) of Table 3) whereas substituents in the *ortho*-position hampered the cyclization process. In these reactions both the 1-alkoxyisoquinolines (**73a**–**j**) and the isoquinolones (**74a**–**j**) were isolated and the former could be efficiently converted into the isoquinolones in a subsequent reaction with HBr in acetic acid. Finally, cyano pyridines (X or Y = nitrogen) can be converted under the used reaction conditions. This approach has also been applied for the synthesis of heterocyclic antiviral compounds patented by Hoffmann-La Roche.^290^

**Table 3:**
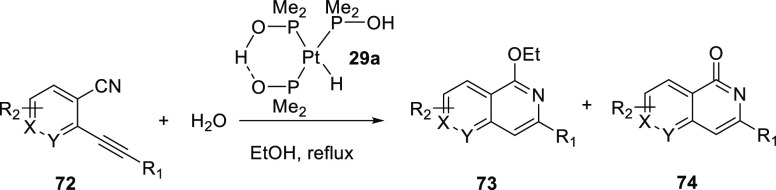
Scope of the Cyclization of *o*-Alkynylbenzonitriles (X and Y Can Be CH or N) and Amides Using **29a** (See Scheme 12)

Entry	Compound	R_1_	R_2_	Yield of **73a**–**l** (%)	Yield of **74a**–**l** (%)
1	**72a**	Ph	H	48	15
2	**72b**	*p*-MeO-C_6_H_4_	H	31	6
3	**72c**	*p*-F-C_6_H_4_	H	40	5
4	**72d**	*o*-CH_3_-C_6_H_4_	H	0	0
5	**72e**	*o*-Cl-C_6_H_4_	H	0	5
6	**72f**	3-Pyridyl	H	30	12
7	**72g**	3-Thiophene	H	49	14
8	**72h**	Cyclohexyl	H	43	19
9	**72i**	Ph	3-F	42	∼5
10	**72j**	Ph	5-CF_3_	37	11
11	**72k**	Ph	5-MeO	16	<5
12	**72l**	Ph	4-Me	40	16

##### 3.1.1.5 Oxidation

**Scheme 18:**
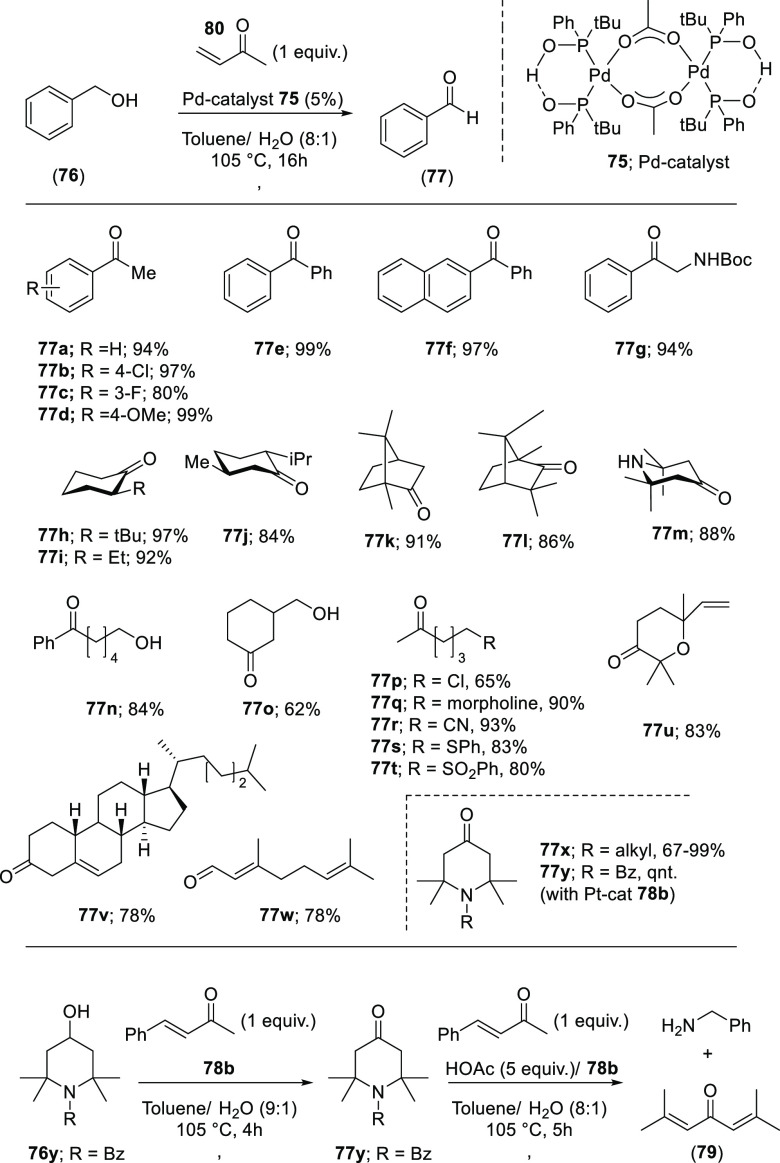
Scope of Pd Catalyzed (**75**) Alcohol Oxidation Reaction Showing Reaction Products Ketones **77x**–**y** were produced using a similar Pt catalyst ([Pt(OH){(P(*t*-Bu)(Ph)O)_2_H}] (**78a**).

Nuel, Giordano, and co-workers reported a Pd(II) catalyzed oxidation of alcohols using catalyst (**75**) with regeneration of the active Pd species through hydrogen transfer to an alkene (see Scheme 18).^291^ This so-called “hydrogen abstracting methodology” (HAM)^292^ is performed under relatively mild conditions at neutral pH and allows for the wide substrate scope displayed in Scheme 18. Benzylic alcohols **76a**–**g** and **76n**, aliphatic cyclic alcohols **76h–m, o, u,** and **v**, and acyclic secondary alcohols **76p**–**t**, including sterically congested substrates, are oxidized to the corresponding ketones. Interestingly, under the applied reaction conditions the nitrile group remained unaffected, which is evidenced by the conversion of **76r** to **77r** in 93% yield. Also, other strongly coordinated moieties did not hamper the oxidation, and the alcoholic substrates containing secondary sulfide, sulfoxide, and morpholine moieties are readily oxidized, showing the potential of the approach. Also, diols containing both primary and secondary alcohol functions were converted with high chemoselectivity; that is, the secondary alcohol was converted to the corresponding ketone while the primary alcohol remained untouched (**76o**).

**Scheme 19:**
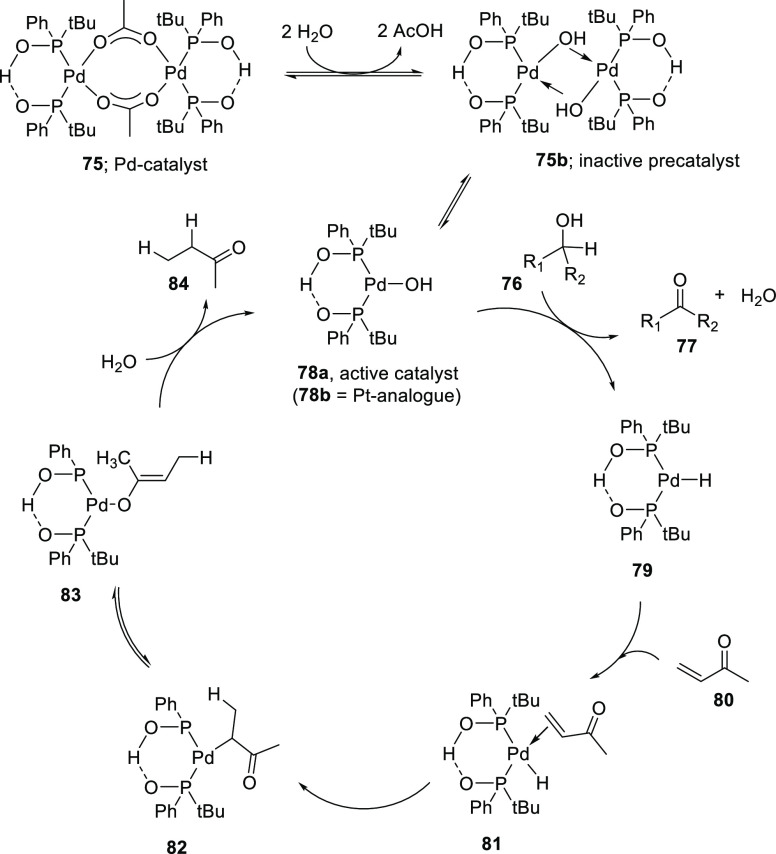
Proposed Mechanism of the Hydrogen Abstracting Methodology

After initial hydrolysis of complex **75**, the proposed mechanism starts with the catalytically active monomeric [Pd(OH)(P(*t*-Bu)PhO)_2_H] (**78a**), which reacts with the substrate alcohol, thereby producing the ketone and yielding the corresponding [Pd(H){(P(*t*-Bu)PhO)_2_H}] complex **79**. This complex then reacts with the hydrogen acceptor (methyl vinyl ketone) **80** in a stepwise 1,4-addition to produce methylethyl ketone **84** and concomitantly reform the [Pd(OH){(P(*t*-Bu)PhO)_2_H}] (**78a**) which closes the catalytic cycle (Scheme 19).

Similar reactivity has been found for platinum complexes. For example, *in situ* formed hydroxy-platinum [Pt(OH){(P(*t*-Bu)(Ph)O)_2_H}] catalyst **78b** was found to be a superior catalyst for the aerobic/anaerobic oxidation of challenging substrates such as *N*-alkyl-2,2,6,6-tetramethylpiperidin-4-ols (**76x**) and analogues, which are smoothly oxidized to the corresponding ketone (**77**) at room temperature in good to excellent yields (67–99%).^239^ The corresponding palladium complexes were not active under the applied reaction conditions. This study was further extended to the oxidative defragmentation of *N*-alkyl-2,2,6,6-tetramethylpiperidin-4-ols (**76x**), which could be accomplished in a two-step one-pot process.^293^ The [Pt(OH){(P(*t*-Bu)(Ph)O)_2_H}] catalyst (**78b**) plays a dual role, and the supramolecular hydrogen-bonded ligand acts as a hydrogen source and the cationic metal center as Lewis acidic site. Under optimized reaction conditions the substrate *N*-benzyl-2,2,6,6-tetramethylpiperidin-4-ol (**76y**) was oxidized by the *in situ* formed [Pt(OH){(P(*t*-Bu)PhO)_2_H}] to the corresponding ketone *N*-benzyl-2,2,6,6-tetramethylpiperidin-4-one (**77y**) under basic conditions within 4 h at 105 °C. Subsequent addition of 5 equiv of acetic acid and hydrogen acceptor *trans*-phenylbut-3-en-2-one followed by another 5 h of reaction time lead to the fragmentation of the ketone **77y** to obtain phorone 2,6-dimethylhepta-2,5-dien-4-one (**79**) and benzylamine. The goal of the authors was to isolate the liberated alkyl amine, which was isolated conveniently by a simple acid/base extraction in 86% yield.

With this Pt catalyst system (**78b**), also challenging substrates such as 1,2- or 1,3-diols can be converted into α- or β-hydroxy ketones in moderate to good yields (44–89%). The relatively mild reaction conditions also allow the oxidation of substrates containing base-sensitive functional groups such as esters. For example, ethyl 3-hydroxycyclohexane-1-carboxylate **(85)** is readily oxidized to the corresponding ketone.^239^ The platinum-SPO catalyzed oxidation proceeds via a proposed mechanism similar to the palladium catalyzed oxidation (*vide supra*).

##### 3.1.1.6 Cross-coupling

**Scheme 20:**
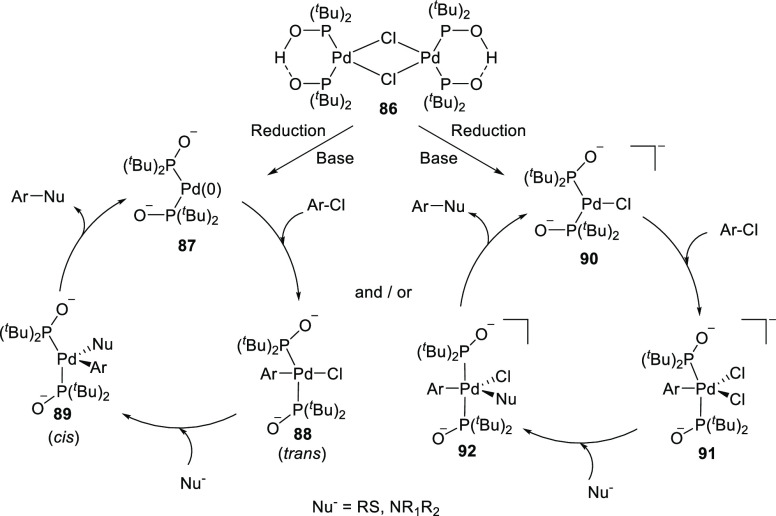
Proposed Mechanism for Pd-SPO (**86**) Cross-coupling Chemistry by Li et al.^294^

The palladium- and, to a lesser extent, the nickel catalyzed cross-coupling chemistry have been extensively studied using SPO ligands, and the literature has been reviewed previously.^236,294^ One of the earliest examples is provided by Li and co-workers,^294^ who demonstrated that the coupling of aryl chlorides and *tert*-butyl acrylate in the presence of K_2_CO_3_ in *N*,*N*-dimethylformamide (DMF) could be accomplished using 1.5 mol % of the palladium dimer catalyst (**86**), resulting in α,β-unsaturated esters in good yields. A proposed mechanism for this reaction is shown in Scheme 20. Further research has shown that this catalyst is active in a wide variety of C–C, C–N, and C–S bond forming processes involving aryl chlorides. The high activity toward the aryl chlorides is attributed to the cleavage of the palladium(II) dimer **86** and deprotonation of both phoshinous acid ligands, yielding an electron-rich phosphine-containing anionic complex (**87** or **90**), which accelerates the rate determining oxidative addition of aryl chlorides in the catalytic cycle (from **87**/**90** to form **88**/**89**). Although the results show the potential of SPO-containing catalysts for cross-coupling reactions, the supramolecular bidentate structure is not maintained during catalysis.

#### 3.1.2 Phosphine-Phosporamidite Single HB Systems

**Figure 3:**
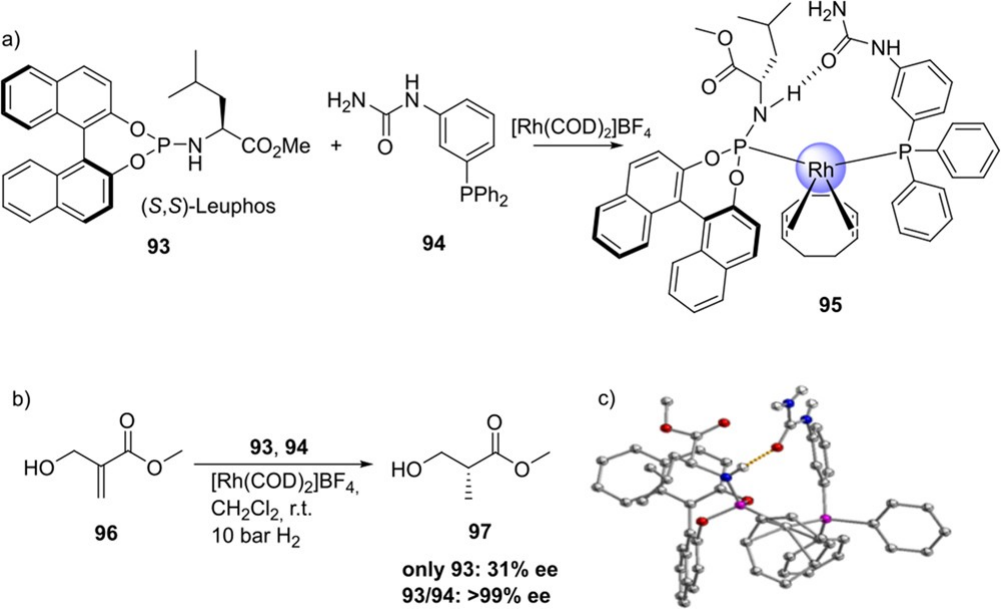
(a) Ligand building blocks that form single hydrogen-bonded bidentate ligands when coordinated to rhodium. (b) Rhodium catalyzed asymmetric hydrogenation of Roche ester precursor by supramolecular bidentate ligand. (c) HB is indicated by a dotted line in the X-ray structure.

Reek and co-workers reported in 2009 the formation of supramolecular heterobidentate ligands formed by a single HB.^295,296^ As is shown in Figure 3a, leucine-based phosphoramidite ligand **93** contains a strongly polarized N–H that forms a HB with the carbonyl group of the urea functionalized phosphine ligand (**94**) when coordinated to a rhodium center. The existence of the HB is also clear from the X-ray structure (see Figure 3c). This HB was also observed by IR spectroscopy in the [Rh]BF_4_ complex and confirmed by DFT calculations to be more stable than the N–H···O=C^urea^ HB in the absence of the metal. In the IR spectroscopic data, the IR band corresponding to the ester moiety remains unchanged whereas the band corresponding to the carbonyl of the urea shifts from 1703 to 1681 cm^–1^, indicating that the urea is involved in a HB as a proton acceptor. The phosphoramidite ligand building block (**93**) as well as the phosphine building block (**94**) can be varied by changing the electronic and steric properties, and various combinations were studied in the rhodium catalyzed hydrogenation of methyl 2-hydroxymethyl acrylate (**96**) (Roche ester precursor).^295^ Among the combinations, the supramolecular bidentate ligand based on (*S*,*S*)-Leuphos (**93**) and the monourea triphenylphosphine **94** gave the highest enantioselectivity (99%+ e.e.), while only (*S*,*S*)-Leuphos (**93**) as ligand showed only 31% e.e., as is shown in Figure 3b. Further detailed investigation of this catalytic system led to the observation that substrate orientation by hydrogen bonding between the functional group of the substrate and the ligands is important in achieving high selectivity, and this will be discussed in section 4.1.

### 3.2 Supramolecular Ligands Using Multiple HBs

**Scheme 21:**
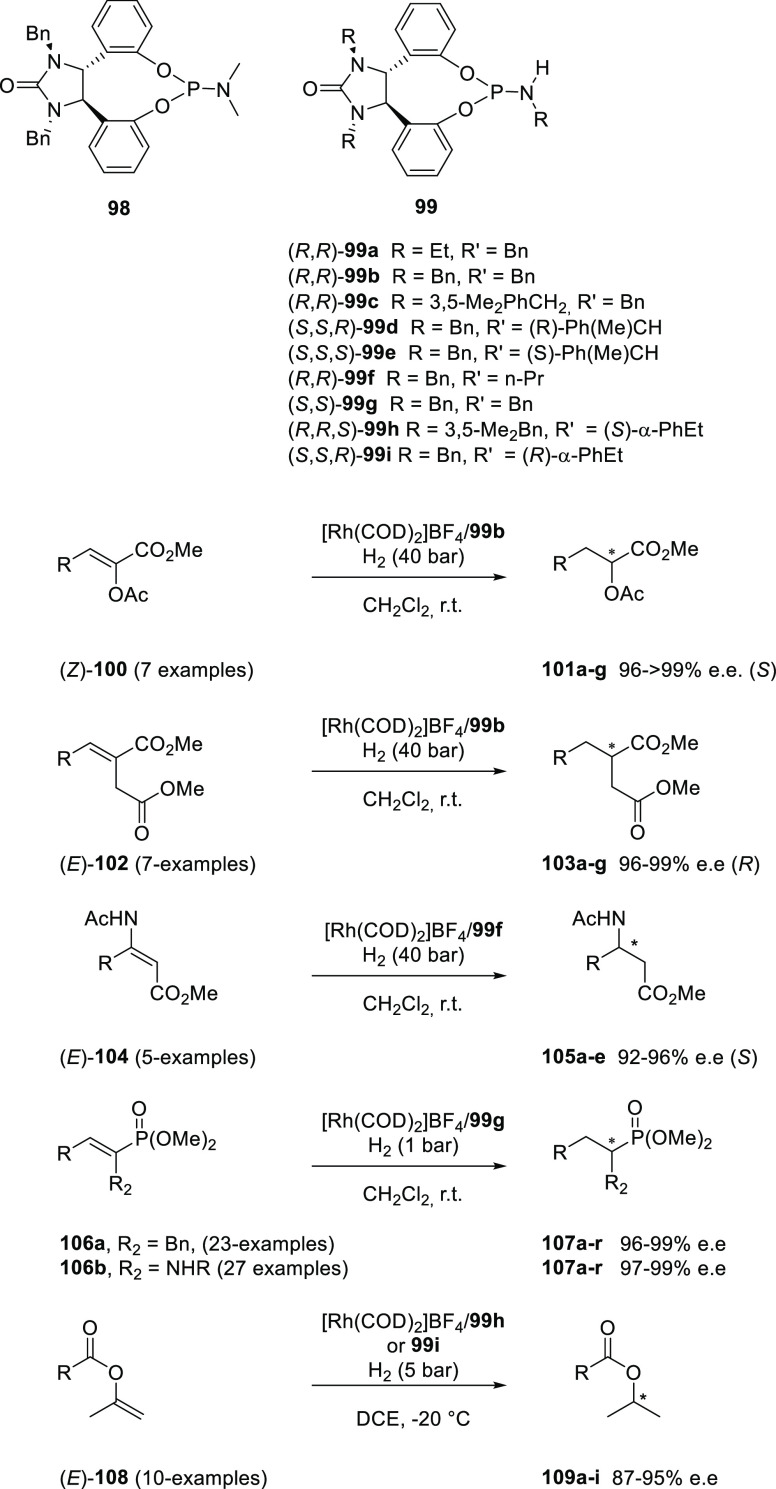
(*R*,*R*)-DPenPhos-Me (**98**) and (*R*,*R*)-DPenPhos-H (**99**) Ligands Developed by Ding et al. and Applied in the Asymmetric Hydrogenation of Different Substrate Classes

**Figure 4:**
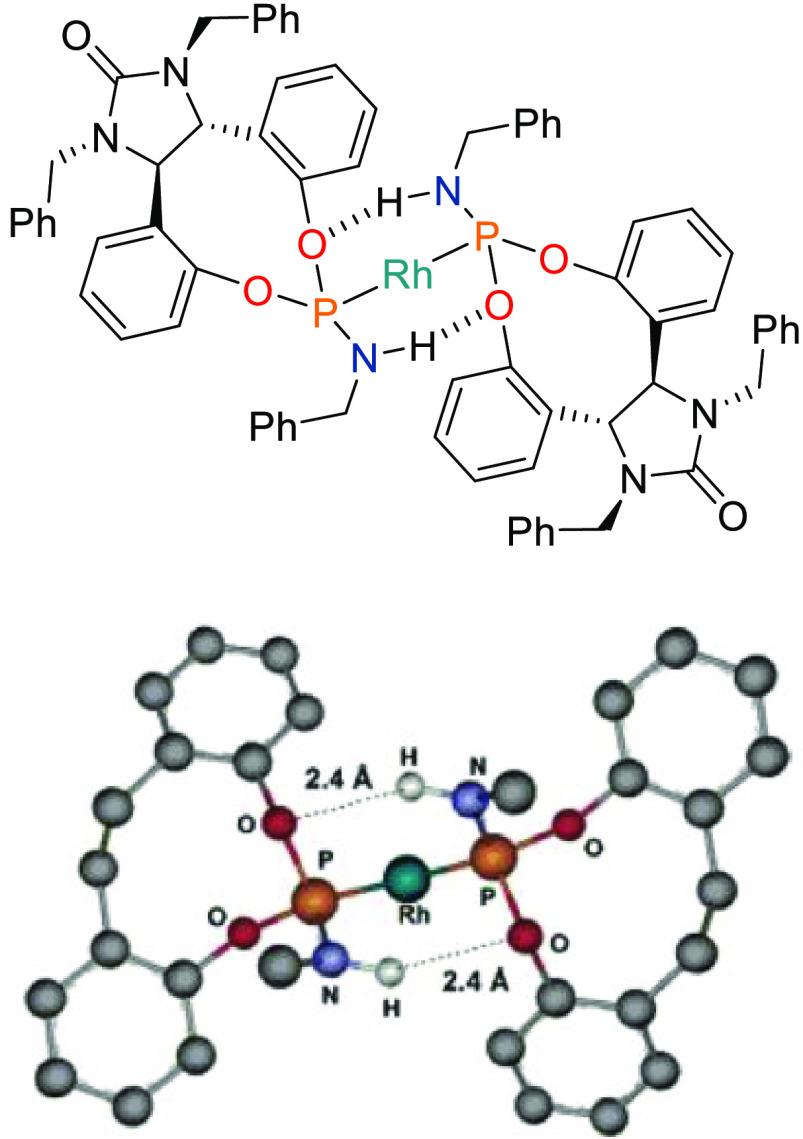
Structure of Rh[DPenPhos-H (**99b**)]_2_ (left-hand side) and its B3LYP/6-31G(d) optimized structure using a structural mimic (right-hand side). For both, the COD ligand is omitted for clarity. Adapted with permission from ref (297). Copyright 2006 American Chemical Society.

Ding and co-workers explored the use of phosphoramidite (DPenphos)-type ligands in the asymmetric hydrogenation of (*Z*)-methyl α-(acetoxy)acrylates and (*E*)-α-aryl itaconate derivatives containing various substituents.^297^ Catalysts based on (*R*,*R*)-DPenPhos-H (**99**) provided full conversion and excellent enantioselectivity (94–99%) (see Scheme 21). The enantiomeric excess induced by the Rh((*R*,*R*)-DPenPhos-H systems was not majorly affected by the substituent on the phosphoramidite nitrogen atom. DFT calculations and NMR spectroscopic studies confirmed the presence of N–H ···O HBs between two coordinated DPenphos ligands (see Figure 4 and Scheme 211) exhibiting the relatively small interligand bite angle of 89.9°. The proximity of the two ligands to the substrate as programmed by the HBs subtly influences catalyst structure and reactivity, and according to the authors, the hydrogen-bonded bidentate structure is maintained under the hydrogenation conditions (employing nonpolar solvents). Remarkably, the closely related (*R*,*R*)-DPenPhos-Me and Monophos did not display any reactivity under the applied reaction conditions; both are based on the dimethylamino-phosphoramidite and, thus, are not able to form the hydrogen bonding bidentate structure. Later, the substrate scope was further extended to the asymmetric hydrogenation of (*E*)- or (*Z*)-β-substituted dehydro-β-amino acid esters, and it was again reported that the N–H moiety in the phosphoramidite ligand is critically important for achieving high activity, and catalysts based on ligand **99f** provided 92–96% e.e.^298^ The versatility of the Rh/DPenphos-H catalyst system was further demonstrated by the asymmetric hydrogenation of α- or β-acyloxy *α,β*-unsaturated phosphonates (**100**) and α- and β-enamido phosphonates (**101**).^299−301^ During these studies, it surfaced that the ligand (*R*,*R*)-DPenPhos-H (**99**) provided superior enantioselectivities (96–99+% e.e.) for a large substrate scope consisting of 50 entries and asymmetric hydrogenation of enol esters (**100**, **102**, **104**, and **108**) with enantioselectivities between 87 and 95% e.e. Besides benchmark reactions, Rh/(**99b**) has also been successfully applied in the synthesis of biologically active compounds such as the Danshensu–cysteine conjugate, which has considerable interest due to its various biological activities, such as antioxidative compounds and daidzein derivatives.^302,303^

**Scheme 22:**
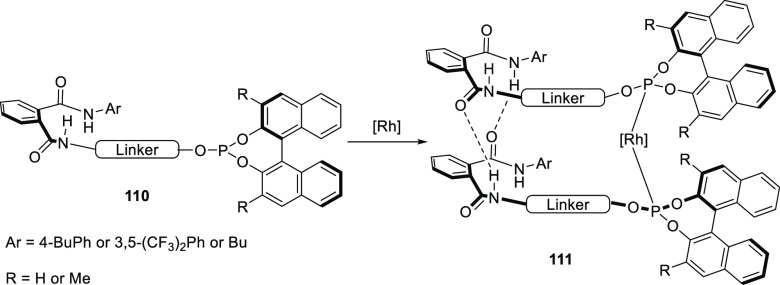
General Design of Phthalaphos Ligands (**110**) That Form Supramolecular Bidentate Ligands by Hydrogen Bonding When Coordinated to a Metal (e.g., Rh)

In the 2010s, Gennari and co-workers reported Phthalaphos ligands, which are binol-derived phosphites linked to a phthalic amide moiety (**110**); see Scheme 22.^304,305^ These ligands form a supramolecular bidentate complex based on HBs between two phthalic amides, as is shown on the right-hand side of Scheme 22. The authors investigated the hydrogen bonding of a Phthalaphos rhodium precatalyst by ^1^H NMR and IR spectroscopy and confirmed the supramolecular bidentate behavior in the precatalyst complex. A library of these ligands consisting of 19 ligands was studied in the rhodium catalyzed hydrogenation of several prochiral dehydroamino esters and enamides, yielding excellent e.e.’s of up to 99% for the benchmark substrates (e.g., methyl 2-acetamido acrylate, methyl (*Z*)-2-acetamido cinnamate, and *N*-(1-phenylvinyl)acetamide). Also, challenging and industrially relevant substrates such as *N*-(3,4-dihydronaphthalen-1-yl)-acetamide and methyl (*E*)-2-(acetamidomethyl)-3-phenyl acrylate could be converted with 96% and 99% e.e., respectively. DFT calculations suggest that the phthalic acid amide is also involved in the substrate preorganization, at the expense of the supramolecular bidentate ligand, and is responsible for the observed high selectivities. The substrate preorganization will be discussed in more detail in section 4.

**Scheme 23:**
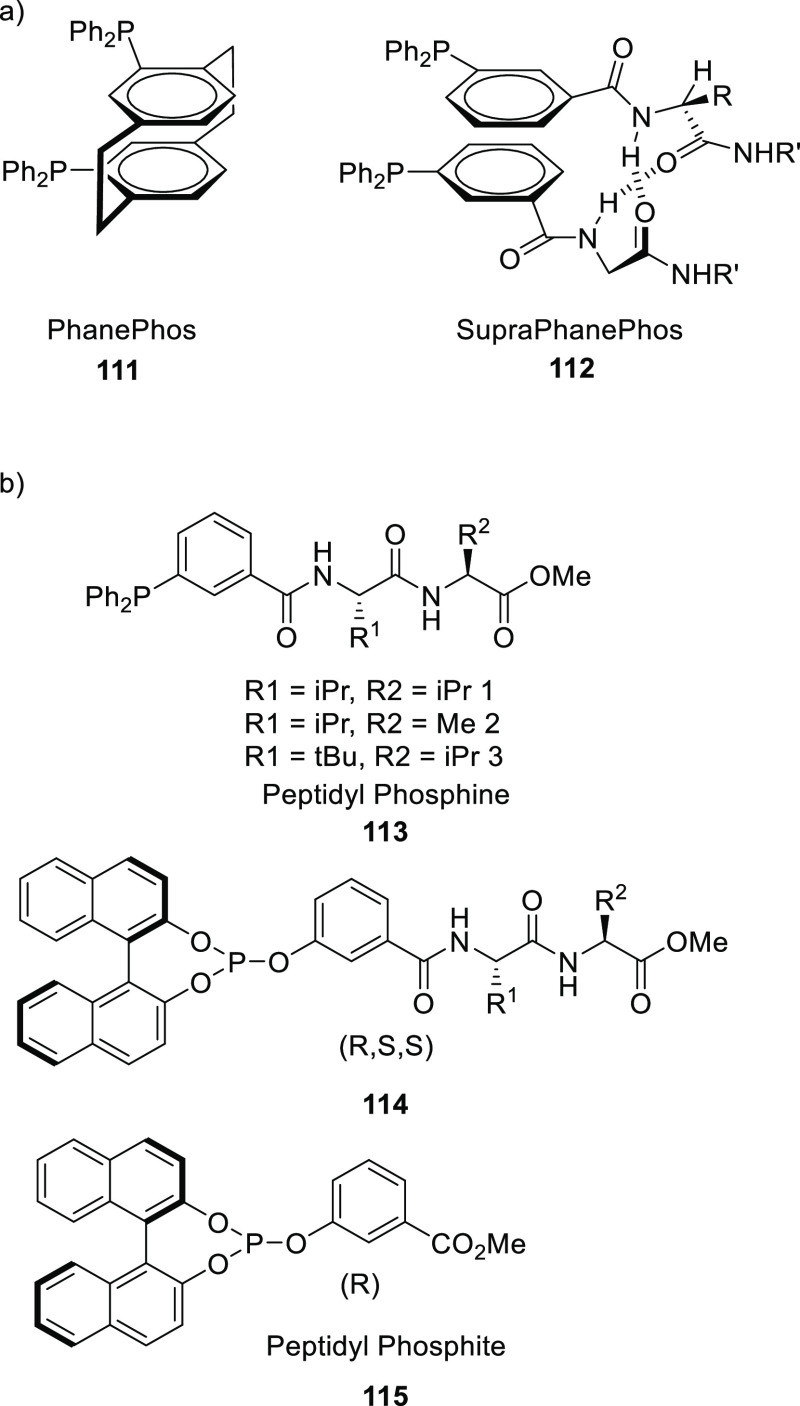
(a) Concept of the SupraPhanePhos (**112**). (b) Different Peptidyl Phosphine and Phosphite Ligand Building Blocks Used in the Rhodium Catalyzed Asymmetric Hydrogenation

Another explored strategy using amides as HB motif was developed by Breit and co-workers in 2008.^306^ As is illustrated in Scheme 23, “SupraPhanePhos” (**112**) is a motif structurally similar to regular “PhanePhos” (**111**) (a well-known covalently linked bidentate ligand) that is held together by HBs with its peptidic fragment. X-ray spectroscopic analysis of a complex formed after SupraPhanePhos coordination to PtCl_2_ revealed a helical conformation of the hydrogen-bonded peptidyl chains. ^1^H NMR spectroscopic studies further confirm that this conformation remained intact in solution. Screening of binary mixtures of SupraPhanePhos (**112**) ligands in the rhodium catalyzed asymmetric hydrogenation of a number of benchmark substrates (e.g., methyl *N*-acetyl dehydro-amino acids and dimethyl itaconate) showed that the peptidyl phosphite-based complexes gave a selectivity of up to 99%, which is higher than that obtained using phosphine analogues. The enantioselectivities are moderate (up to 51%), yet these results are interesting because the chiral centra in SupraPhanePhos complexes are at least seven atoms away from the active metal site. The authors observed a match/mismatch effect by changing the binol enantiomer in combination with the chiral peptide. Also, for one of the reactions the complex based on the ligand without any peptidyl chain gave the highest e.e., suggesting a negative effect of the supramolecular chelate ligand.

**Scheme 24:**
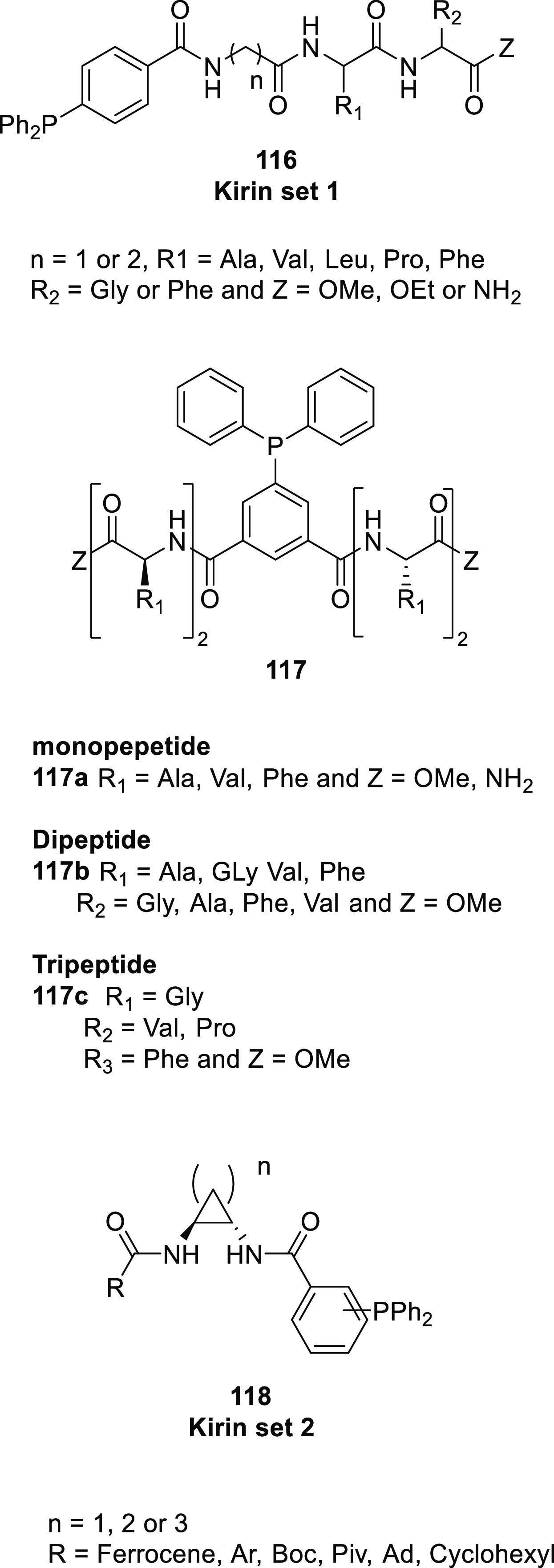
Chirality Induction by Distant Chiral (Peptidyl) Groups as Developed by Kirin and Coworkers

The chirality transfer between the distant chiral peptidyl chain and the metal center, also referred to as “*backdoor induction*” but perhaps better described by the “*chirality relay mechanism*”, was further developed by Kirin and co-workers using a slightly modified SupraPhanePhos ligand design. Most notably, in their approach, Kirin and co-workers installed the phosphine group *para* with respect to the peptidyl chain. This ensures that the ligand is relatively insensitive to rotation around the phenyl–peptidic C–C bond. Another feature of Kirin’s design is that the peptidyl chains are terminated by an amide group for a more extended hydrogen bonding array (Kirin set 1, see Scheme 24).^307^ Asymmetric hydrogenation of methyl 2-acetamidoacrylate using Rh complexes of their ligands resulted in moderate e.e.’s, which were nonetheless an improvement over an analogous complex reported by the Breit group (from 51% to 68% e.e.). The strategy was extended to bispetidyl phosphine ligands with amino acids (**117a**) and dipeptidyl (**117b**) and tripeptidyl chains (**117c**). After complexation to [Rh(COD)_2_]BF_4_, these ligands form intermolecular hydrogen-bonded β- or γ-turns.^308^ Both unary and binary mixtures were studied in the Rh catalyzed asymmetric hydrogenation of a number of benchmark substrates yielding up to 80% e.e.

Designs using a more rigid backbone based on cyclic diamine (Kirin set 2; see Scheme 24) provided catalysts that induced higher e.e. in the conversion of the same substrate classes (up to 92% e.e.) and up to 97% e.e. in the case of the phenyl derivative.^309^ Characterization of the hydrogen bonding of the ligand building block by NMR and IR spectroscopy indicated that only one amide N–H was involved in the interligand hydrogen bonding and one amide proton is not hydrogen bonded. For the square planar Pt and Rh complexes, circular dichroism showed a metal–ligand absorption around 435 nm corroborating the relay of chirality to the metal complex.

#### 3.2.1 Urea-Based Supramolecular Bidentate Ligands

**Figure 5:**
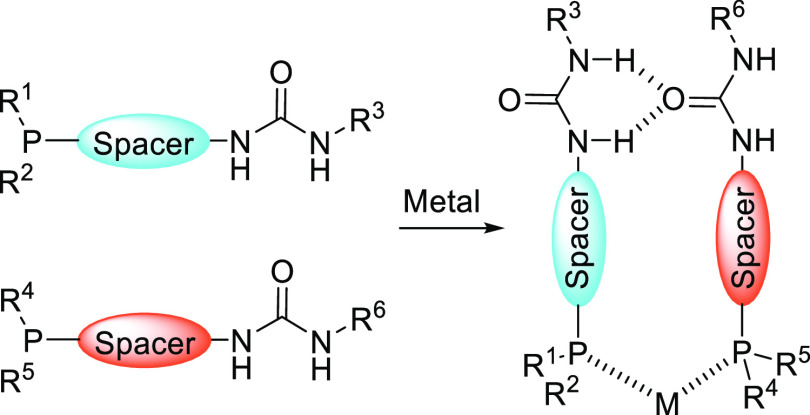
Self-assembly of UREAphos ligand systems to form supramolecular bidentate ligands in the presence of palladium or rhodium precursors (M).

Reek and co-workers reported supramolecular bidentate P-donor ligands based on ureas as a self-complementary hydrogen bonding motif. The targeted synthons to build such ligands are selected to be either commercially available or easily prepared. This makes the approach modular, and with it, it is easy to build a large and diverse ligand library that easily exceeds 100,000 members. These “UREAPhos” ligands are easily accessible by simple “click”-type reactions allowing parallel automated synthesis of 32 new ligand building blocks in a single day, highlighting the potential of this approach.^310^ Assembly of two of such phosphine ligand building blocks allowed the formation of hydrogen-bonded chelating structures in the presence of Pd and Rh complexes, as illustrated in Figure 5 and evidenced by detailed NMR spectroscopic studies.^311^

**Scheme 25:**
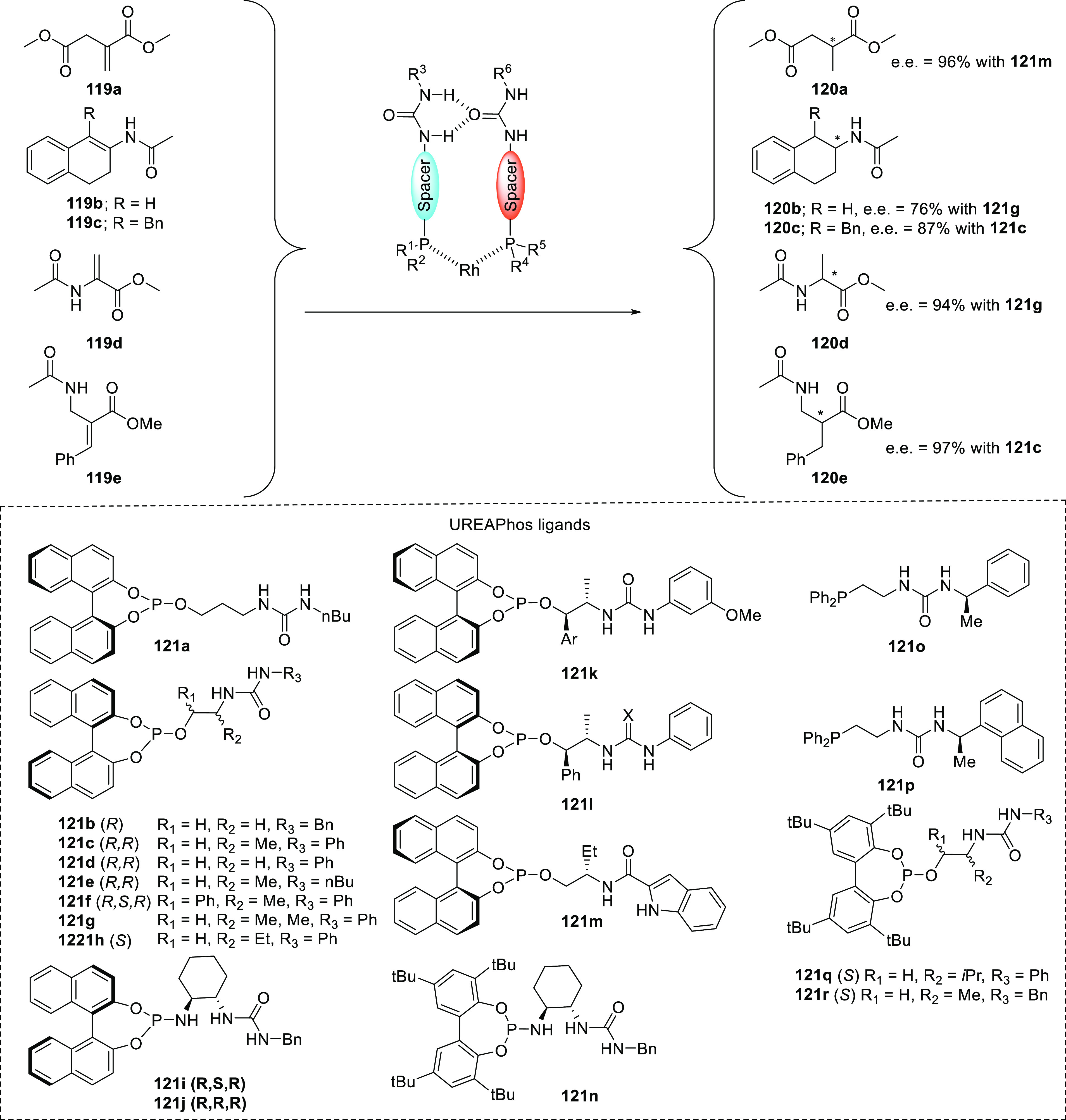
Asymmetric Hydrogenation Reaction of a Series of Substrates (**119**) to Useful Products (**120**) Using a Library of Rhodium Catalysts Based on Self-Assembled Bidentate UREAPhos Ligands (**121**)

In two separate studies the use of a significant library of 18 structurally related ligands (summarized in Scheme 25) was explored in the asymmetric hydrogenation of industrially relevant prochiral substrates, revealing that the products were formed in high selectivities.^310,312−314^ Importantly, small changes in the spacer unit between the donor atom and the urea motif resulted in large variation in the catalytic performance of the related rhodium complex, making variation in the UREAphos structure relevant. The observed enantioselectivities are good to high, also for the inherently difficult substrates (**119e**) and the tetrasubstituted substrate (**119c**), which were hydrogenated to form the product in 97% and 87% e.e., respectively. For **119e**, a substrate–ligand interaction has been invoked based on NMR and IR spectroscopic studies.^314^ These results support the significance of generating large and diverse catalyst libraries for lead discovery. After optimization of the reaction conditions for two successful catalysts, it was demonstrated that the catalyst activity is easily improved by varying the temperature. As the UREAPhos ligands are prepared by simple reactions steps, scale-up of the catalysts is relatively easy.

**Scheme 26:**
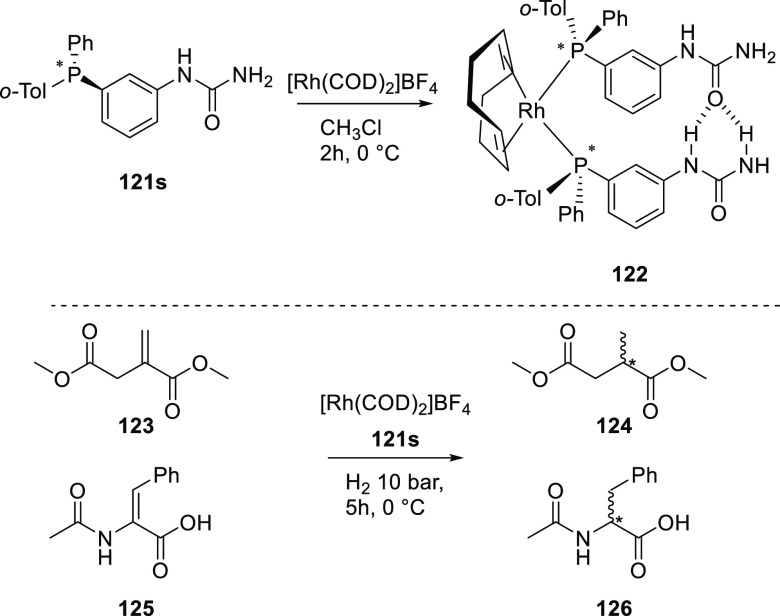
P-Chiral UREAphos and the Application in the Rhodium Catalyzed Hydrogenation of Dimethylitaconate and *N*-Acetyldehydrophenylalanine

Chikkali and co-workers further extended the approach to P-chiral UREAPhos ligand building blocks to form complex **122** shown in Scheme 26.^315^ Formation of **122** upon coordination with [Rh(COD)_2_]BF_4_ was confirmed by ^1^H NMR and IR spectroscopy, and upon application in the hydrogenation of dimethylitaconate, the complex gave only 33% e.e. whereas *N*-acetyldehydrophenylalanine was converted with 99% e.e.. Further investigations suggest that with this substrate’s HBs between the substrate and the urea moiety of complex **122** are responsible for the high enantioselectivity observed.^311^

**Scheme 27:**
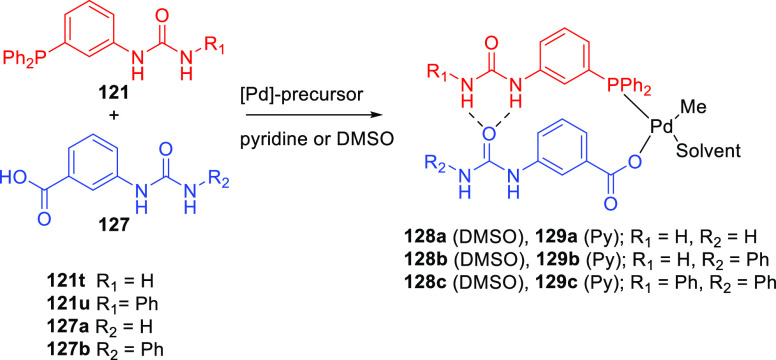
Self-Assembly of Anionic Urea-Containing Ligands and UREAPhos Ligands to Generate a Small Library of Palladium Complexes (**128a**–**c** and **129a**–**c**) (Solvent Is DMSO or Pyridine, Respectively)

Supramolecular hydrogen-bonded bidentate ligands using the urea binding motif were also exploited to generate the self-assembled *hetero*-bidentate ligands illustrated in Scheme 27. Using UREAphos ligands **121t** or **121u** and anionic urea-containing ligands **127a**–**b**, a small library of palladium complexes was generated (**128a**–**c** and **129a**–**c**), forming a supramolecular SHOP-like Pd complex.^316^ The existence of the hydrogen-bonded bidentate structure was confirmed by an array of spectroscopic and DFT studies. Catalysts **128** (with DMSO as solvent) were applied in the Pd catalyzed ethylene polymerization, and **128a** was found to be the most active catalyst, leading to the production of highly branched polyethylene with a molecular weight of 55.700 g/mol and melting temperature of 112 °C.

**Scheme 28:**
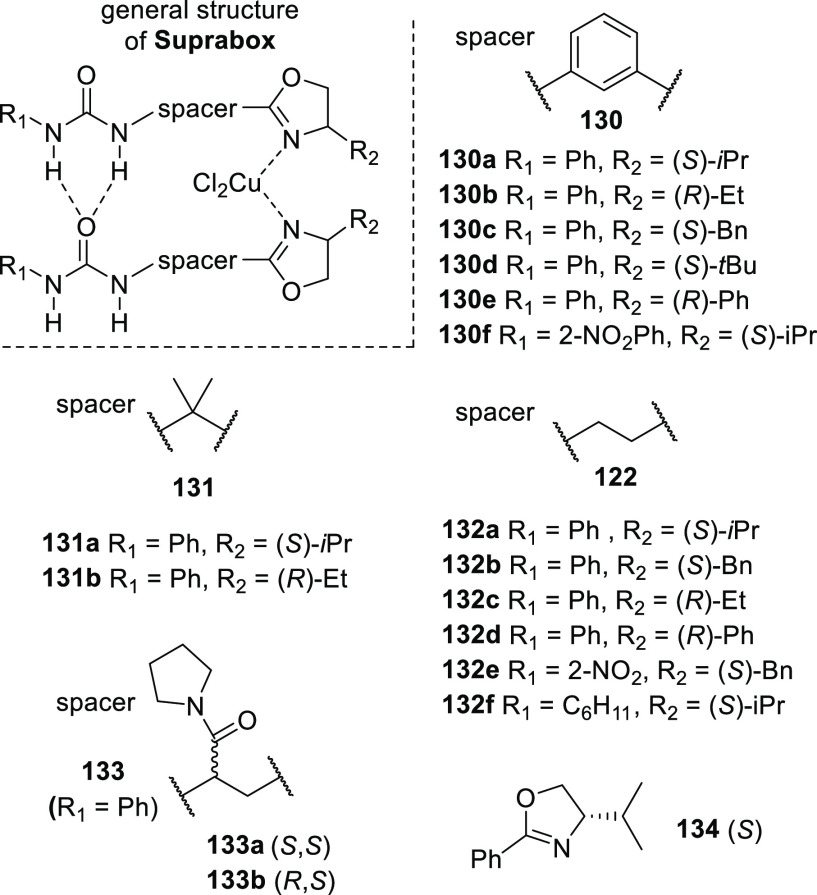
Overview of Some Suprabox Ligands Used in the Copper Catalyzed Asymmetric Benzoylation of *vic*-Diols^317^

Further diversification of the coordination moiety of the urea-based ligand building blocks was done by Piarulli and co-workers by generating the urea-based oxazoline SupraBox ligands illustrated in Scheme 28. They applied these in the copper catalyzed asymmetric benzoylation of *vic*-diols.^317^ Variable temperature ^1^H NMR spectroscopy and dilution studies of [Pd(**132a**)_2_Cl_2_] in CD_2_Cl_2_ were used to probe the supramolecular interaction between the two monomers and confirmed the hydrogen bonding between the urea moieties within the complex. The application of these self-assembled ligands in asymmetric benzoylation of *vic*-diols gave interesting enantioselectivities (up to 86% with ligand **132c**) but lower than the traditional bidentate complex (*R*,*R*)-PhBox (99% e.e.). Nonetheless, the importance of the hydrogen bonding motif was underlined by the generation of racemic product when a monomer (**134**) lacking hydrogen bonding ability was used.

#### 3.2.2 6-DDPon Ligands

**Scheme 29:**
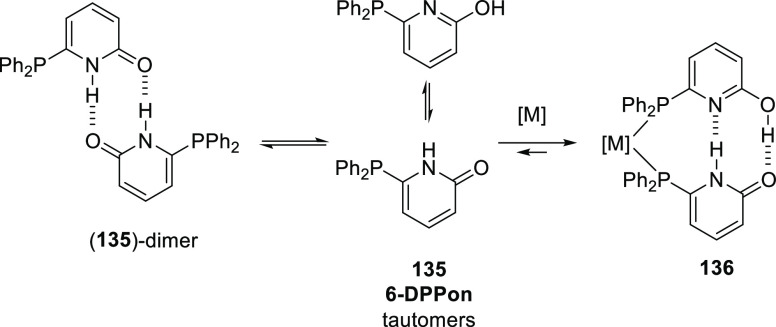
Tautomeric Structure of 6-DPPon (**135**) and Self-Assembly in the Absence and Presence of a Transition Metal

In 2003, Breit and co-workers described the formation of supramolecular bidentate ligands using 6-DPPon (**135**) shown in Scheme 29, which is based on the pyridone/hydroxypyridine tautomers (**135**)^318^ that can form a dimer by a DA/AD HB array (see also section 2.2, Figure 2). The formation of the supramolecular bidentate ligands is driven by coordination of the monomers to the metal center, and the supramolecular bidentate structure was initially confirmed by X-ray structure analysis of *cis*-[PtCl_2_(6-DPPon)_2_] (**136**). Later, also a rhodium complex was characterized by X-ray analysis, showing the hydrogen bonding between the ligand building blocks.^319^ Based on NMR spectroscopic experiments, enthalpic stabilization through hydrogen bonding was found to contribute 14–15 kcal·mol^–1^ to the complex formation.^320^ With bite angles close to 105° and being relatively electron poor, these ligands have found application in multiple catalytic transformations.

##### 3.2.2.1 Hydroformylation

The self-assembled bidentate ligands were evaluated in the rhodium catalyzed hydroformylation of 1-octene (and other *n*-alkenes), and these catalysts displayed high linear/branch selectivity (l:b ratio 97/3). These catalysts were significantly more active than known bidentate phosphine ligands such as the *tert*-Bu-Xantphos ligand, which is a well-known wide bite angle ligand that can form both *cis* and *trans* spanning coordination complexes.^321^ Interestingly, the catalytic system kept its selectivity up to 100 °C, suggesting that the HBs hold at elevated temperatures. A lower selectivity (similar to that of complexes based on PPh_3_) was obtained when the complex was used at even higher temperatures or when methanol was used as solvent, indicating that under these conditions the HB motif is disrupted. A detailed study of the hydroformylation reaction using ESI-MS identified crucial intermediates of the Heck–Breslow catalytic cycle in which the RO–H···O=C HB remains intact.^322^ Interestingly, in these gas-phase reactions, oxidative addition of the H–N moiety provides additional pyridine coordinated intermediates which are involved in the hydrogen activation process. DFT calculations of the parent system complex [HRh(CO)(6-DPPon)_2_] indeed confirm that the O–H···O=C HB is much stronger than the N–H···N hydrogen bonding interaction.^319^ The weaker N–H···N is thus disrupted to a great extent in the course of the catalytic cycle, making the H–N moiety accessible for the proposed oxidative addition.

**Scheme 30:**
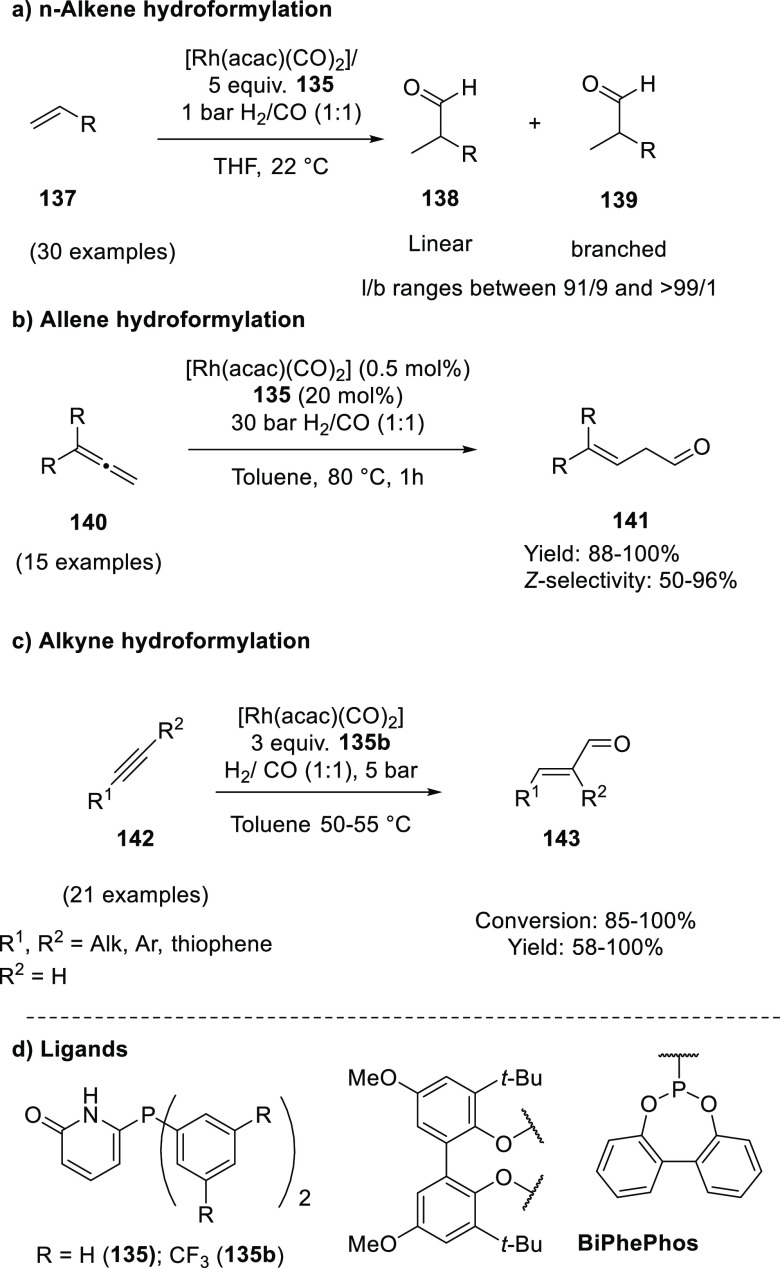
Rh/6-DPPon Catalyst Used in (a) *n*-Alkene Hydroformylations, (b) 1,1-Disubstituted Allene Hydroformylation, and (c) Alkyne Hydroformylation. (d) The Used Ligands (**135** and **135b**) Are Shown Together with a Covalent Bidentate Ligand That Was Used for Comparison (BiPhePhos)

The high activity/selectivity allowed hydroformylation to be effectively performed at room temperature and just 1 bar syngas pressure, alleviating the need for high pressure equipment.^323^ This practicality was displayed by converting a large substrate scope consisting of 30 *n*-alkenes, as illustrated in Scheme 30a. The alkenes were equipped with many important functional groups, and the aldehyde products were obtained in high yields with l/b selectivities between 91/9 and >99/1. Direct comparison of the complexes based on 6-DPPon (**135**) and “BiPhePhos” (Scheme 30d) showed that at similar conversion and selectivity, the 6-DPPon (**135**)-based system displayed lower isomerization than the BiPhePhos (8% vs 54%) at 22 °C and 1 atm of syngas (H_2_/CO = 1:1). Interestingly, similar reactivity and selectivity were obtained in aqueous hydroformylation reactions in the presence of an emulsifier, performed at 1 bar syngas pressure and room temperature.^324^

In a separate study, the Rh/(**135**) system was found to be active and selective in the hydroformylation of 1,1-disubstituted allenes (**140**), as shown in Scheme 30b.^325^ Recently, the Rh/(**135**) catalyst system was also applied to the sequential double hydroformylation of butadiene by Subramaniam and co-workers.^326^ The traditional bidentate ligand DIOP gave the best results in the first hydroformylation step, providing a maximum 4-pentenal selectivity of 48%. The use of **135** as ligand showed the best performance for the subsequent 4-pentenal hydroformylation step with adipaldehyde selectivity exceeding 93%.

The slightly modified Rh/(**135b**) catalyst system in which 3,5-(trifluoromethyl)phenyl substituents are placed on the phosphine ligand also proved to be efficient in the hydroformylation of internal alkynes (**142**), achieving high selectivity and activity (see Scheme 30c).^327^ The observed reactivity and selectivity were somehow lower using alkyne substrates with electron-poor aryl groups (R^1^ and R^2^ = 4-CF_3_Ar, conversion 83%, yield 58%). The conversion of aromatic terminal alkynes was also reported.

**Scheme 31:**
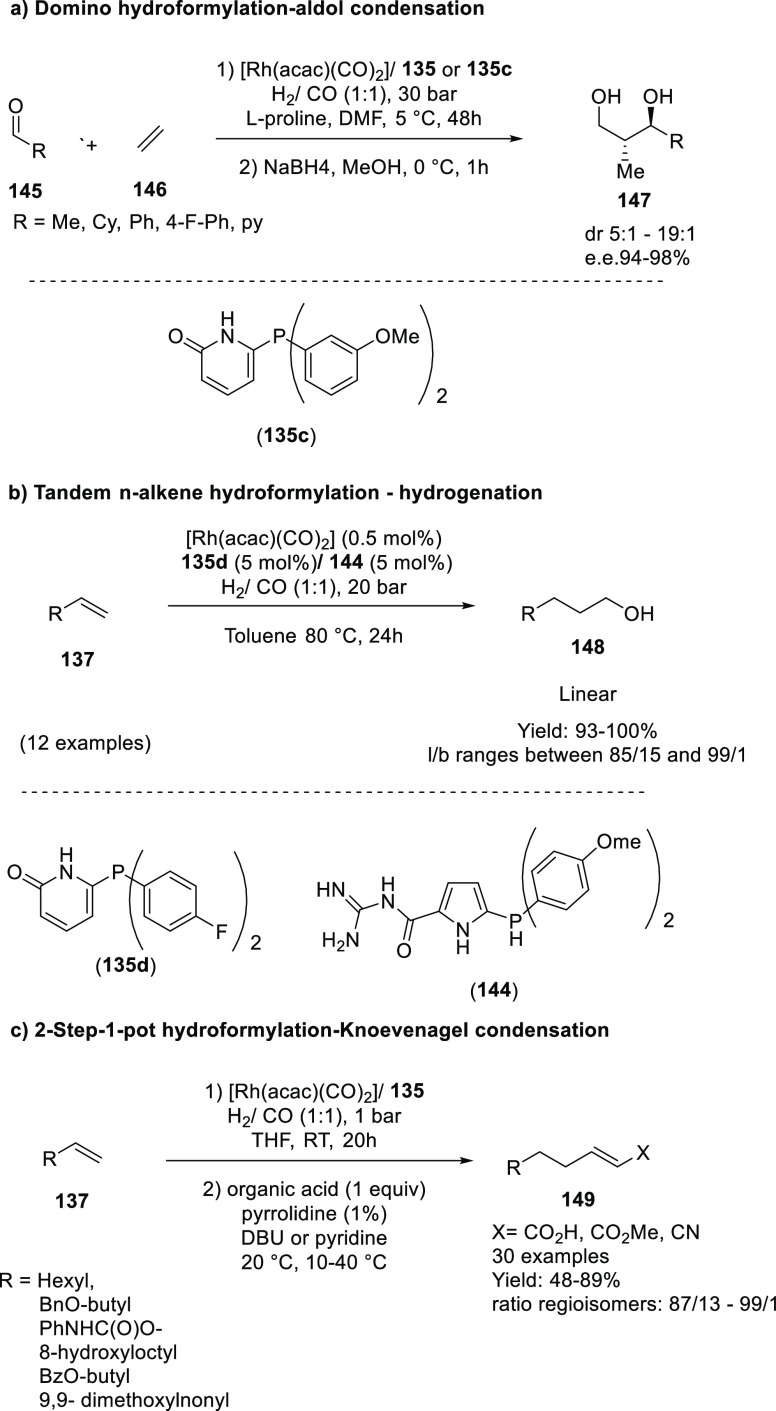
6-DPPon (**135**) Ligand System Used in (a) Domino Hydroformylation-Aldol Condensation, (b) Tandem *n*-Alkene Hydroformylation-Hydrogenation, (c) and the Regioselective Hydroformylation-Decarboxylative Knoevenagel Reaction

Interestingly, the Rh/6-DPPon system was found suitable for the low temperature domino hydroformylation/l-proline catalyzed cross-adol addition shown in Scheme 31a. To prevent a buildup of the primary hydroformylation aldehyde product (leading to undesired homocoupling of the aldehyde), the rate of the hydroformylation reaction was attenuated by using **135c** in the domino hydroformylation/aldol condensation reaction. When salicylaldehyde was used as the acceptor aldehyde, the corresponding lactols were isolated. The reaction was performed in DMF, but the authors did not provide any information on the stability of the HB network of the catalytic system under these conditions.^328^

The tandem rhodium catalyzed hydroformylation–hydrogenation of alkenes illustrated in Scheme 31b was also reported using the combination of 4-fluorophenyl-6-DPPon (**135d**) and an acyl-guanidine decorated ligand (**144**) (the latter catalytic system will be further described in section 4.1).^329^ The Rh/6-DPPon catalyst is responsible for the stereoselective hydroformylation and the Rh/(**144**) catalyst for the aldehyde hydrogenation. Fine-tuning of the electronic properties was necessary to match the relative rates of hydroformylation and aldehyde hydrogenation, of which **135d**/**144** was the most efficient combination.

As illustrated in Scheme 31c, the combination of regioselective hydroformylation and decarboxylative Knoevenagel condensation allows for two-step, one-pot C3 homologation of terminal alkenes to (*E*)-α,β-unsaturated acids and esters, (*E*)-β,γ-unsaturated acids, (*E*)-α-cyano acrylic acids, and α,β-unsaturated nitriles.^330^ The scope of the reaction was illustrated by 30 examples in which the initially formed aldehydes (by hydroformylation) were reacted with malonic acid, malonic acid methyl ester, or nitrilacetic acid in the presence of pyridine or DBU (1,8-diazabicyclo[5.4.0]undec-7-ene). The overall yield varied between 48 and 99%, and the regioselectivity ranged between 87/13 and 99/1, strongly depending on the base used.

##### 3.2.2.2 Hydrogenation

**Scheme 32:**
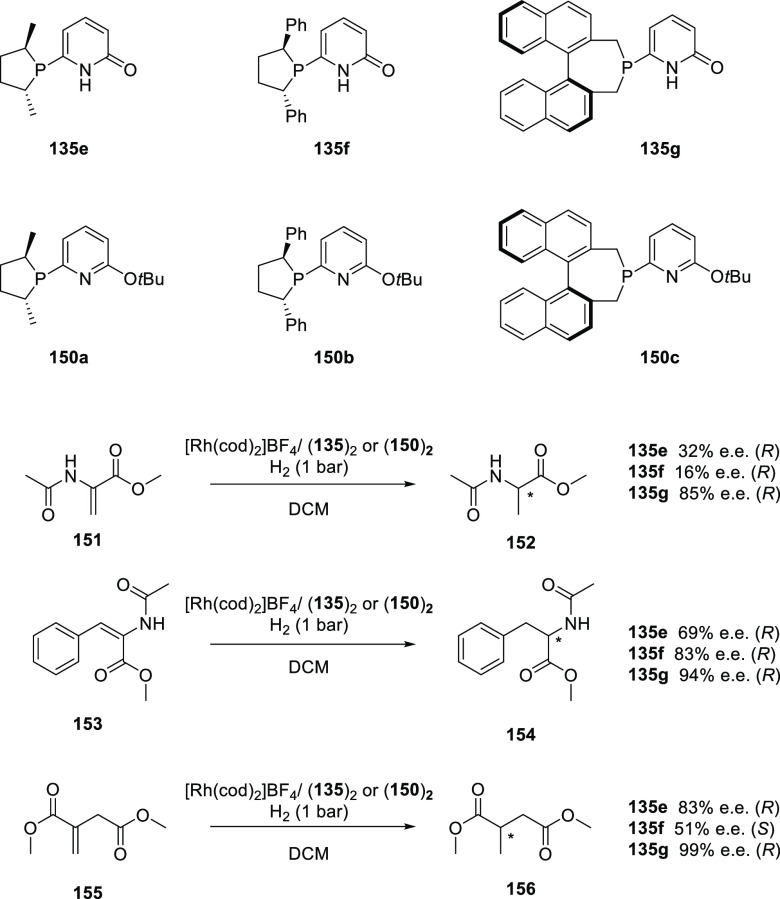
Phospholane-Based 6-DPPon Ligands (**135e**–**g**) and Ligands (**150a**–**c**) Used in the Rhodium Catalyzed Asymmetric Hydrogenation

Rh/(**135**) systems based on chiral phospholane analogues have been evaluated in the asymmetric hydrogenation of a number of benchmark substrates.^331^ As is depicted in the top of Scheme 32, the different building blocks vary in steric/size at the phospholane unit. Ligands (**150a**–**c**) which are incapable of forming hydrogen-bonded bidentates were included as reference. Application of the self-assembled bidentate ligands (**135e**–**g**) in the rhodium catalyzed hydrogenation of methyl acetamidomethacrylate (**151**), methyl acetamidocynnamate (**153**), and dimethylitaconate (**155**) showed that the (**135g**) performed best for all three substrates in terms of enantioselectivity (with 91%, 94%, and 99% e.e., respectively). A noticeable loss of enantioselectivity in the conversion of methyl acetamidocynnamate and dimethylitaconate was observed when the reaction was performed in methanol instead of DCM, suggesting loss of the HB bidentate structure. DFT calculations on ligands **135e**–**g** show that phospholane-substituted 2-hydroxypyridines are lower in Gibbs free energy and are therefore the dominant tautomers in the gas phase. Compared to the parent 2-pyridone/2-hydroxypyridine system, the equilibria for **135e** and **135f** are shifted to the 2-hydroxypyridine side, while that of **135g** changes only slightly. Calculations of Rh/(**135g**)_2_, with a relatively large steric bulk, favor the formation of the supramolecular bidentate ligand, compared to Rh/(**135e**)_2_ and Rh/(**135f**)_2_ (with Δ*G* of 14.3, 7.9, and 3.2 kcal·mol^–1^, respectively).

**Scheme 33:**
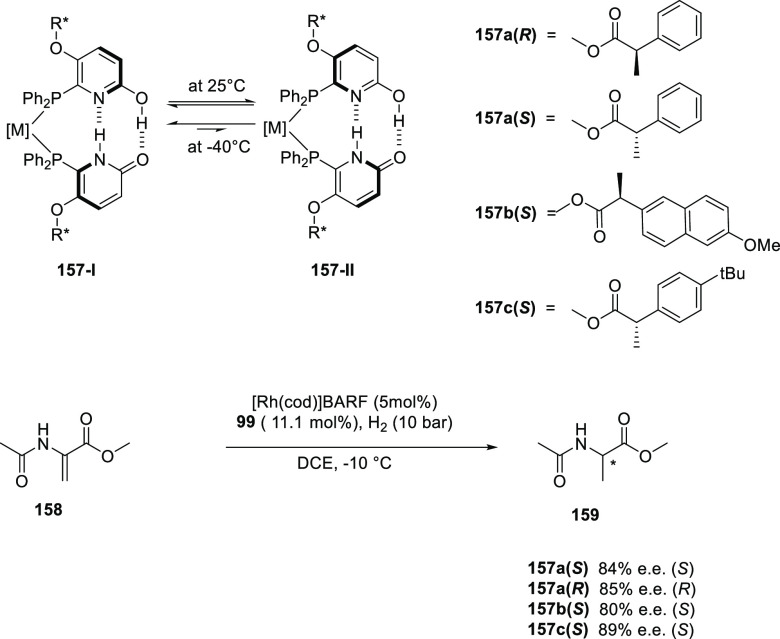
Atropoisomeric Supramolecular Hydrogen-Bonded 6-DPPon-Based Ligands (**157**) and Their Application in Rhodium Catalyzed Asymmetric Hydrogenation of Methyl Acetamidomethacrylate (**158**)

Recently, the library of chiral 6-DPPon building blocks was extended to analogues with a chiral functional group at the 5 position of the pyridine ring, as illustrated in Scheme 33.^332^ These self-assembled bidentate ligands are not planar but twisted around the HB motif, leading to atropisomeric structures, and the presence of additional chiral groups results in the formation of diastereotopic species. These diastereoisomers interconvert rapidly at room temperature but can be spectroscopically resolved at low temperature (−40 °C), showing the presence of a dominant species. Application of this ligand system (**157**) in asymmetric hydrogenation of methyl acetamidomethacrylate (**158**) gave interesting enantioselectivity (52% e.e.) at room temperature in 1,2-dichloroethane which can be further improved to 86% e.e. when performing the hydrogenation reaction at −30 °C. By changing the solvent to dichlorobenzene, an e.e. of 90% could even be obtained. The low activity and selectivity observed for this catalytic system in THF and MeOH are in line with the low amplitude of the circular dichroism spectra observed in those solvents compared to the circular dichroism spectra observed in 1,2-dichloroethane. This example shows the potential of chirality induction in the catalytic system through the HB network.

#### 3.2.3 Heterobidentate Ligands 6-DPPAP and 3-DPPICon

**Scheme 34:**
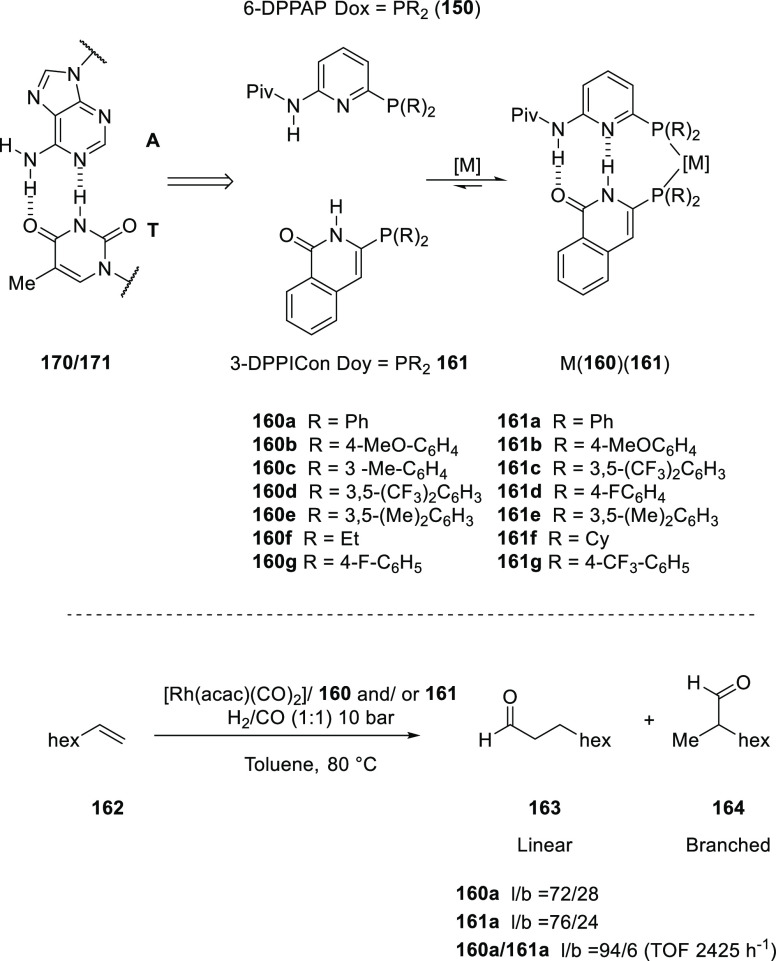
Supramolecular Heterobidentate Ligands Based on Complementary Hydrogen-Bonded Ligand Building Blocks, Inspired by the A-T DNA Pairs A combinatorial library of 4 × 4 supramolecular heterobidentate ligands becomes accessible based on a 4 + 4 building block.

In analogy to the adenine-thymine DNA base paring illustrated in Scheme 34, the supramolecular heterobidentate ligands based on aminopyridine (**160**)/isoquinolone (**161**) have been developed by Breit and co-workers.^333^

Mixing of 1 equiv of 6-diphenylphosphino-*N*-pivaloyl-2-aminopyridine (6-DPPAP; **160a**) with 1 equiv of 3-diphenylphosphinoisoquinolone (3-DPPICon; **161a**) in the presence of [PtCl_2_(COD)] yielded the heteroleptic *cis*-[PtCl_2_(**160a**)(**161a**)] in quantitative yield. The ligands **160** and **161** were evaluated in the rhodium catalyzed hydroformylation of terminal alkenes and showed that only the combination of 6-DPPAP (**160a**) and 3-DPPICon (**161a**) displayed a much higher linear/branched selectivity compared to the reaction employing either **160a** or **161a** alone. Exploration of a ligand library showed that the activity expressed in TOF varied between 1040 and 8643 mol_substrate_/mol_catalyst_·h^–1^. The fastest catalyst was based on **160d** and **161d**, and very small changes in the selectivity were noted, as detailed in Table 4. In addition, LyondellBasell has patented the Rh/6-DPPAP/3-DPPICon system for the selective hydroformylation of allyl alcohol into 4-hydroxybutyraldehyde.^334^ The combination Rh/**160e**/**161e** gave the highest aldehyde yield (99%) and highest linear selectivity (linear/branched ratio 23.1). Interestingly, the combination Rh/**160f**/**161f** mainly produced the linear alcohol by a hydroformylation–hydrogenation sequence (alcohol yield 79%, l:b ratio 9.7).

**Table 4:** Rhodium Catalyzed Hydroformylation Using a 4 × 4 Library of 6-DPPAP (**160a**–**d**) and 3-DPPICon (**161a**–**d**) Derivatives (for Structures See Scheme 34)^a^ with the Activity in TOF^b^ and Selectivity in l:b Ratio Provided^c^

	3-DPPIcon monomer			
6-DPPAP monomer	**161a** (R = Ph)	**161b** (R = (4-MeOPh)	**161c** (R = 3,5-bis-CF_3_Ph)	**161d** (R = 4-FPh)
**160a** (R = Ph)	2425	1040	2732	2559
	94:6	94:6	96:4	95:5
**160b** (R = 4-MeOPh)	2033	1058	1281	1772
	93:7	92:8	96:4	94:6
**160c** (R = *m*-tolyl)	3537	1842	1808	2287
	94:6	93:7	96:4	94:6
**160d** (R = 3,5-bis-CF_3_Ph)	7439	2695	7465	8643
	94:6	95:5	94:6	94:6

a Reaction conditions: [Rh(CO)_2_(acac)], [Rh]:(**160**):(**161**):1-octene = 1:10:10:7500, 10 bar H_2_/CO (1:1), toluene (*c*_0(1-octene)_ = 2.91 M), 5 h. Catalyst preformation: 5 bar CO/H_2_ (1:1), 30 min, RT to 80 °C.

b Turnover frequency (TOF) in mol_substrate_/mol_catalyst_·h^–1^.

c Regioselectivity in linear (l) to branched (b) (l:b) ratio.

**Scheme 35:**
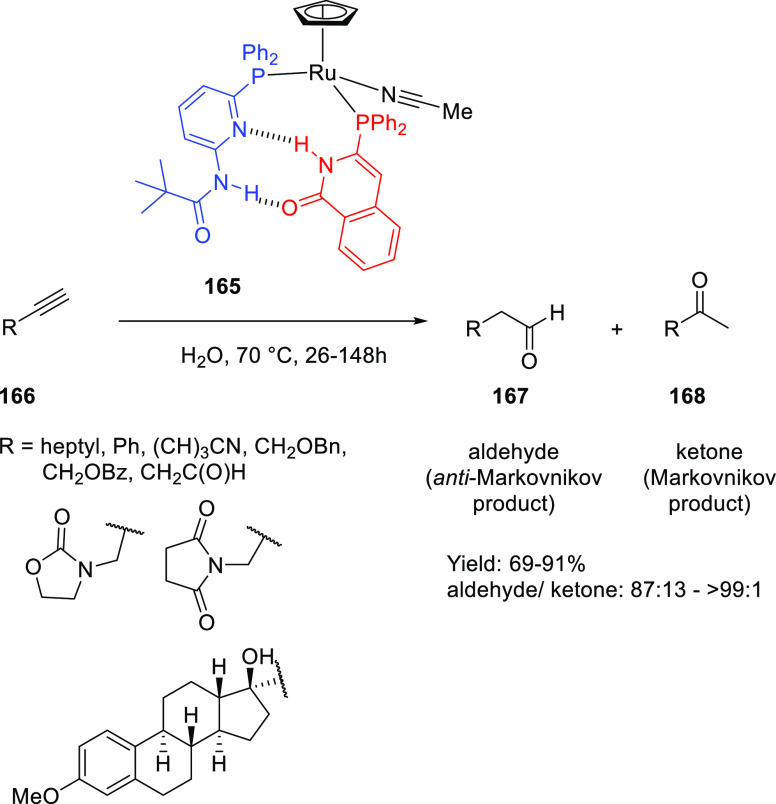
[RuCp(**160**)(**161**)(MeCN)] (**165**) Catalyzed *anti*-Markovnikov Hydration of Terminal Alkynes (**166**)

Ruthenium complexes based on the supramolecular heterobidentate ligands 6-DPPAP (**160**) and 3-DPPICon (**161**) were also investigated in the ruthenium catalyzed hydration of 1-nonyne.^335^ In a preliminary study, different [RuCp(L)_2_(MeCN)] complexes with L_2_ being traditional and supramolecular bidentate ligands were evaluated. The complexes based on the traditional ligand and the homosupramolecular bidentate 6-DPPon (**135**) performed poorly, leading to formation of the Markovnikov product, while the self-assembled heterobidentate complex [RuCp(**160**)(**161**)(MeCN)] gave high conversion and perfect selectivity for the aldehyde (*anti*-Markovnikov product; see Scheme 35).

**Scheme 36:**
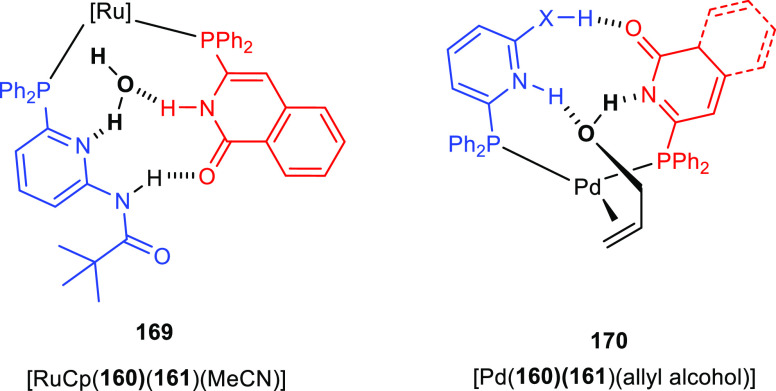
HB Involvement with a Supramolecular Bidentate Ligand during a Catalytic Cycle in (a) Activation of a Water Molecule in the Hydration of Alkynes (See Also Scheme 35) and (b) Activation of Allyl Alcohol for Allylation Reactions

As is illustrated in Scheme 36 (left), the selectivity arises from both the bidentate character of the ligand and an interaction of the functional groups of the complex with the water substrate.^336^ DFT calculations correlated with previous experimental data resulted in the authors proposing a mechanism involving an initial alkyne–vinylidene tautomerism, which occurs via a ligand-assisted proton shuttle mechanism. The HB between the pivaloyl moiety and the isoquinolone remains intact while a water molecule intercalates in the other HB (see 169 in Scheme 36). This example highlights once more the various roles that the functional groups of the HB motif can have, such as leading to the formation of a supramolecular bidentate ligand interacting with the substrate during the catalytic cycle.

A similar hydrogen bond activation of the unreactive allyl alcohol substrate is shown on the right side of Scheme 36 and has been proposed for the palladium catalyzed allylation of indoles and pyrroles with allyl alcohol.^337^ A ligand library of 6-DPPon (**135**), a heterobidentate combination using 6-DPPAP (**160**), and 3-DPPICon (**161**) was evaluated. The library was extended with 6-DPAIND (**171**) and 2-DPPAT (**172**), which also have complementary HB motifs. The catalyst system consisting of [Pd(η^3^-allyl)(COD)]/(**135a**)_2_ provided the most efficient catalyst, yielding the 3-subsituted allyl indoles in 58–91% yield. The allyl alcohol activation is proposed to be based on a HB between the alcohol and the HB motif of the supramolecular bidentate ligand (see Scheme 36). The application of the bidentate ligand based on 6-DPPAP and 3-DPPICon was also explored in the ruthenium catalyzed nitrile hydration.^338^ The highest activity was observed for [Ru(**160a**)(**161a**)(acac)_2_] and was rationalized based on electronic factors. Furthermore, it was hypothesized that the nucleophilic attack of water may be facilitated by hydrogen bonding with the ligands, as was also found for Ru complexes used in the hydration of alkynes (see Scheme 35).

A catalyst library consisting of aminopyridine (**160a**–**b**, **d**, and **g**) ligands and isoquinolone ligands (**161a**–**d** and **g**) was also evaluated in the nickel hydrocyanation of styrene.^339^ Several complexes based on supramolecular heterobidentate ligands were found catalytically active, and the performance depends more on the nature of the isoquinolone than on the aminopyridine building block. The nickel complex based on **160b/161b** gave yields greater than 95% and branched/linear ratios up to 97:3.

**Scheme 37:**
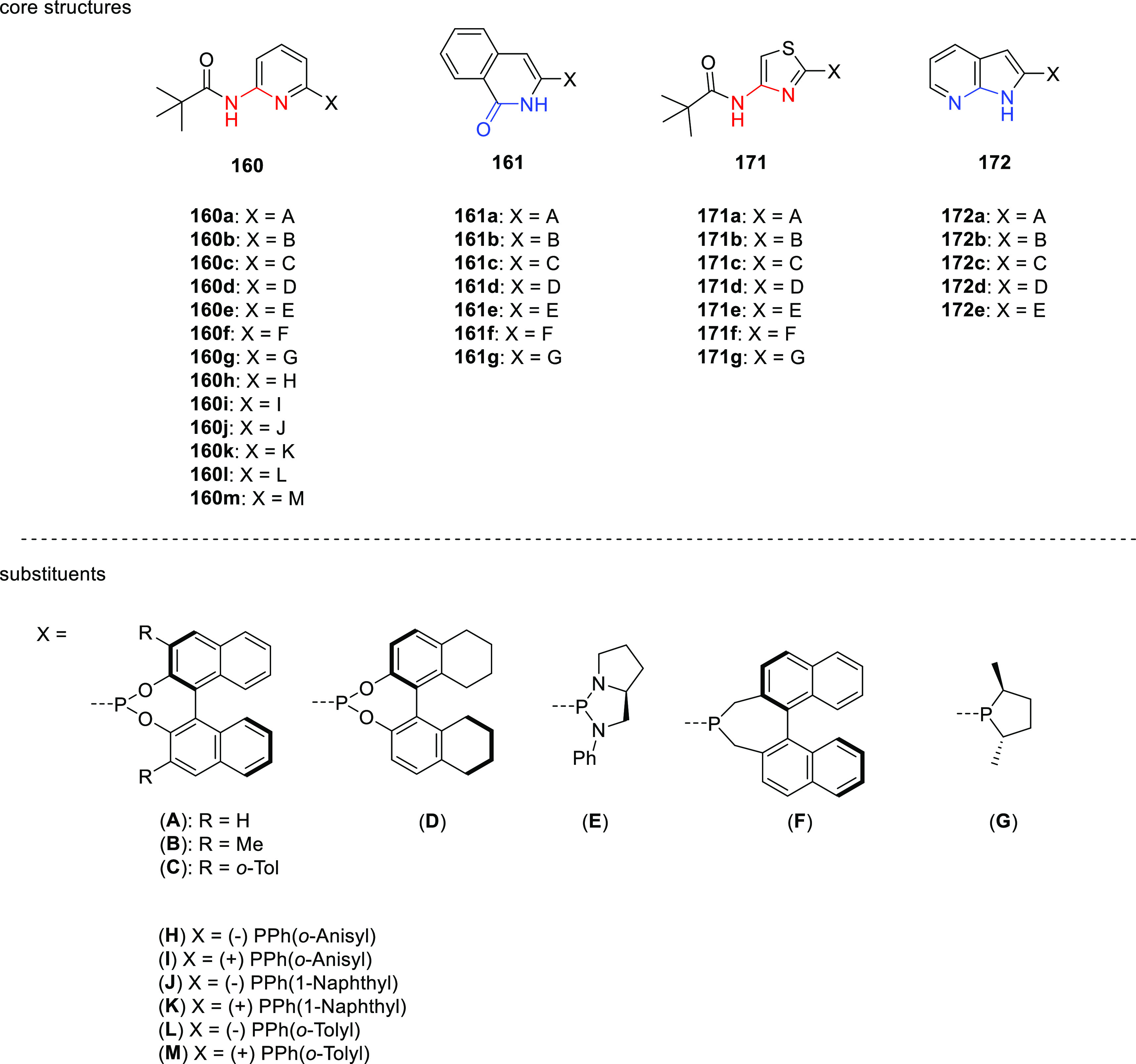
Overview of the Different Ligand Building Blocks with Complementary HB Motifs. Top: Core Structures of Ligands **160**, **161**, **171**, and **172**. Combination with Phosphorus Groups **A**–**G** (Bottom) Leads to a Library of 120 Supramolecular Hetereobidentate Ligands That Was Used in the Combinatorial Iterative Library Deconvolution Strategy. In Addition, Core Structure **150** Has Been Combined with Substituents **H**–**M**

A small library of ligand building blocks with chiral phospholane units that self-assemble into supramolecular homobidentate ligands was explored in the palladium catalyzed Tsuji–Trost reaction and compared to ligand building blocks that are protected and thus does not form self-assembled ligands. Both the self-assembled ligands and the building blocks that are coordinated as monodentate ligands form complexes that induce enantioselectivity. However, the smaller ligand based on **E** (see Scheme 37) that does not form self-assembled bidentate ligands provided the highest e.e.’s (99% e.e.).^341^

The same HB motif was used to make a library with chiral phosphine and phosphinite ligand building blocks, providing supramolecular bidentate ligands that were explored in rhodium catalyzed asymmetric hydrogenation (see, for example, the ligand library in Scheme 37). Formation of Rh**L**_**1**_/**L**_**2**_COD complexes based on these supramolecular bidentate ligands was confirmed by NMR and MS spectroscopy.^340^ The library of chiral self-assembled catalysts consisting of chiral phosphines and phosphonites was formed by just mixing the components and explored in asymmetric hydrogenation. The phosphonite systems based on the BINOL skeleton resulted in the catalysts that induce the highest selectivity. For example, high enantioselectivities (>99% e.e.) and excellent catalyst activities were observed in the asymmetric hydrogenation of methyl acetamidoacrylate using [Rh(COD)_2_]BF_4_/**160a**/**161a** as catalyst. In a follow-up study, the solvent was found to have an influence, but no correlation could be found to the HB characters of the ligands.

The full potential of the approach was demonstrated by the generation of a 10 × 12 library of supramolecular bidentate ligands.^342^ The ligand building blocks were based on the acceptor–donor hydrogen bonding units (in red) 6-DPPAP (**160**, with **A–G**) and **171** (with **A–G**) and the donor–acceptor hydrogen bonding units (in blue) 3-DPPICon (**161**, **A–G**) and **172** (**A–E**) as illustrated in Scheme 37. By just mixing these building blocks, a large library of 120 self-assembled bidentate ligands was available that could be evaluated individually. Next to individual testing, the screening for the best catalyst was also exploited through an iterative library deconvolution strategy.

The combinatorial iterative library deconvolution strategy involves the division of the catalysts into subgroups that are evaluated as a mixture, thereby accelerating the evaluation of the entire library’s potential. As the ligand building blocks form the supramolecular bidentate ligands *in situ*, such mixtures are easy to prepare. With this concept in mind, the activity and selectivity of mixtures of catalysts were probed in the rhodium catalyzed asymmetric hydrogenation of methyl acetamidomethacrylate. The mixture of catalysts that provided the highest selectivity was used in the next set of experiments, in which the mixtures consisted of a smaller number of catalysts, based on the building blocks present in the winner subset of the first round. In the final set of experiments, four reactions were performed with pure catalyst solutions to determine which supramolecular bidentate ligand gave the complexes that produced the product in the highest enantioselectivity. In this particular example, the strategy identified three catalysts that yield full conversion and 99% e.e. of the product. Importantly, only 17 reactions were required using this iterative library deconvolution strategy to identify the best catalyst from the set of 120. This combinatorial strategy was further applied to other substrates with similar results.

#### 3.2.4 Ligands Based on Other HB Motifs

**Scheme 38:**
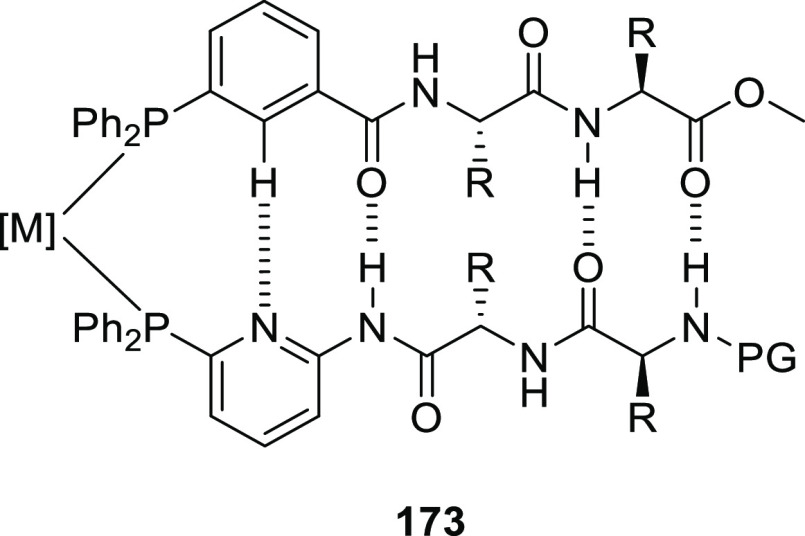
General Structure of SupraPeptiPhos (**173**), a Supramolecular Bidentate Ligand Based on a Peptide Chain That Mimics the Antiparallel β-Sheet Structures

The supramolecular bidentate ligands based on one or two HBs were demonstrated to provide selective catalysts for a variety of transformations. Extension to motifs based on a larger array of HBs is interesting, as this would make the system more rigid. The motifs based on AAAA-DDDD arrays would provide the strongest interaction (see also Figure 2); however, synthetic procedures to access such building blocks seem complicated. To form supramolecular bidentate ligands based on a larger HB array, the application of peptidic chains has been explored. The peptide sequence chosen is shown in Scheme 38 and mimics the typical β-sheet structures found in proteins.^343^ This set of ligand building blocks that form bidentate ligands was coined SupraPeptiPhos (**173**). Self-assembly of PtCl_2_ with **173** was monitored by ^1^H NMR spectroscopy, which indeed revealed the formation of a β-sheet-type structure. Moreover, **173** was applied in the rhodium catalyzed asymmetric hydroformylation of styrene, resulting in an e.e. of up to 38%. While this is modest, these data do provide evidence of chirality transfer from the peptidic chain to the product via the metal center, as highlighted earlier for the work of Kirin (see Scheme 24).

## 4 Substrate Orientation by Hydrogen Bonding

Metal catalyzed transformations are typically described as a sequence of elementary steps that occur at the metal center.^344^ Depending on the specific details, the selectivity of the reaction is determined during one of these steps. In many reactions, a migration step is involved in the selectivity determining step, such as in hydrogenation and hydroformylation reactions. The orientation of the substrate at the metal center plays an important role in such a selectivity determining step. The substrate orientation at the metal center depends on the ligand environment around the metal center and can also be realized via coordination of a “directing group” to a metal center.^345−350^ Recently, hydrogen bonding between the functional groups of the substrate and the functional groups of the catalyst has been used as a strategy to achieve the proper orientation of the substrate and, hence, to control the selectivity of the reaction. In this manner, the interactions in the second coordination sphere are of crucial importance. In analogy to the substrate orientation effects found in natural systems, such control of the second coordination sphere may further guide the rational design of selective catalysts. The hydrogen bonding interactions between the substrate and the functional groups of the catalyst are ideal for substrate orientation, and in this section we will review examples of this concept in various different reactions.

### 4.1 Asymmetric Hydrogenation

**Figure 6:**
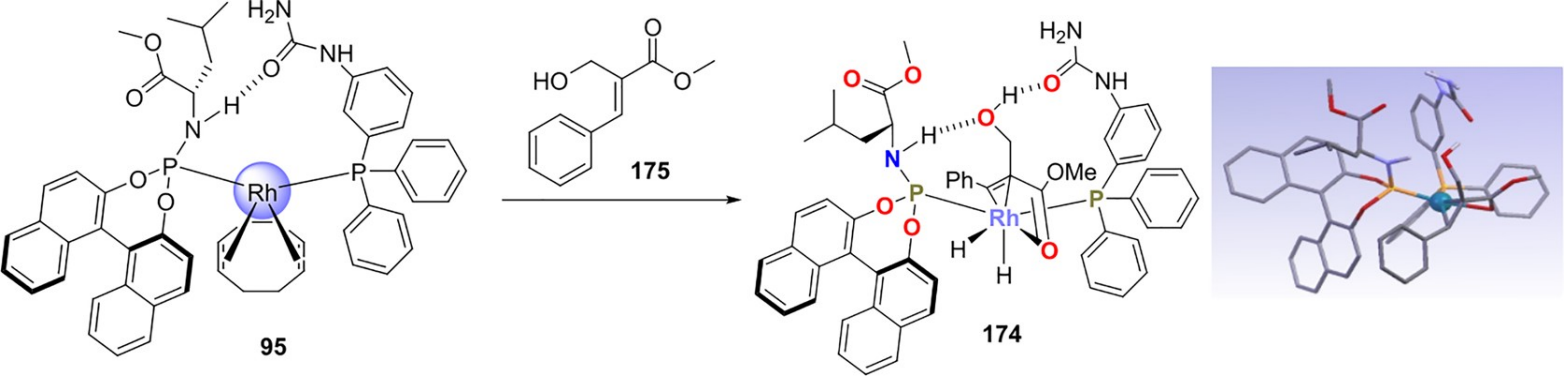
Formation of the substrate coordinated complex, in which the hydroxy function of substrate **175** forms two HBs with the functional groups of the ligands, as indicated by the dotted lines in the schematic representation of the DFT calculated structure.

The asymmetric hydrogenation reaction is a powerful asymmetric transformation, as it provides a general strategy to create chiral centers in organic molecules. This relevance was underscored by the Nobel prize in 2001 awarded to Knowles and Noyori.^1,29^ Kagan and Knowles reported chiral bidentate phosphine ligands that formed rhodium complexes displaying significant e.e. in asymmetric hydrogenation (up to 70% e.e.).^351,352^ These bidentate ligands were further developed to get catalysts displaying exceptionally high enantioselectivities for a large set of substrates. In the early nineties it was also demonstrated that complexes based on monodentate ligands can induce high enantioselectivity. In the early 2000s, the concept of supramolecular bidentate ligands, that is the generation of bidentate ligands based on the self-assembly of ligand building blocks, was reported. These ligands feature the benefit of easy synthesis typically for monodentates, yet they display control over the coordination sphere like bidentate ligands. Also, supramolecular bidentate ligands are ideal for the generation of large catalyst libraries, as the number of catalysts grows exponentially with the number of synthesized building blocks (see also section 3). Our group has developed ligand building blocks that can form supramolecular bidentate ligands based on a single HB between a urea C=O and a phosphoramidite N–H (see section 3.1.2 and Figure 6).^295,353^ Rhodium complexes of these ligands were shown to convert methyl 2-hydroxymethacrylate and its derivatives in very high enantioselectivities, in contrast to substrates that did not have the hydroxy functional group (methyl or methyl ester). This difference in reactivity suggests that hydrogen bonding between the catalyst and the hydroxy group of the substrate plays an important role.

**Figure 7:**
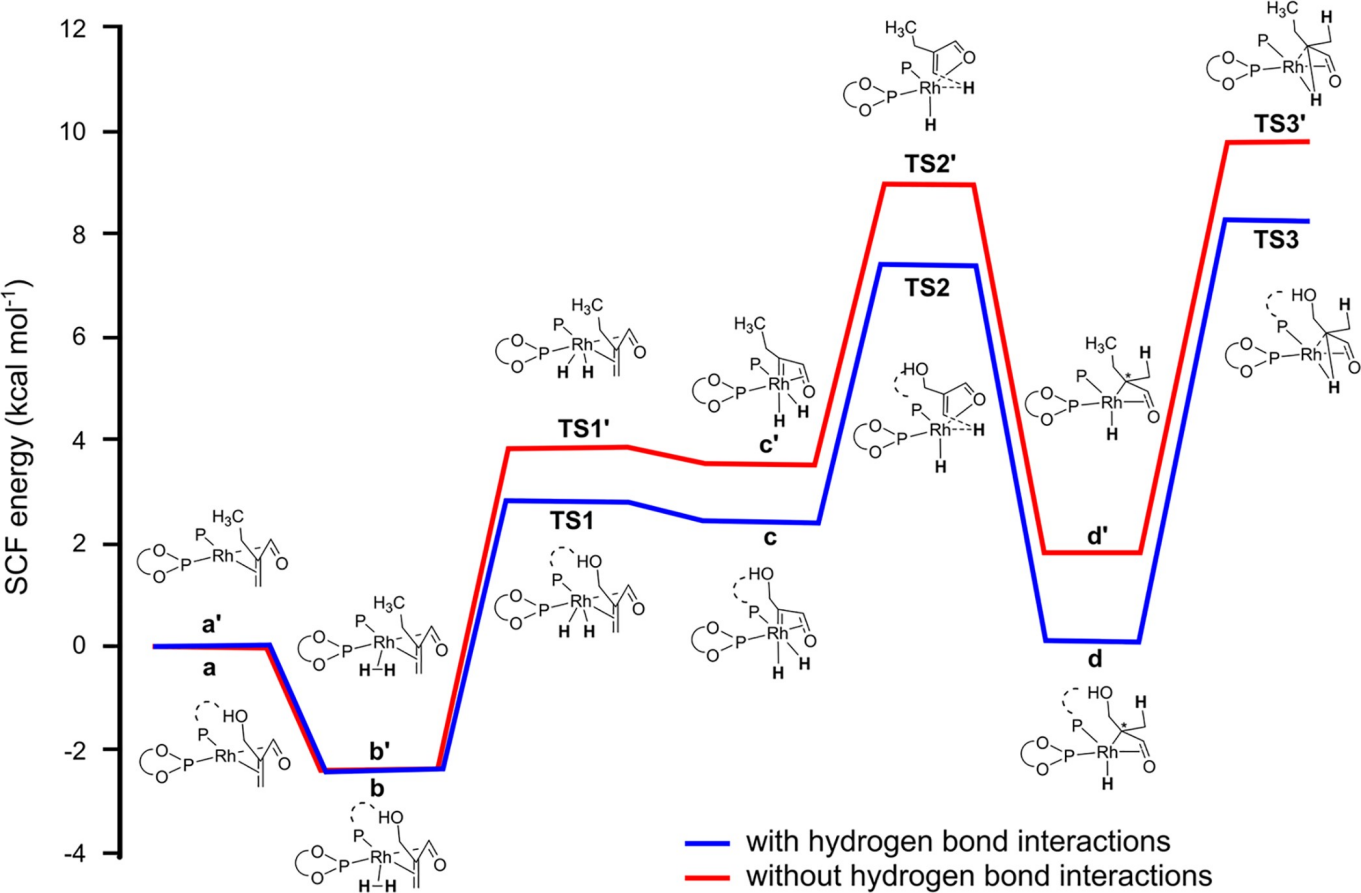
Comparing relative energies of intermediates along the reaction path of a substrate that can form a HB interaction (blue) to that of a substrate that cannot (red).

Detailed spectroscopic studies, in combination with kinetic analysis and DFT calculations, revealed the consequence of the HB formed in the second coordination sphere.^354^ Addition of (*E*)-methyl 2-(hydroxymethyl)-3-phenyl acrylate to a solution of the precomplex (under either hydrogen or the solvato complex prepared from the COD precomplex) results in a structure in which the alkene coordinates to the rhodium center and the hydroxyl group of the substrate binds via hydrogen bonding to the functional groups of the ligands. The complex was observed during catalysis by *in situ* NMR spectroscopy and, therefore, was identified to be the resting state of the reaction. According to DFT calculations, the HBs established between the substrate and the ligands of the complex stay intact throughout the whole catalytic cycle. As illustrated in Figure 7, the reaction pathway deploying a ligand that can form this HB (blue) involves lower overall energy barriers of transition states (**TS1**, **TS2**, and **TS3**) compared to the use of a ligand that cannot use this HB (red, **TS1′**, **TS2′**, and **TS3′**). Detailed analysis of all reaction pathways displayed by the various diastereomeric complexes showed that those forming HBs in the starting complex are favored. As such, these complexes operate via an anti-Halpern model, in which the most energetically favored structure (by hydrogen bonding) is the one that is most productive.

**Scheme 39:**
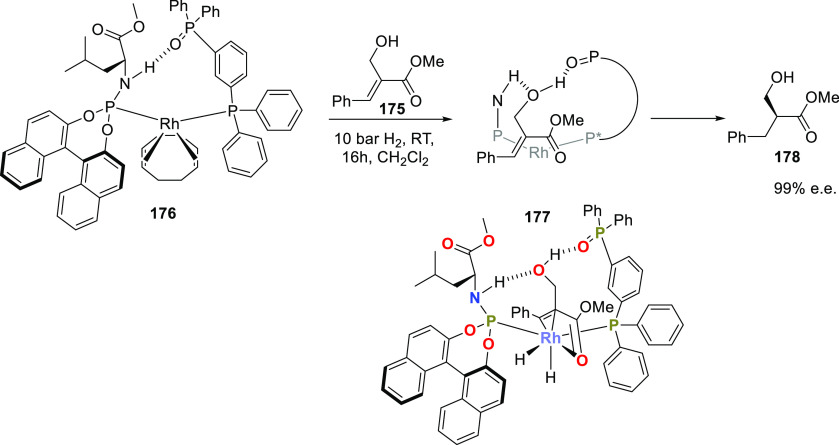
Redesign of the Catalyst, Using Phosphine Oxide-Containing Functional Groups That Are Strong HB Acceptors, Leading to More Active and Selective Catalysts A schematic picture is shown displaying how the HBs are formed during the transition state.

The reaction follows Michaelis–Menten kinetics, and a stronger association (precomplex formation) is due to the HBs between the substrate and the ligands. In addition, these HBs also give a higher *V*_max_, which is in line with the lower transition state barrier. As the HBs formed in the second coordination sphere play a crucial role, another catalyst was designed by only modifying the potential for such hydrogen bonding.^355^ This catalyst is illustrated in Scheme 39 (**176**, left) and consists of a supramolecular bidentate ligand where the urea part in the previous catalyst (see Scheme 38) was replaced by a phosphine oxide, which is a much stronger HB acceptor. A mechanistic study demonstrates that also for this catalyst two HB interactions between the catalyst and the substrate are involved, leading to stabilization of a catalyst–substrate complex intermediate shown at the bottom in Scheme 39 (**177**).

DFT calculations of the reaction pathway show that the stronger HB interactions between the catalyst and the substrate result in a lower energy barrier by transition state stabilization. In line with this, it was found that the second generation catalyst indeed provides higher rates (factor 4). In addition, the product is also generated in higher selectivity (>99% e.e.) and the catalyst is more robust, as demonstrated by its performance at elevated temperatures. Chikkali and co-workers used urea functionalized P-chiral ligand building blocks to form supramolecular bidentate ligands that were used in asymmetric hydrogenation (Scheme 26). For these systems it was also proposed that a HB interaction between the substrate and the urea of the ligand was of crucial importance to steer the outcome of catalysis.^311^

**Figure 8:**
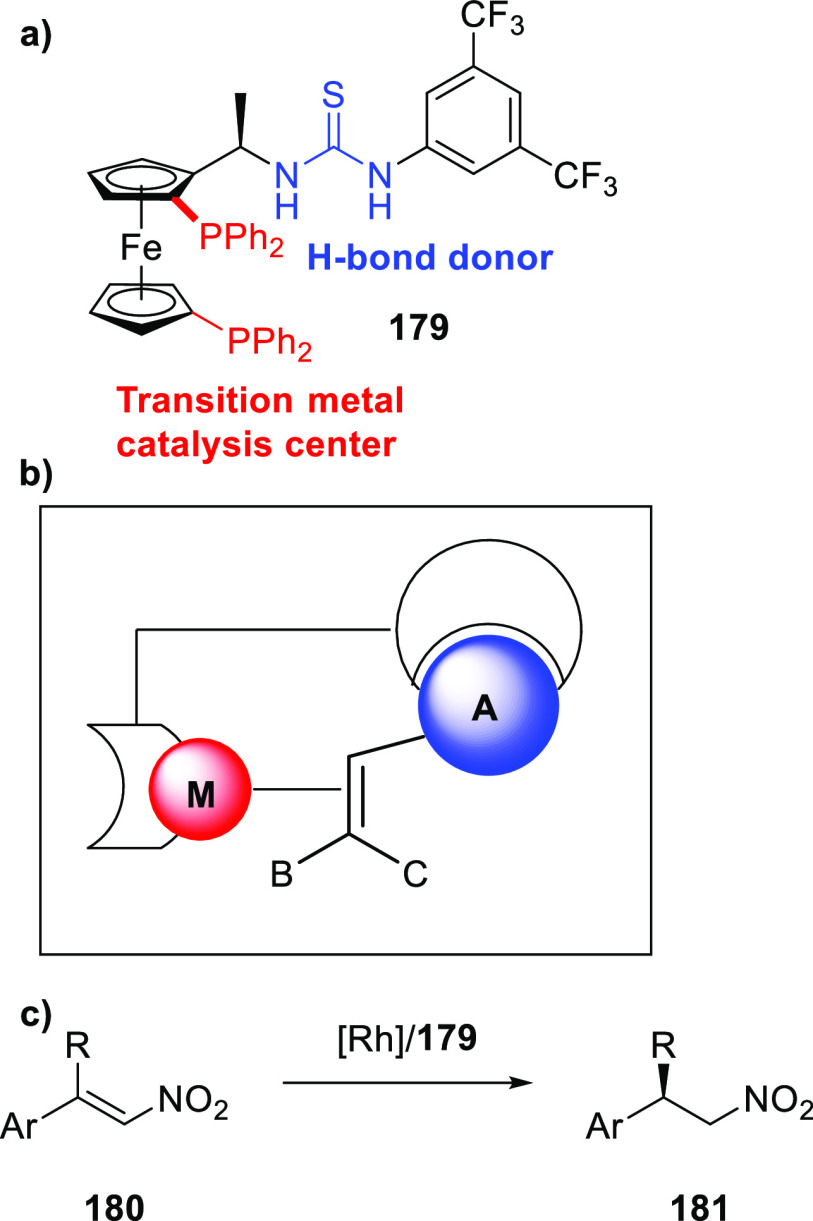
Design of the bidentate phosphine ligand Zhaophos (**179**) with a thio-urea binding site as a handle to organize substrates at the metal center via HBs in the second coordination sphere. Initial studies showed that β,β-disubstituted nitroalkenes (**180**) were converted with high selectivity.

The group of Zhang developed a different strategy for substrate organization by hydrogen bonding in the second coordination sphere. They developed the ligand Zhaophos (**179**) shown in Figure 8, which is based on a ferrocene diphosphine (red) and a thio-urea HB motif (blue).^356^ The ditrifluorophenyl group on the thiourea makes the HB donors a bit more acidic and, as such, even stronger HB donors. Initial studies demonstrated that the Rh–bisphosphine–thiourea complex was an excellent catalyst for the rhodium catalyzed asymmetric hydrogenation of challenging β,β-disubstituted nitroalkenes, providing the product in 99% e.e. for the benchmark methyl-phenyl analogue and above 86% for all analogues reported in the initial study. Control experiments show that the binding site is important, and later spectroscopic and DFT evidence supports this (not reported in the initial study).^357^

**Scheme 40:**
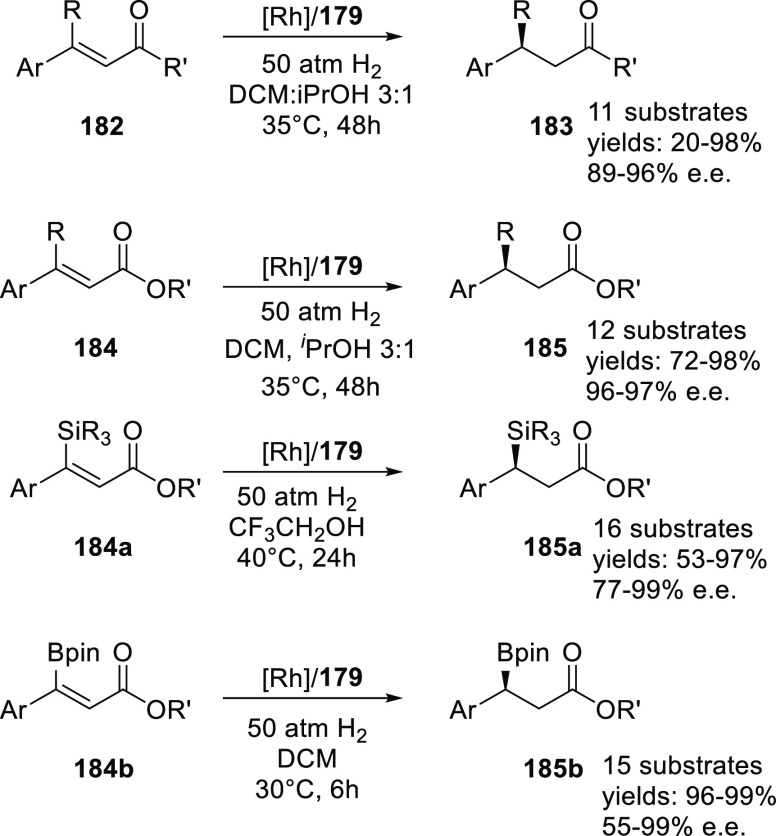
Part of the Extended Substrate Scope That Was Explored Using Zhaophos (**179**)

After the initial results using Zhaophos (**179**) in β,β-disubstituted nitroalkenes (**180**), the substrate scope for the rhodium catalyzed asymmetric hydrogenation was extended to challenging α,β-unsaturated ketones (**182**)^358^ and α,β-unsaturated esters (**184**)^359^ such as those shown in Scheme 40. These include substrates containing silicon (**184a**)^514^ and boron (**184b**)^515^ functional groups that provide handles for further functionalization. Also β-thio-α,β-unsaturated esters were converted with high selectivity using rhodium complexes based on Zhaophos (**179**).^360^

**Scheme 41:**
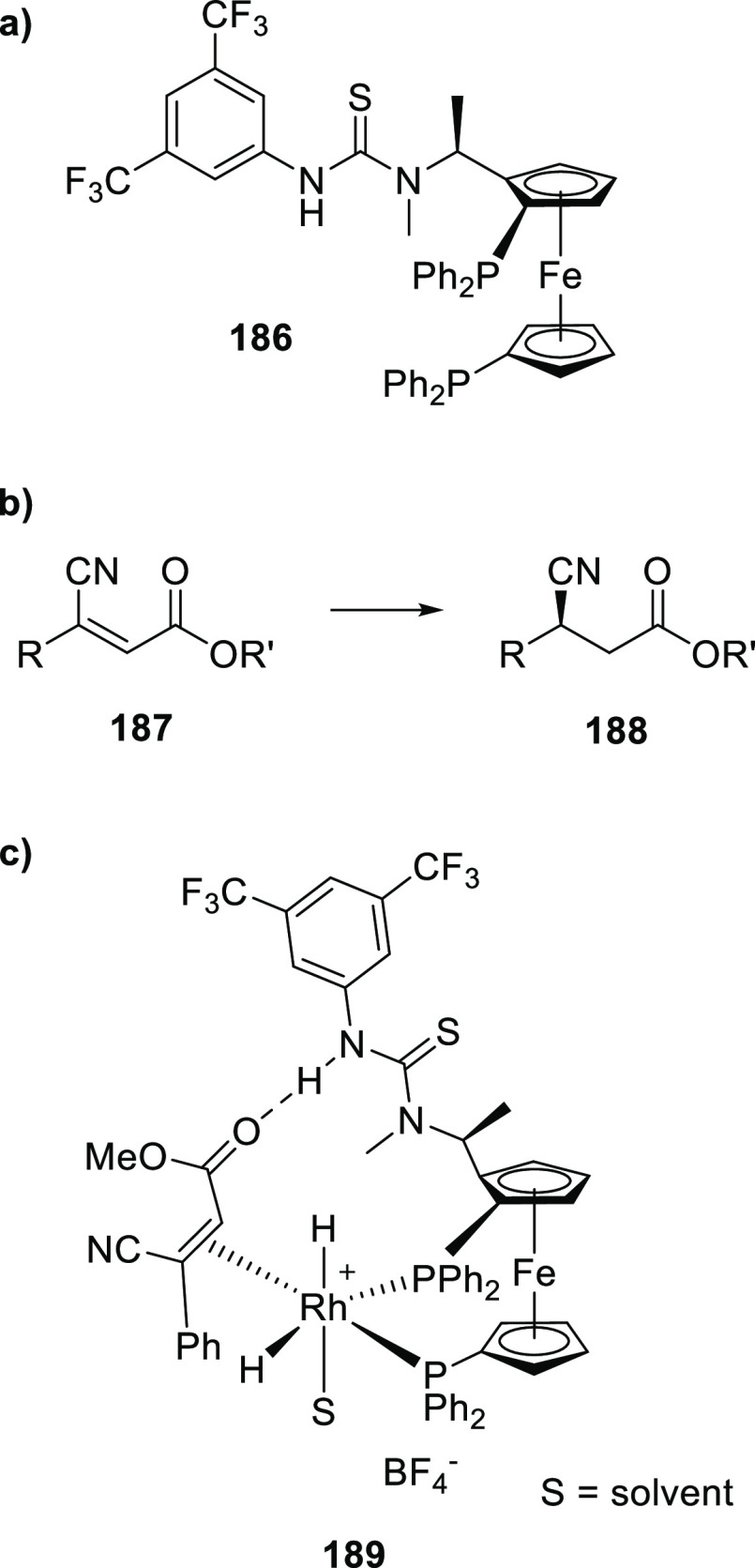
(a) NH-Methylated Analogue (**186**) of Zhaophos; (b) Asymmetric Hydrogenation of β-Cyano-α,β-unsaturated Esters (**187**) Using **186** to Yield the Product in 99% Selectivity; (c) Proposed Intermediate Structure (**189**) towards the Product

The asymmetric hydrogenation of β-cyano-α,β-unsaturated esters (**187**) was also explored using rhodium complexes based on Zhaophos. While the product was obtained already in high e.e., the selectivity could be further improved by using an analogue of Zhaophos (**186**) in which the least acidic NH of the thio-urea functional group was methylated (see Scheme 41).^357^ A Job plot analysis of a the binding study indicated that the substrate and ligand bind in a 1:1 ratio. DFT calculations suggest that the single HB formed between the substrate and the ligand leads to a stronger binding compared to that based on the two HBs formed between the substrate and the original Zhaophos (**179**). Also, the complex features a broad substrate scope of β-cyano-α,β-unsaturated esters, and the reaction was performed at gram scale to demonstrate the applicability.

**Scheme 42:**
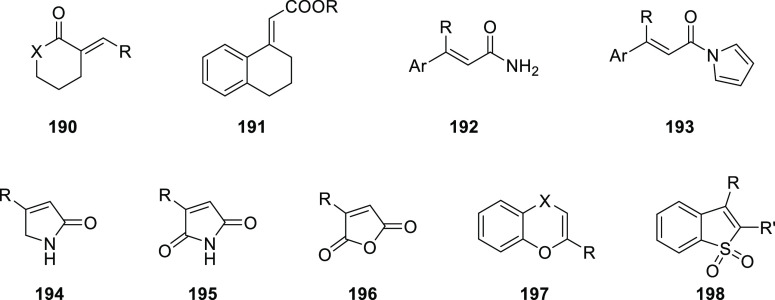
Further Extended Substrate Scope for Selective Rhodium Catalyzed Asymmetric Hydrogenation Using Zhaophos

The substrate scope for Zhaophos and analogues was further explored in the rhodium catalyzed asymmetric hydrogenation and appeared extremely versatile, as is summarized in Scheme 42. For many different subclasses of substrates, conditions were found in which the alkenes were hydrogenated in high e.e. (often >95% was reported), including exocyclic α,β-unsaturated lactones^361^ and lactams, (*E*)-2-chorman-4-ylidene)acetates,^362^ α,β-unsaturated primary amides,^359^ acylpyrazoles,^363^ five-membered α,β-unsaturated lactams,^364^ maleinimides, maleic anhydrides,^365^ 2-substituted benzo[*b*][1,4]dioxines, and benzo[*b*]thiophene 1,1,dioxides.^366^

**Scheme 43:**
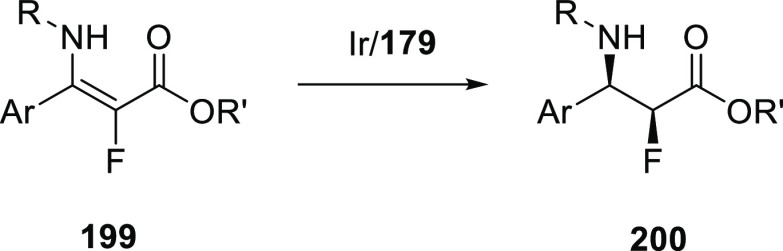
Iridium Catalyzed Asymmetric Hydrogenation Using Zhaophos (**179**)

The reaction scope was further extended to the iridium catalyzed asymmetric hydrogenation shown in Scheme 43. Various challenging tetrasubstituted α-fluoro-β-enamino esters were converted by the Ir/ZhaoPhos system, leading to products with two adjacent tertiary stereocenters. Excellent diastereoselectivities/enantioselectivities were reported (73%–99% yields, >25:1 diastereomeric ratio, 91%–>99% e.e.),^367^ with a very high activity using a relatively low catalyst loading (TON ≤ 8.600). Deuterium labeling studies suggest that the substrate does not isomerize to the imine before it is reduced. Also, different analogues of Zhaophos show low selectivity in this iridium catalyzed transformation, indicating also that for this system substrate orientation via the thiourea plays a crucial role.

**Scheme 44:**
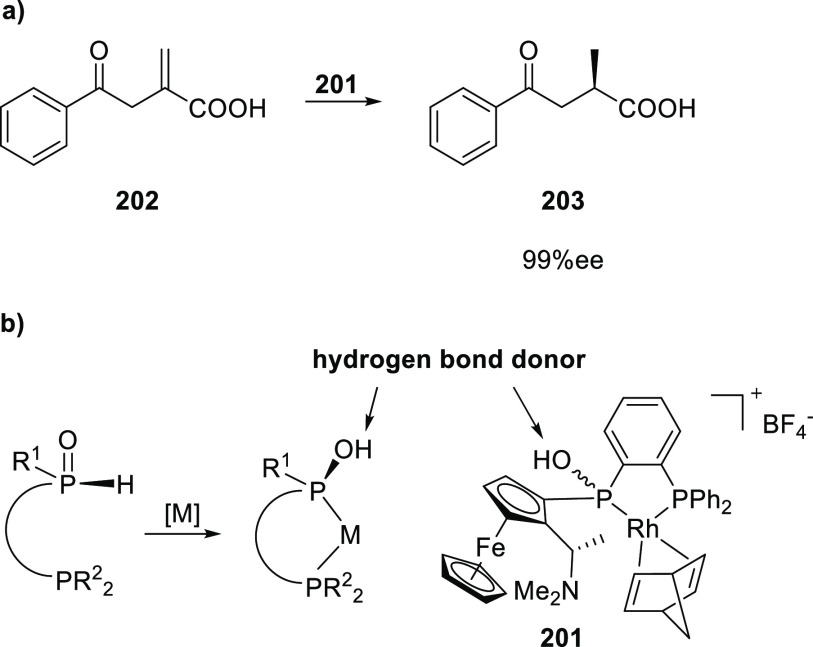
(a) Rhodium Catalyzed Asymmetric Hydrogenation Using SPO-Wudaphos (**201**); (b) Upon Coordination of SPO-Wudaphos (**201**), the P-OH Group Forms That Can Preorganize the Substrate by Hydrogen Bonding to the Ketone of **202** (Formation of a P–OH···O=C HB)

The Zhang group combined an SPO (see also section 3.1.1) with a normal phosphine in a bidentate ligand, coined SPO-Wudaphos (**201**), as illustrated in Scheme 44.^368^ When P coordinated to a metal, SPO-Wudaphos was tautomerized to its phosphinous acid and the resulting PO-H HB donor was demonstrated to give a HB interaction with substrates such as α-methylene-γ-keto carboxylic acids. Rhodium complexes based on the SPO-Wudaphos ligand converted this type of substrates with very high e.e. DFT calculations and control experiments demonstrated that the P-OH hydrogen bonded with the ketone, while the carboxylic acid function formed an amine salt ion pair with the amine function, and both were found to be important for the induction of high selectivity.

**Figure 9:**
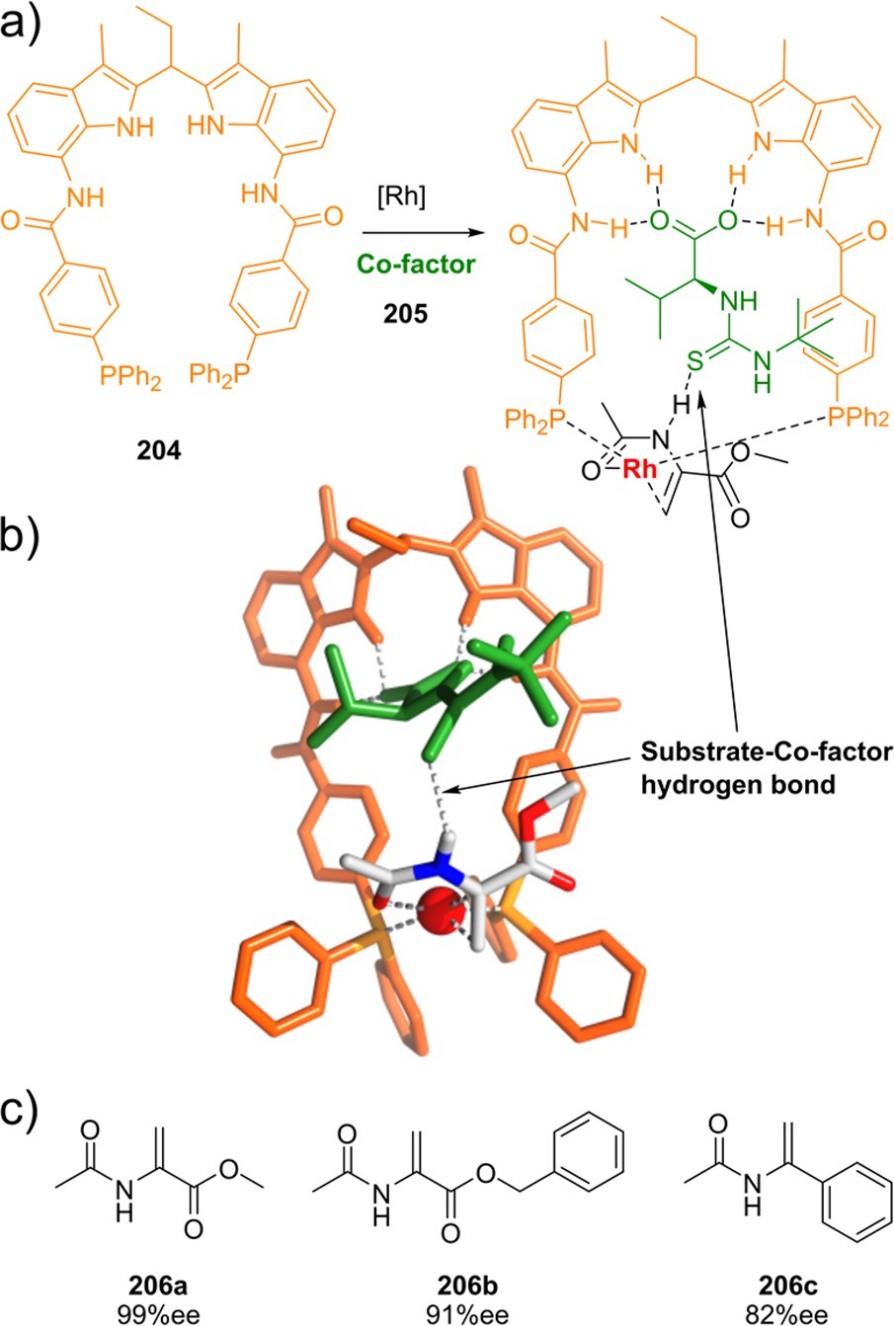
(a) Use of DIMPhos (**204**, in orange) with a chiral cofactor (green) for rhodium catalyzed hydrogenation. (b) Computed structure showing the HB established between the substrate and the bound cofactor, which is of crucial importance to obtain high selectivity in this reaction. (c) Three substrates that were converted with good to high enantioselectivity using the same cofactor. Adapted with permission from ref (346). Copyright 2014 RSC under a CC-BY-NC license [https://pubs.rsc.org/en/content/articlelanding/2014/SC/C3SC53505C].

Interestingly, the functional group for substrate orientation and the bidentate ligand do not necessarily need to be covalently linked, but similar cooperativity can also be generated via cofactor binding in the second coordination sphere. To demonstrate this concept, Reek and co-workers have explored a bidentate ligand with an integrated binding site for anions, coined DIMPhos (**204**) (see Figure 9).^369^ Chiral cofactors such as **205**, containing a carboxylate function, are bound to DIMPhos (**204**) rather strongly because of the formation of four HBs. The nonchiral bidentate DIMPhos (**204**) was explored in the rhodium catalyzed asymmetric hydrogenation of methyl 2-acetamidoacrylate (**206a**) in the presence of various chiral cofactors. Importantly, under these conditions the cofactor was the only source of chirality. From the 18 cofactors evaluated, one provided a supramolecular complex that converted methyl-2-acetamido-acrylate in very high enantioselectivity (99% e.e.), whereas other cofactors resulted in low to moderate selectivity (≤61% e.e.). Two other amidoacrylates (**206b** and **206c**) were converted with high enantioselectivity using the same cofactor. Control experiments and DFT calculations suggest that also for this catalyst a HB between the substrate that is coordinated to the rhodium metal center and the cofactor is of crucial importance. Interestingly, similar to the Zhang system (Figure 8), a thiourea-containing cofactor appeared to be important for selectivity, but in this system the HB is formed between the sulfur and the amide NH of the substrate, rather than between the urea NH and the carbonyl of the substrate (Figure 9). The substrate scope was not extensively explored, but considering the results of Zhaophos, it could well be that this system is also widely applicable. Importantly, the above systems all show that hydrogen bonding between the substrate and the catalyst in the second coordination sphere, next to substrate coordination, can play an important role in achieving highly selective catalysis. Thus, hydrogen bonding is a powerful tool to guide the rational design of hydrogenation catalysts.

### 4.2 Hydroformylation Catalysis

**Figure 10:**
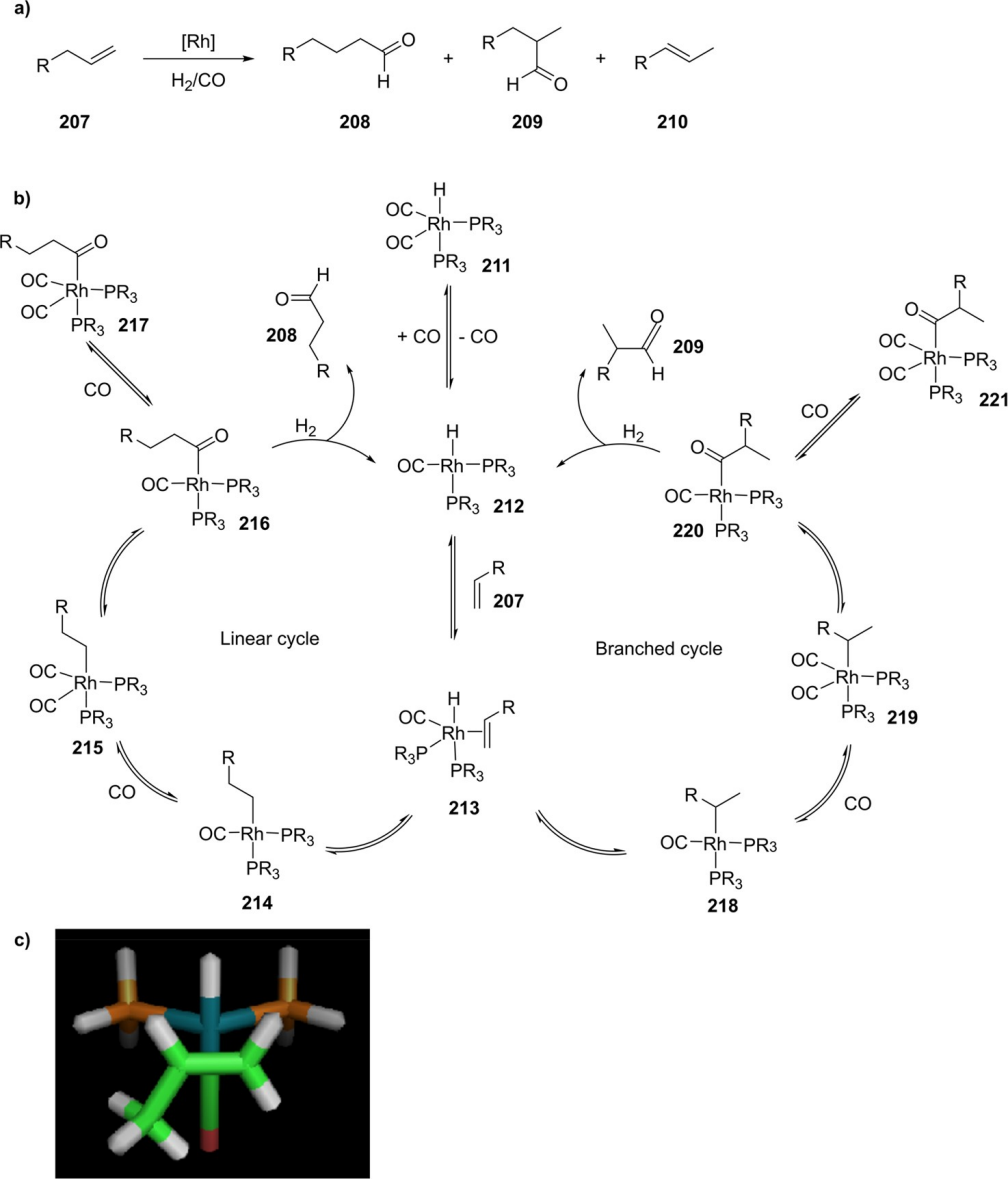
(a) Hydroformylation reaction in which alkene groups are converted to aldehyde products by the addition of CO and H_2_; (b) Generally accepted mechanism, proceeding via alkene coordination (**212** to form **213**), hydride migrations (**213** to form **214**/**218**), CO coordination and insertion (**214**/**218** to form **216**/**220**), and hydrogenolysis (via oxidative addition/reductive elimination or metathesis; **216**/**220** to form **222**); (c) molecular model of the selectivity determining hydride species (**213**) to illustrate that hydrogen bonding can control this step by preventing substrate rotation (clockwise or counterclockwise), thus determining the reaction pathway (to **214** or **218** resulting in linear or branched products, respectively).

The hydroformylation reaction typically involves a metal catalyzed addition of CO and H_2_ to an alkene as is shown in Figure 10a. This reaction was discovered by serendipity in the thirties of the last century and has further resulted in many industrial applications.^370,371^ Fundamental studies have provided detailed insight in the reaction mechanism,^372,373^ and many issues regarding selectivity and activity have been solved. Yet, there are still several challenges left that, when successfully sorted out, can lead to new industrial applications. These challenges mainly involve selectivity issues, including the branched selective hydroformylation, the selective hydroformylation of internal alkenes, and the selective hydroformylation of tri- and tetrasubstituted alkenes. Also, the asymmetric hydroformylation of terminal disubstituted alkenes is a largely unsolved problem. Whereas the typical approach to control activity and selectivity in rhodium catalyzed hydroformylation involves changes in the ligand structure (electronic, steric, and the bite angle), more recently it has been demonstrated by the groups of Reek^374^ and Breit^375^ that hydrogen bonding between the substrate and the catalyst in the second coordination sphere can also be used to obtain selective hydroformylation catalysts.

The generally accepted mechanism for rhodium complexes with phosphorus ligands is displayed in Figure 10b.^372,373^ For complexes based on phosphine ligands, the resting state is usually the rhodium(I) hydrido complex (**211**). The cycle starts with CO dissociation and subsequent alkene coordination to form the alkene–rhodium hydride complex (**213**), of which a model is presented in Figure 10c. The hydride migration step that follows to either C2 (to give **214**) or C1 (to give **218**) leads the path to form the linear or the branched product, respectively. During this selectivity determining migration step, the alkene rotates clockwise or anticlockwise, depending on the carbon atom to which the hydride migrates. As such, fixation of the substrate by hydrogen bonding in the second coordination sphere may block some of these rotations and can thus lead to more selective hydroformylation catalysis.

**Scheme 45:**
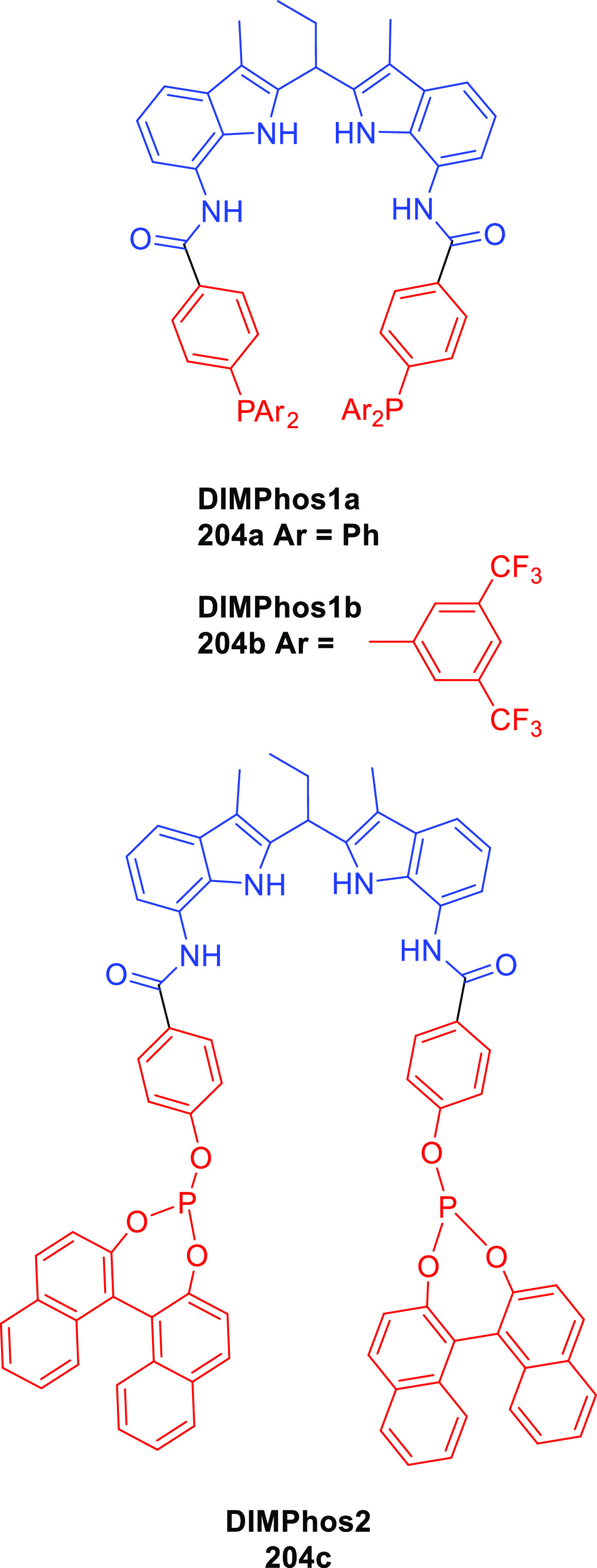
Three DIMPhos Ligands with Aryl Phosphines (DIMPhos1, **204a** and **204b**) and with Phosphites (DIMPhos2, **204c**) with the Binding Site Displayed in Blue and the Ligand in Red

Reek and co-workers employed bidentate DIMPhos ligands (**204a**–**c**) such as those shown in Scheme 45 for the selective rhodium catalyzed hydroformylation of alkene substrates containing carboxylate or phosphate functional groups.^374^ These ligands have an integrated HB binding site for carboxylate (and phosphate) functional groups (in blue) that can be used to orient a substrate containing these groups. Under hydroformylation conditions, the ligands bind to a rhodium center in a bidentate fashion forming the typical rhodium-hydride bis-carbonyl complexes that are active in hydroformylation. Binding studies monitored by IR and NMR spectroscopy showed that the metal complexes strongly bind to acetate groups in the NH-rich binding pocket of DIMPhos (blue), while leaving the coordination geometry around the metal unaffected. Exploration of a series of substrates with carboxylate functional groups in hydroformylation catalysis demonstrated that substrate preorganization results in unprecedented selectivities. A broad range of terminal and internal alkenes functionalized with an anionic carboxylate (or phosphate) group has been used.

**Figure 11:**
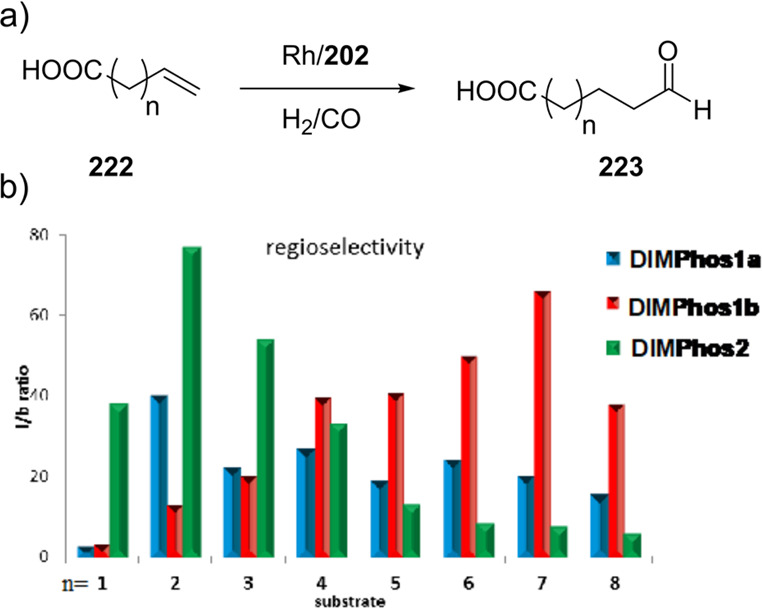
Selective hydroformylation of terminal alkenes (**222**) to linear aldehydes (**223**) using various DIMPhos ligands (**204a**–**c**) that can form HBs with the substrate. The l:b ratio as a function of the number of carbon atoms (*n*) is displayed.

The three DIMPhos ligands (**204a**–**c**) shown in Scheme 45 were used in the rhodium catalyzed hydroformylation of terminal unsaturated carboxylates. As is summarized in Figure 11, 4-pentenoate up to 10-undecenoate are converted to the aldehyde with high selectivities for the linear product when DIMPhos1a is used (**204a**). The methyl ester analogues of these substrates, which have no significant affinity to the binding pocket, are converted with low selectivity, confirming the need for HB formation. Detailed kinetic analysis shows that these complexes convert carboxylate substrates via a mechanism that follows Michaelis–Menten kinetics, with product inhibition.^376^ The Michaelis–Menten constant is the same as the product inhibition constant, reflecting the carboxylate binding in the DIMPhos pocket. The authors concluded from detailed experiments that first the substrate is bound in the pocket, experiencing competition from the product, and then the alkene is converted to the aldehyde. Importantly, although the mechanism is described with a model that includes product inhibition, the reaction rates are high (and accelerated by binding) and full conversion can easily be reached. The use of DIMPhos1b (**204b**) also gave high selectivities for the larger substrates, but the trend could not be clearly explained. The use of the phosphite analogue DIMPhos2 (**204c**) also resulted in the formation of the 1-aldehyde in high selectivity, also for the smallest substrate (*n* = 1). Whereas the distance between the alkene and the carboxylate in this substrate is too small to simultaneously coordinate to rhodium with the alkene and to the DIM pocket with the carboxylate in complexes formed by DIMPhos1a (**204a**), the extra flexibility in the phosphite analogue allows this.

**Figure 12:**
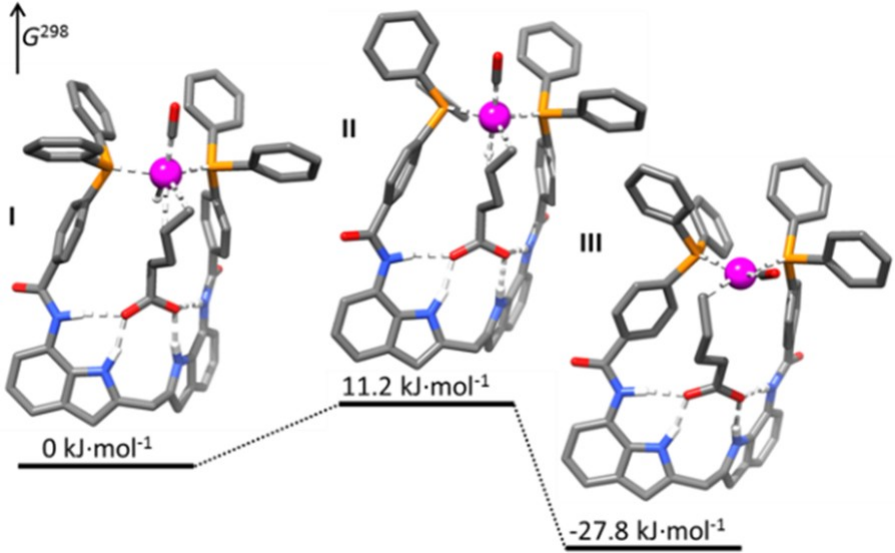
DFT calculated structures of the hydride migration that leads to the linear aldehyde. The pathway to the branched product would require the breakage of the HBs or proceed via other intermediates that are even higher in energy (not displayed).

The concept of substrate orientation by hydrogen bonding was further supported by DFT calculations on the hydride migration step as is illustrated in Figure 12.^374−376^ Only the carbon atom close to the hydride is available for the migration because of the carboxylate binding in the pocket, and in the precomplex, the alkene is already rotated toward the transition state of hydride migration. The hydride migration that leads to the intermediate that forms the branched aldehyde requires the alkene to rotate clockwise (about 180°). This is, however, not possible without breaking the HBs between the substrate and the HB pocket of DIMPhos. From these calculations it is also clear that 3-butenoate (*n* = 1 in Figure 11) is too short to bind simultaneously to the receptor moiety and the metal center in complexes based on DIMPhos1a (**204a**).

**Figure 13:**
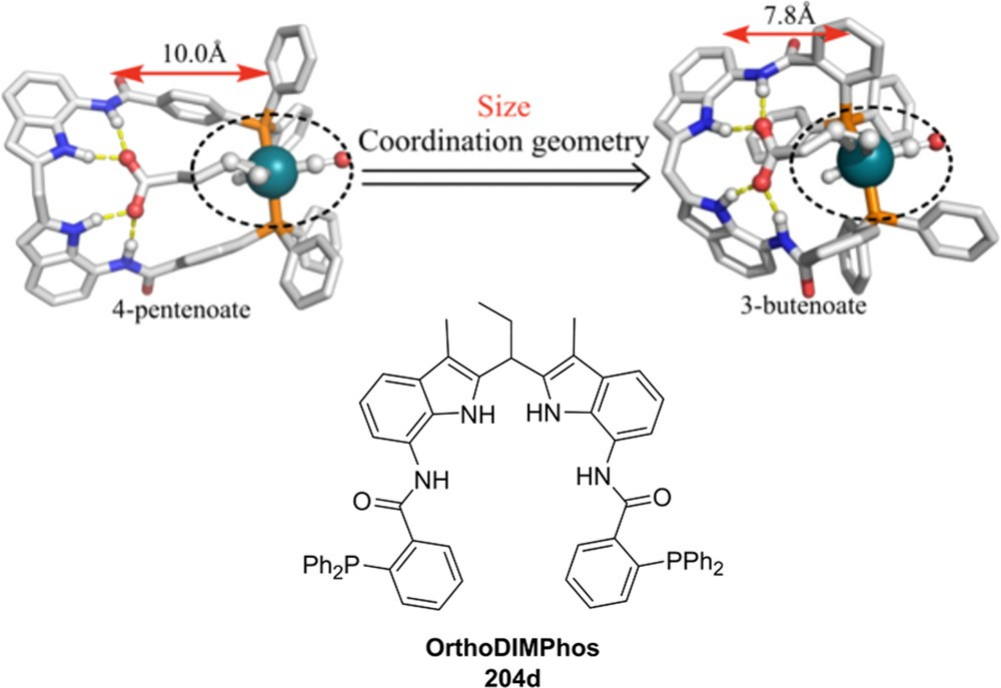
Redesign of DIMPhos1a (**204a**) to provide OrthoDIMPhos (**204d**), an analogue with a shorter distance between the Rh-complex and the DIM pocket. Adapted with permission from ref (377). Copyright 2019 Wiley under a CC-BY-NC license [https://chemistry-europe.onlinelibrary.wiley.com/doi/10.1002/cctc.201900487].

In order to also address the small 3-butenoate substrate with phosphine-based ligands, DIMPhos1a (**204a**) was redesigned by repositioning the -PPh_2_ moieties from the *para* position to the *ortho* position in OrthoDIMPhos (**204d**). The DFT calculated structures displayed in Figure 13 show that this alteration shortens the distance between the HB binding site and the rhodium complex from 10 to 7.8 Å, the ideal distance to ditopically bind 3-butenoate. Application of this ligand in the rhodium catalyzed hydroformylation of 3-butenoate indeed demonstrated selectivity to the linear product in record high selectivity (l/b = 84).^377^

**Scheme 46:**
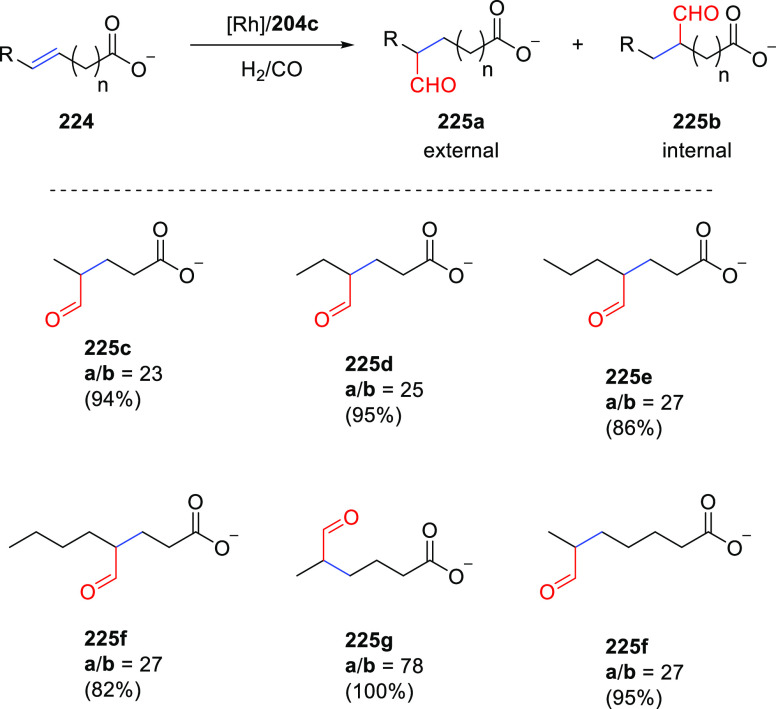
Selective Hydroformylation of Internal Alkenes Using Rhodium Complexes Based on DIMPhos2 (**204c**) with the Indicated Quotients of External (a)/Internal (b) Aldehydes

The concept of supramolecular substrate orientation by hydrogen bonding was also explored for internal alkenes. These substrates are less reactive, and the selectivity is harder to control, as there is generally no electronic bias with the consequence that the two aldehydes are typically produced in a ratio close to 1:1. Rhodium complexes based on the phosphine-based ligand DIMPhos1a (**204a**) are too unreactive to convert internal alkenes under mild conditions. In contrast, rhodium complexes based on a phosphite ligand are generally more reactive.^373^ Indeed, deploying DIMPhos2 (**204c**) to support a Rh complex led to conversion of internal alkenes under mild conditions.^376^ The application of a rhodium complex based on DIMPhos2 (**204c**) on the series of carboxylate functionalized substrates listed in Scheme 46 showed that these were converted to aldehydes with very high selectivity. In the products that are formed, CO is inserted in the carbon atom furthest away from the carboxylate, in line with the selectivity obtained for terminal alkenes. For some substrates, exceptionally high selectivities are observed with a quotient of external/internal aldehyde of 78. Substrates with different distances between the alkene and the carboxylate were selectively converted with the highest selectivity obtained for the internal alkene on the 4-position.

**Scheme 47:**
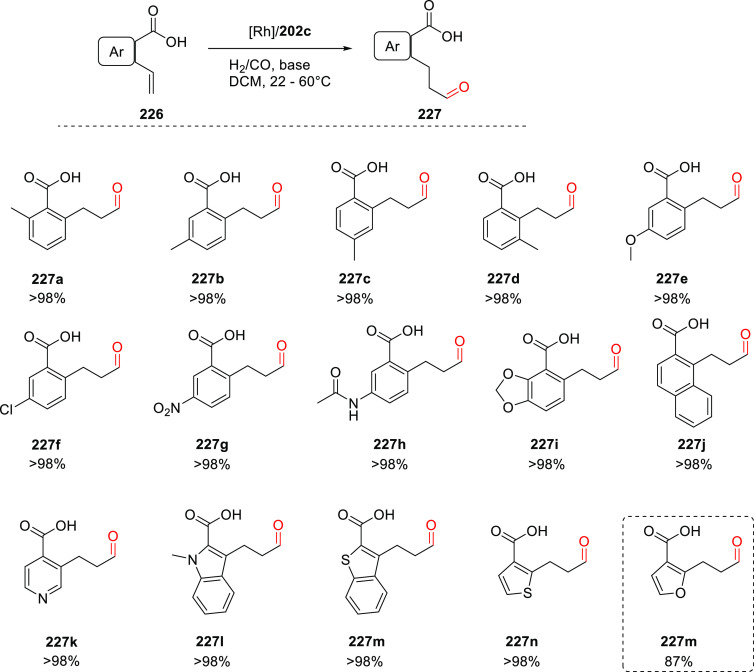
Styrene Derivatives That Have Been Converted with High Selectivity Using DIMPhos2

Using the same ligand, it was demonstrated that even reversal of selectivity can be obtained by organization of the substrate by hydrogen bonding. The linear aldehyde is usually the disfavored product in the hydroformylation of vinyl 2- and 3-carboxyarenes, but by application of DIMPhos2 (**204c**), chemo- and regioselectivities up to 100% can be achieved, as summarized in Scheme 47. The catalyst proved to be selective for a wide scope of substrates, could be applied at low catalyst loading, and worked well at ambient pressure.^378−380^

The catalyst was still selective at temperatures up to 120 °C, and very high reaction rates were observed at these temperatures. Follow-up reactions on the formed products demonstrated the wide possible applicability, paving the way to designing new synthetic routes for biorelevant compounds. Also, the most challenging substrates with internal double bonds, such as methylstyrene derivatives and the cyclic analogues thereof, were converted with exceptional selectivity. Kinetic studies and *in situ* spectroscopy revealed that the active species involve complex equilibria including dormant species. The reaction kinetics is described by a model including both product inhibition and substrate inhibition due to binding of the carboxylate to the binding site and to the metal center. Nonetheless, efficient formation of the desired product is observed with TOF’s as high as 2000 mol_substrate_/mol_catalyst_·h^–1^.

**Figure 14:**
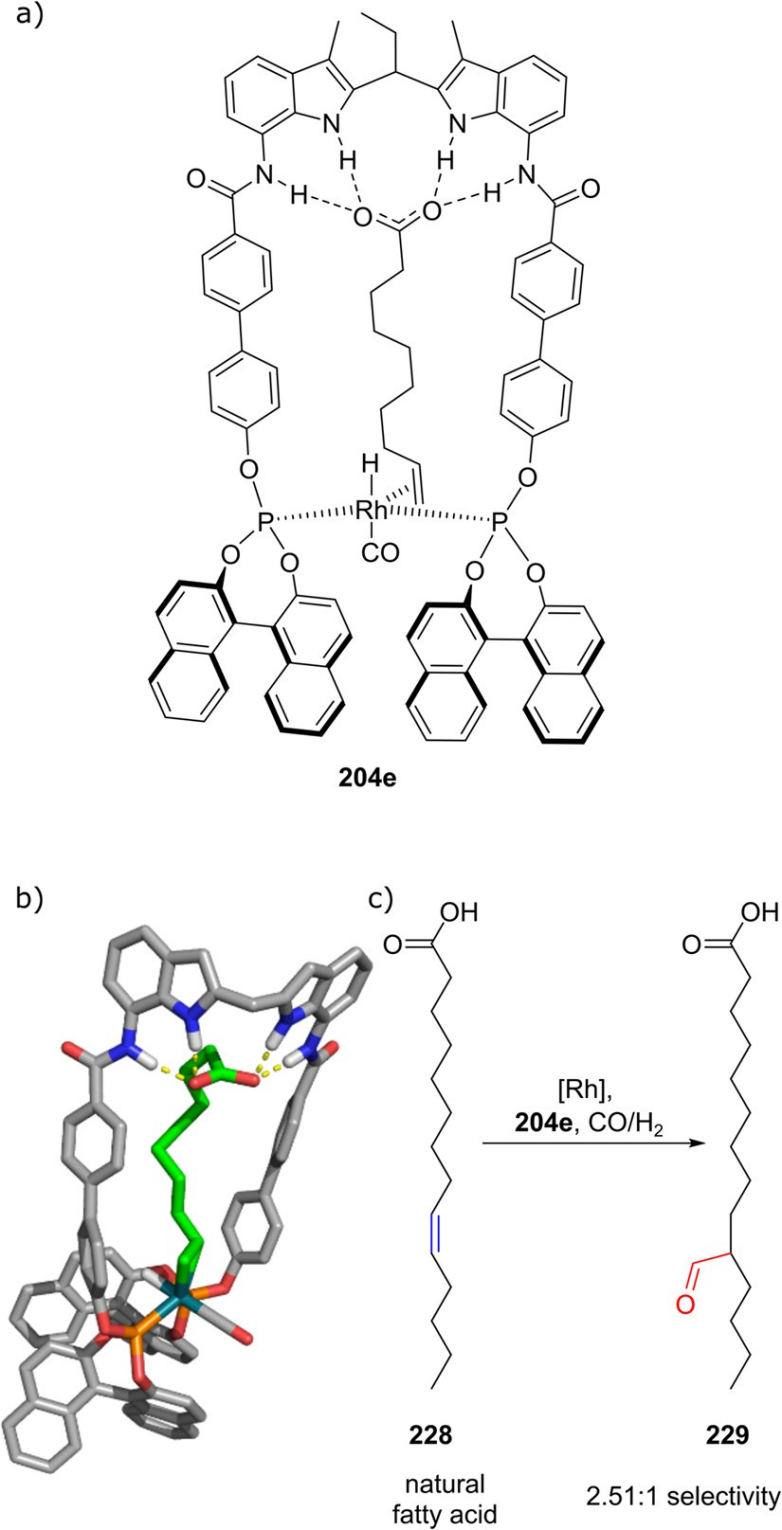
(a) Extended DIMPhos ligand (**204e**) for substrate preorganization by hydrogen bonding of larger substrates; (b) modeling picture showing the binding of 9-decanoate as a model for natural fatty acids such as **228**. (c) **228** is selectively converted to aldehydes such as **229**. Adapted with permission from ref (381). Copyright 2020 Wiley.

The hydroformylation of natural monounsaturated fatty acids (MUFAs) in a regioselective fashion requires a catalyst with a larger distance between the rhodium center and the HB binding pocket of the DIMPhos ligand. With this in mind, the phenyl “linker” between the HB binding pocket and the -PPh_2_ moieties was extended to a biphenyl in **204e**, as shown in Figure 14a.^381^ DFT calculations suggest that 9-decanoate spans the distance between the HB binding pocket and the rhodium complex reasonably well, making it a reasonable model for fatty acids. The hydroformylation catalyst based on the extended DIMPhos ligand (**204e**) converts substrates with high regioselectivity, including monounsaturated fatty acids (MUFAs) and their model substrates. For example, as shown in Figure 14b, the natural fatty acid *cis*-myristoleic acid could selectively be hydroformylated with a 10-formyl/9-formyl ratio of 2.51. This, in fact, is the first selective hydroformylation catalyst for this biobased compound.

The results obtained with different versions of the DIMPhos ligand demonstrate that substrate preorganization via hydrogen bonding allows the redesign of catalysts to provide selective conversions for substrates of different sizes.

**Figure 15:**
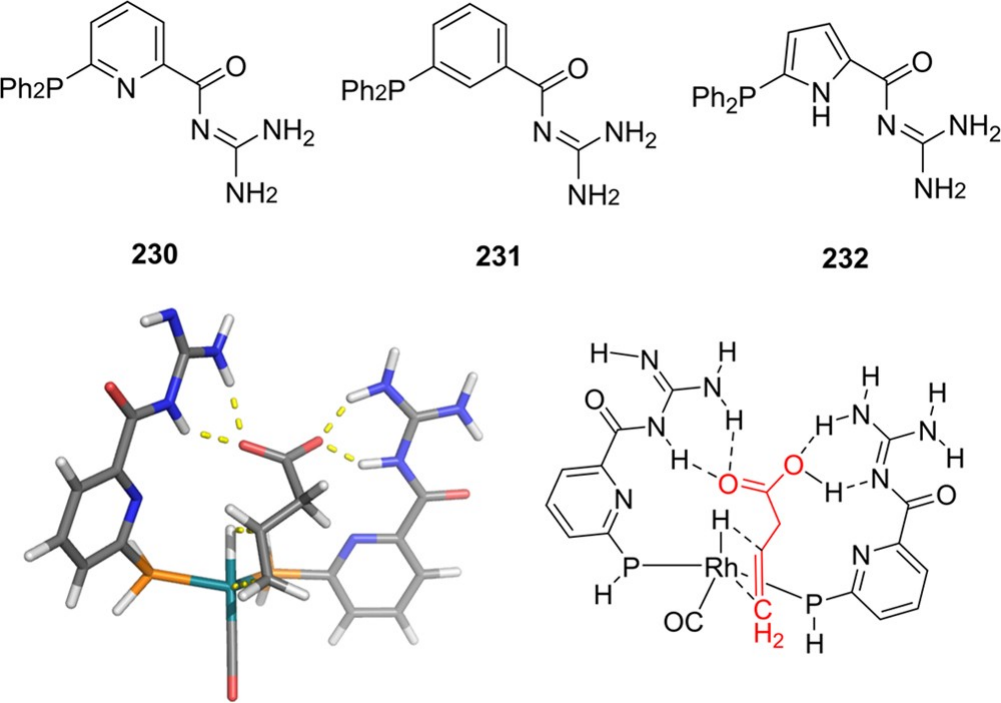
Acyl guanidine functionalized phosphine ligands (**230**–**232**) which form HBs to carboxylic acid functionalized substrates. The DFT calculated structure based on **230** (with *in silico* PPh_2_ to PH_2_ mutation) shows that the substrate is preorganized at the rhodium complex by hydrogen bonding, to control the subsequent selectivity determining hydride migration step (see also Figure 10b).

Substrate orientation can also be achieved by using monodentate ligands with functional groups. Breit and co-workers explored the use of the acyl guanidinium functionalized phosphine ligands (**230**–**232**) shown in Figure 15, in which the guanidine can form HBs to unsaturated carboxylic acids.^375^

These HBs between substrate and ligand lead to substrate preorganization of the alkene at the metal center. 3-Butenoic acid is converted by a rhodium catalyst based on ligand **230** with a high selectivity for the linear product (l:b = 41 under optimized conditions). In experiments in which competitive guests (with carboxylic acid functional groups) are present, the substrates are converted with lower selectivity and activity in line with the substrate preorganization model. As is exemplified for 3-butenoic acid in the bottom of Figure 15, DFT calculations show the Rh(H)CO–substrate complex is most stable when two P ligands are coordinated to Rh and the carboxylic acid moiety of the substrate forms HBs with the guanidines. In this example, one guanidine deprotonates the acid to give a carboxylate that can form four HBs, very similar to the case observed for DIMPhos. No substrate–ligand interaction was observed in complexes with only one ligand coordinated to the metal center, and as such, the bis-coordinated species is proposed to be the most likely intermediate responsible for the high selectivity.^382^ Analysis of the calculated structures indicates that preceding the hydride migration step, the alkene is already rotated toward the hydride as a result of constraints imposed by the HBs between the guanidinium moieties and carboxylic acid moiety of the substrate.

The catalyst also converts internal alkenes such as 3-pentenoic acid with high selectivity for aldehyde introduction on the unsaturated carbon atom furthest away from the carboxylic acid (with ratio 18:1). The selectivity was found to be highly dependent on the distance between the acid moiety and the alkene function. 4-Pentenoic acid was converted with comparable selectivity to levels typically found for triphenyl phosphine-based catalysts. This clearly shows that for this catalyst system the alkene–acid distance must be precise in order to control selectivity through hydrogen bonding. The high selectivity for the 3-pentenoic acid was only obtained for the *cis* configurated alkene. In a following paper^383^ the authors demonstrated that the use of an electron-poor acylguanidine ligand (**252**) provided complexes that could also convert β-alkynoic acids to provide similar products.

**Scheme 48:**
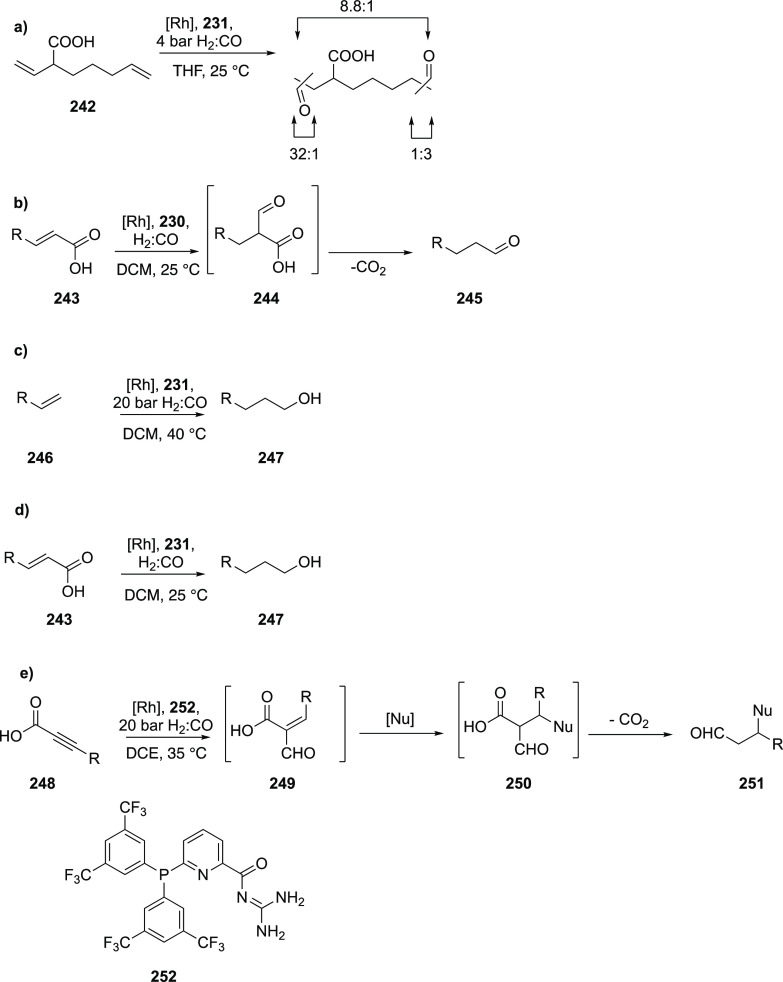
(a) Site-Selective Hydroformylation by Substrate Organization via Hydrogen Bonding Containing Multiple Olefinic Sites, (b) Decarboxylative Hydroformylation of *α,β* Unsaturated Carboxylic Acids by Substrate Organization, (c) Tandem Hydroformylation–Hydrogenation Sequence Converting 1-Octene into 1-Nonanol, (d) Tandem Decarboxylative Hydroformylation–Hydrogenation of α,β Unsaturated Carboxylic Acids, and (e) Tandem Supramolecular Rh Catalyzed Hydroformylation of α-Alkynoic Acids Followed by Michael Addition and Decarboxylation

This substrate selectivity can actually be exploited for substrates containing two alkenes at different distances from the carboxylate, as shown in Scheme 48a. As the alkene closer to the carboxylic acid better matches the guanidine–rhodium distance, this alkene is converted at a higher rate with a ratio of 8.8:1. In addition, this alkene is converted with higher selectivity for the linear aldehyde with l:b = 32, compared to an l:b ratio of merely 0.3 for the other alkene moiety.^382^

Next to regular hydroformylation, also decarboxylative hydroformylation of α,β-unsaturated carboxylic acids was explored (see Scheme 48b).^384^ Using substrate preorganization, the alkene was selectively functionalized, leading to the aldehyde intermediate, which after decarboxylation, results in the final linear aldehyde product. Complexes based on triphenylphosphine did not lead to this linear aldehyde but instead only gave reduction of the double bond.

Modifications on the ligand building blocks were explored. When instead of the pyridine-containing ligand (**230**), the benzene (**231**) or a pyrrole (**232**) analogue was used as ligand (see Figure 15), aldehyde hydrogenation is observed.^385^ Complexes based on **231** and **232** were thus used in a tandem hydroformylation–hydrogenation sequence converting 1-octene into 1-nonanol (Scheme 48c). The selectivity for the linear alcohol can be enhanced by using a catalyst based on the pyrrole analogue of the guanidium ligand, in combination with the 2-pyridone/2-hydroxypyridine hydrogen-bonded bidentate (6-DPPon (**135**), Scheme 29). This indeed provided a highly selective hydroformylation–hydrogenation reaction of 1-octene to 1-nonanol. Furthermore, combining the decarboxylative hydroformylation catalyst with a supramolecular aldehyde hydrogenation catalyst yields an effective system for tandem decarboxylative hydroformylation–hydrogenation (Scheme 48d).^386^ The yield for the alcohol can be 99% when **230** is used or when a mixture of ligands is used. Finally, the supramolecular approach was used for the Rh catalyzed hydroformylation of α-alkynoic acids followed by Michael addition and decarboxylation, using an electron-poor phosphorus ligand equipped with the acyl guanidine moiety (**252**) (see Scheme 48e).^387^ This domino reaction is triggered by the Rh catalyzed hydroformylation of α-alkynoic acids, requiring the hydrogen bonding interaction between the ligand and the substrate. Consecutive Michael addition of arenes as nucleophiles lead to an intermediate which after decarboxylation of the carboxyl function leads to the β-aryl aldehyde products. In this sequence the carboxyl function is a transient and traceless directing group for the introduction of the aldehyde function. The strategy has been used for the preparation of a key intermediate for the synthesis of Avitriptan.

### 4.3 C–H Activation

Transition metal catalyzed C–H bond activation allows the introduction of functional groups at a late stage of a synthesis protocol and, as such, is an increasingly applied tool.^388−394^ The direct C–H borylation is of particular interest as it installs a boron functional group, which allows further modification by, for example, Suzuki coupling reactions, amination, hydroxylation, and halogenation.^395−399^ Controlling the selectivity of a C–H activation reaction is particularly challenging, as typically there are several similar C–H bonds present in a molecule and they are not electronically activated. Recently, the use of hydrogen bonding between the substrate and the ligand of a metal complex has been explored to control the selectivity. These approaches resulted in regioselective catalysts for C–H borylation for a diversity of substrates.

**Figure 16:**
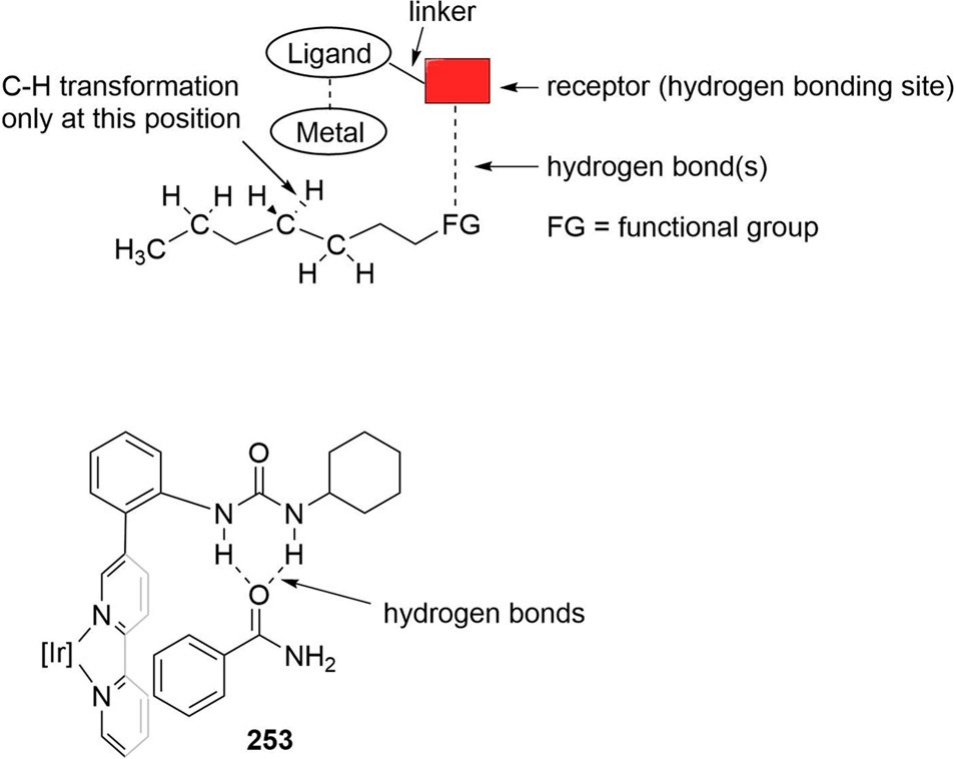
(a) Concept of HB directed selective C–H activation and (b) urea functionalized bipy ligand that forms an iridium complex that displays regioselectivity in C–H borylation reactions.

**Scheme 49:**
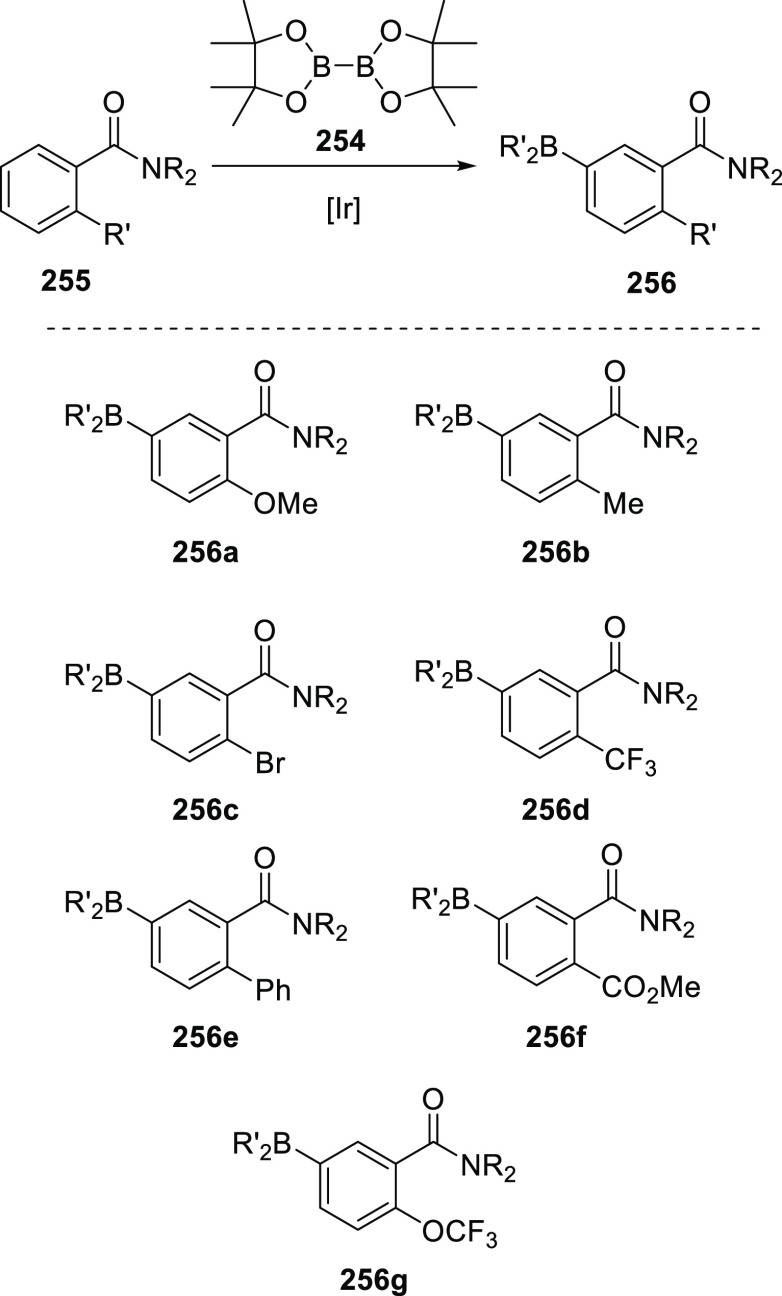
HB Directed *meta*-Selective C–H Activation Using the Urea Functionalized Ligand (**253**) (See Figure 16) Part of the substrate scope is displayed with *meta*/*ortho* > 7.

The hydrogen bonding approach was pioneered by Kanai and co-workers, who explored the use of urea functionalized 2,2′-bipyridine (bpy) ligands exemplified in Figure 16.^400^^1^H NMR spectroscopic experiments established that HBs form between the amide functional groups of the substrate and the urea motifs. In the hydrogen-bonded complex, the substrate is preorganized for *meta*-selective C–H activation. Application of *N,N*-dihexylbenzylamide as a substrate in the iridium catalyzed borylation afforded the *meta* product in high selectivity (*meta*/*para* = 8.3 under standard conditions, 27 in *p*-xylene as solvent). Importantly, control experiments in which the urea functional groups were not properly positioned resulted in unselective borylation reactions. The thiourea analogue was also used; however, the iridium complex of this ligand did not provide any product. It was also shown that the hydrogen bonding resulted in faster catalysis and further optimization of the ligand was possible by the presence of functional groups at the bipy part of the ligand.^401^ The approach was followed up by Phipps and co-workers, who used an iridium catalyst containing sulfonated anionic bpy ligands that were active for *meta*-selective borylation.^402^ In these systems HBs were proposed to be formed with the anionic sulfonate group. Using a similar preorganization strategy as reported by Kanai, Chattopadhyay, and co-workers made a bpy ligand functionalized with a naftapyrildone functional group, which also gave highly *meta*-selective borylation catalysts.^403,404^ Part of the substrate scope that could be achieved with urea-functionalized ligands is shown in Scheme 49, demonstrating that the reaction can tolerate a variety of different functional groups.

**Figure 17:**
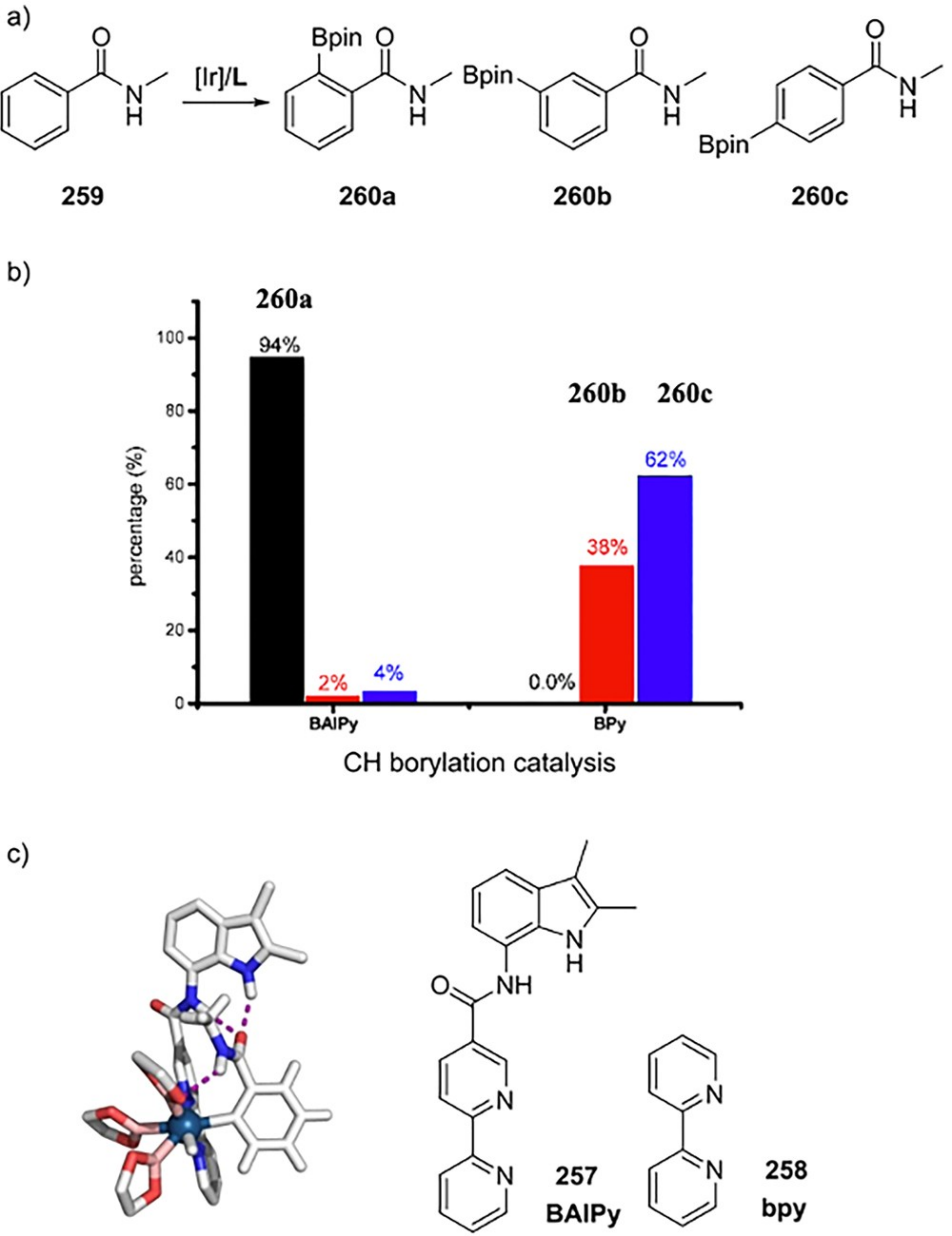
(a) Ir catalyzed C–H borylation of **259** to **260**; (b) selectivity patterns obtained using Ir catalysts based on BAIPy (**257**) or bpy (**258**). (c) DFT modeling of the substrate–BAIPy (**257**)–trisboryl–Ir catalyst complex shows that three HBs form between the substrate and the catalyst (purple dashed lines). The indole amide functional group in **257** thus orients the substrate by hydrogen bonding with secondary aromatic amide substrates, leading to *ortho*-selective C–H borylation. The model reaction using complexes based on bipyridine as the ligand (bpy, **258**) does not display this selectivity. Adapted with permission from ref (405). Copyright 2019 Wiley under a CC-BY-NC license [https://onlinelibrary.wiley.com/doi/10.1002/anie.201907366].

In order to have access to *ortho*-borylated compounds (**256**), a new ligand was designed that also operates via HB-assisted substrate orientation. A bipyridine ligand was functionalized with an indole amide functional group, coined BAIPy (**257**) (Figure 17c), which has the HB donors closer to the metal in the iridium complex.^405^ DFT calculations of the C–H activation step show that three HBs form between the substrate and the catalyst. The two anticipated HBs form between the indole amide and the carbonyl of the substrate, while a third unexpected HB was identified between the N–H moiety of the substrate and an oxygen of a Bpin group attached to iridium. Experiments employing the model substrate show high selectivity for *ortho*-selective C–H borylation. In contrast, in the control experiment with the parent bis-pyridyl ligated iridium complex, only *meta* and *para* borylated product are formed, and the *ortho* borylated product is not formed at all (Figure 17a and b). Interestingly, using *N,N*-dimethylbenzylamide as substrate gave substantially lower *ortho* selectivity (1:1), indicating that the ^substrate^N–H···O^Bpin^ HB identified in the complex with *N*-methylbenzylamide (Figure 17c) is of importance.

**Scheme 50:**
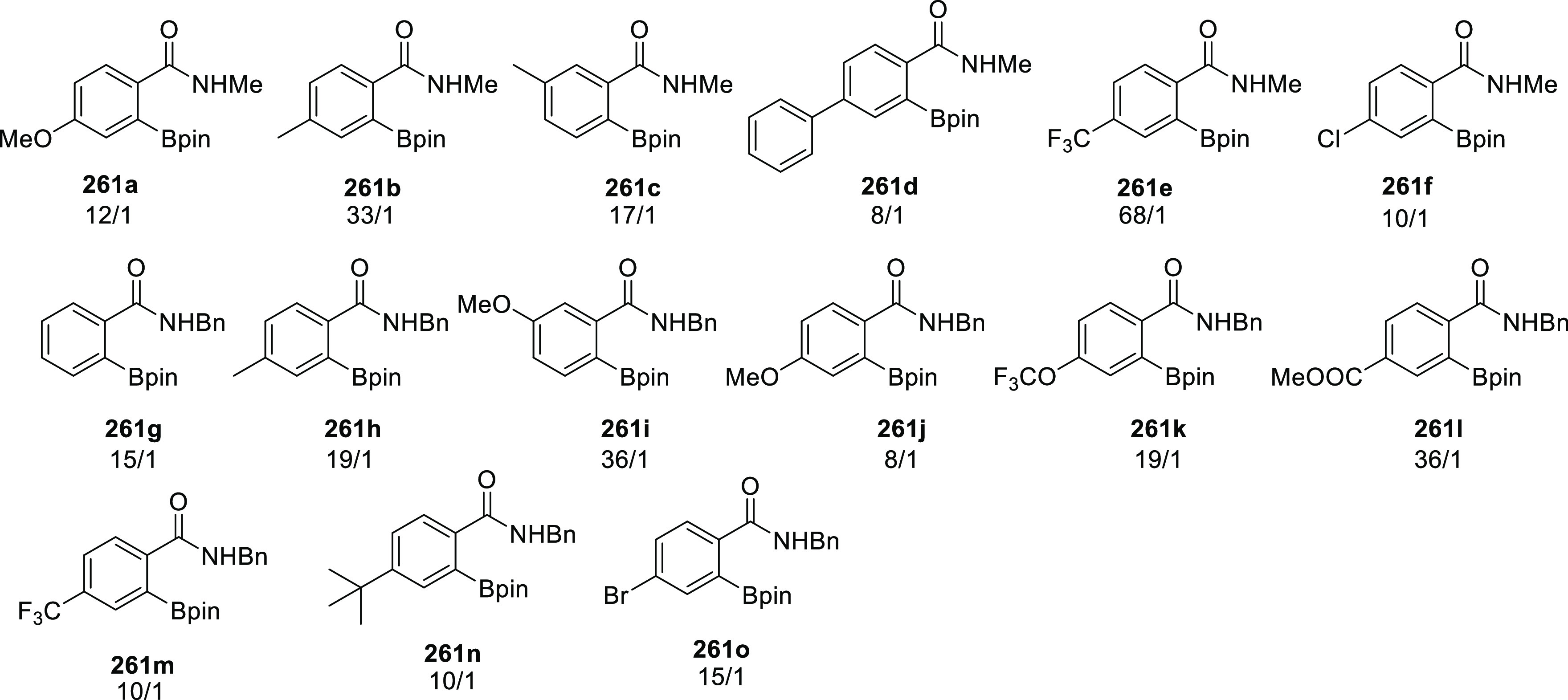
Part of the Substrate Scope of Selective Borylation Using Iridium Complexes of BAIPy (**257**)

In addition to the high selectivity achieved, hydrogen bonding between the substrate and ligand also enabled faster catalytic reactions. In the substrate scope illustrated in Scheme 50, more than 26 examples of *N*-methylbenzamides and aromatic amides were reported, including peptide-based analogues. This large substrate variety demonstrates that this supramolecular catalyst is compatible with a plethora of functional groups, featuring the catalyst’s general applicability. The supramolecular iridium catalyst has been applied at gram scale with high conversion and selectivity at elevated temperature. The ligand is easily prepared at large scale, facilitating the application of catalysts that operate via hydrogen bonding in C–H borylation. These two examples show that supramolecular substrate orientation by hydrogen bonding is a powerful approach to control the regioselectivity in challenging C–H borylation reactions.

**Scheme 51:**
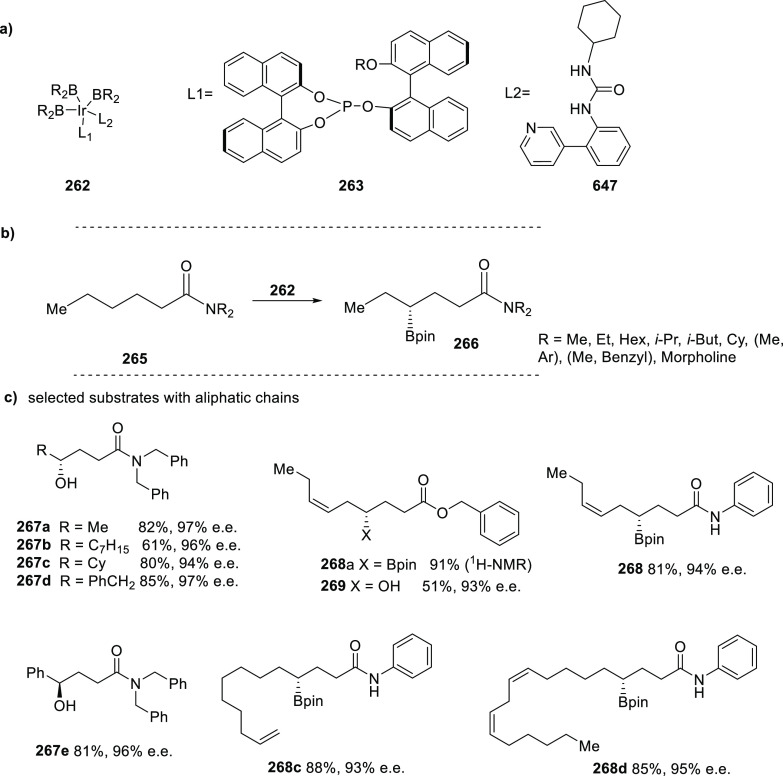
(a) Iridium Catalysts (**262**) with a Chiral Ligand (**263**) and a Ligand for Substrate Orientation (**264**); (b) Leading to Remote C–H Borylation Producing the Product **266** in High Enantioselectivity (up to 99% e.e.); (c) Part of the Substrate Scope with Aliphatic Chains for Asymmetric Remote C–H Borylation For substrates **267** the borylated intermediate product was directly converted to the alcohol.

The strategy of substrate orientation by hydrogen bonding was further extended by the group of Sawamura to asymmetric C–H borylation of aliphatic amides and esters.^406^ To this end, they used chiral iridium complexes (**262**) based on a chiral Bisnaphtol-based bulky phosphite ligand (**263**) and a phenyl urea functionalized pyridyl ligand (**264**) as shown in Scheme 51a. Chiral information is provided by the phosphorus ligand (**263**), whereas the urea moiety on the pyridyl ligand (**264**) preorganizes the substrate with respect to the iridium center. Secondary amides and esters were converted in high e.e.’s with the borylation occurring at the *gamma* position of the substrate. Variation of the pyridyl ligand (**247**) in which the nitrogen is placed in the *para* or *ortho* position with respect to the phenyl urea group also resulted in borylation at the *gamma* position but with lower selectivity and yield. When the reaction was carried out at elevated temperature, the enantioselectivity dropped only slightly (from 99 to 87% at 80 °C). The substrate scope was very broad, as a variety of substrates with different functionalities at the amide were converted. Also, the reaction was shown to be tolerant to variation at the aliphatic tail, and larger substrates and substrates with alkenes in the chain were still selectively converted (Scheme 51c).

### 4.4 Radical-Type Carbene and Nitrene Transfer Reactions

**Figure 18:**
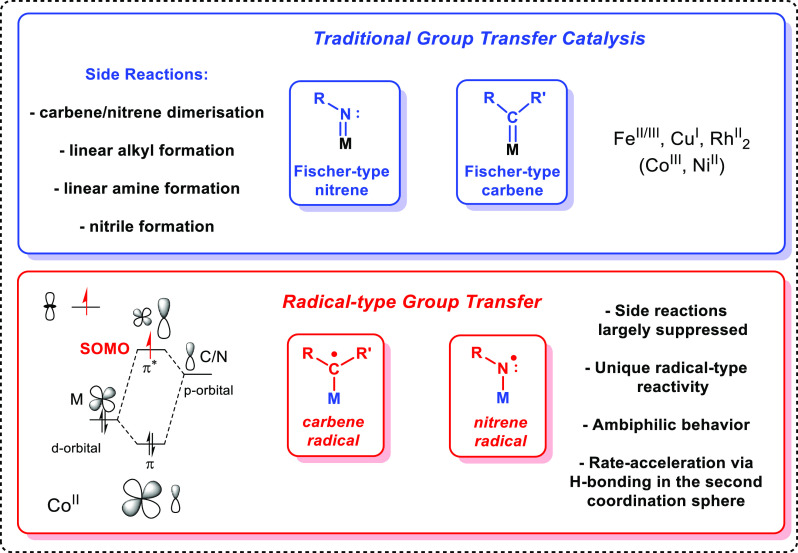
Formation of radical-type carbenes and nitrenes in cobalt(II) catalyzed group transfer catalysis.

Metallocarbene and nitrene radicals are important intermediates in a variety of radical-type carbene and nitrene transfer reactions mediated by cobalt(II) catalysts. Implementing hydrogen bonding interactions between structural motifs in a catalyst with the substrate has been shown to be a powerful method to control both the activity and (enantio)selectivity of these reactions. In particular, chiral porphyrin complexes with HB donors attached to the ligand framework have been shown to be powerful catalysts for enantioselective radical-type reactions. Such reactions typically rely on a combination of the unique electronic structures of the key intermediates and supramolecular interactions with HB moieties in the second coordination sphere of the catalysts.^407−411^ Instead of traditional (Fischer-type) carbene and nitrene complexes of cobalt(II), unique cobalt(II)-carbene and nitrene radicals are formed by single electron transfer from cobalt to the carbene or nitrene moiety. Computational and EPR studies have shown that the spin densities of the carbene and nitrene complexes are almost entirely located on a *p*-orbital of the carbene or nitrene moiety, as is illustrated in Figure 18.^412^ The resulting substrate radical species are key intermediates in a broad range of catalytic group transfer reactions to C–H, C=C, and C≡C bonds. A unique feature of these systems is the fact that intramolecular electron transfer from cobalt to the substrate occurs simultaneously with generation of the carbene or nitrene moiety. This leads to one-electron reduced Fischer-type carbenes or nitrenes, resulting in lower electrophilicity of activated substrates, rendering them partially nucleophilic and imposing radical reactivity together with a unique selectivity (Figure 18).

**Figure 19:**
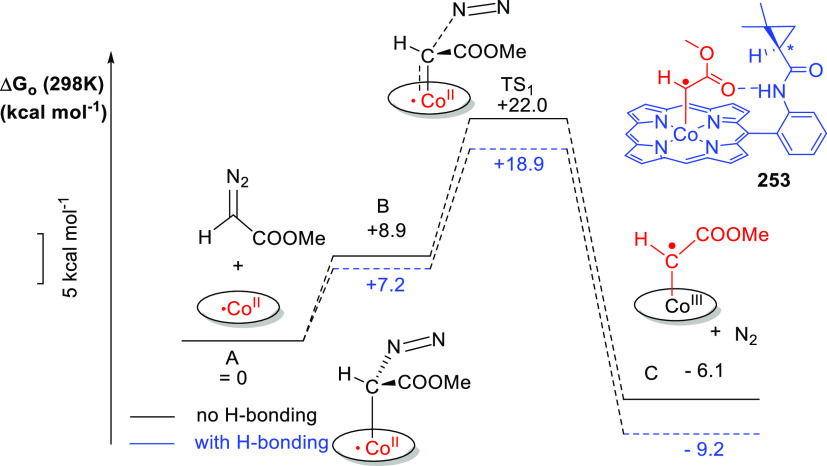
Hydrogen bonding leading to transition state stabilization in cobalt(II) catalyzed group transfer catalysis.

Increased electron density at bound substrates also provides novel opportunities to control the enantioselectivity of these radical-type reactions using hydrogen bonding interactions in the second coordination sphere. The high activity and enantioselectivity of such reactions can be explained by combined transition state stabilization and substrate orientation. This is illustrated in Figure 19 for carbene radical formation.^407^ In the presence of chiral amide HB donors in the second coordination sphere, formation of the carbene radical intermediate shows a lower barrier than without these HBs. Electron transfer from cobalt to the carbene moiety results in a stronger HB to the hypovalent carbene moiety than in the precursor, which explains the lower transition state barrier. At the same time, these interactions bring the chiral information of the catalyst close to the reactive substrate, which can lead to high enantioselectivities of the follow-up carbene transfer reactions. Similar effects play a role in radical-type nitrene transfer reactions with cobalt catalysts.^410,411^

**Scheme 52:**
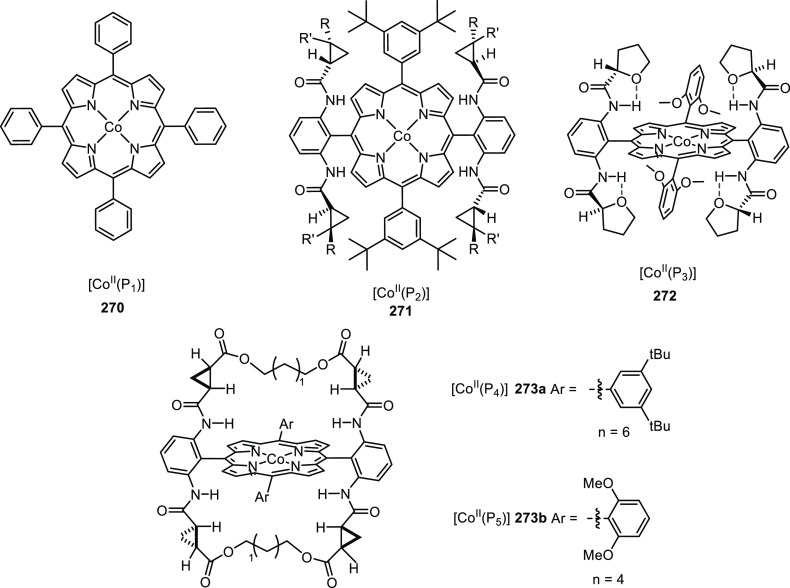
Selection of Cobalt(II) Porphyrins Used in Radical-Type Group Transfer Catalysis

Shown in Scheme 52 is a selection of cobalt(II) porphyrins that have been studied in radical-type group transfer catalysis. The nonchiral [Co^II^(P_1_)] (**270**) (P_1_ = *meso*-tetraphenylporphyrin) has been used extensively as a workhorse for the synthesis of several cyclic and noncyclic products via metalloradical catalyzed carbene transfer. The cobalt–carbene radical intermediates involved in these reactions undergo stepwise controlled radical addition or hydrogen atom abstraction (HAA) and then transform to various interesting structures in a mild and efficient manner. The redox-active carbene substrates thereby give access to the [Co^II^(P_1_)] catalyzed formation of, e.g., cyclopropanes,^413−418^ 2*H*-chromenes,^419,420^ furans,^421^ indenes,^422^ ketenes,^423,424^ butadienes,^425^ dihydronaphthalenes,^425^ piperidines,^426^ pyrrolidines,^427^ dibenzocyclooctenes,^428,429^ and monobenzocyclooctadienes.^429^ Carbene precursors for these reactions are typically diazo compounds (R_2_C=N_2_) and *N-*tosylhydrazones. With some exceptions,^417,430−432^ enantioselective carbene transfer reactions typically rely on catalysts capable of providing additional hydrogen bonding interactions in the second coordination sphere (Figure 19 and Scheme 52).

**Scheme 53:**
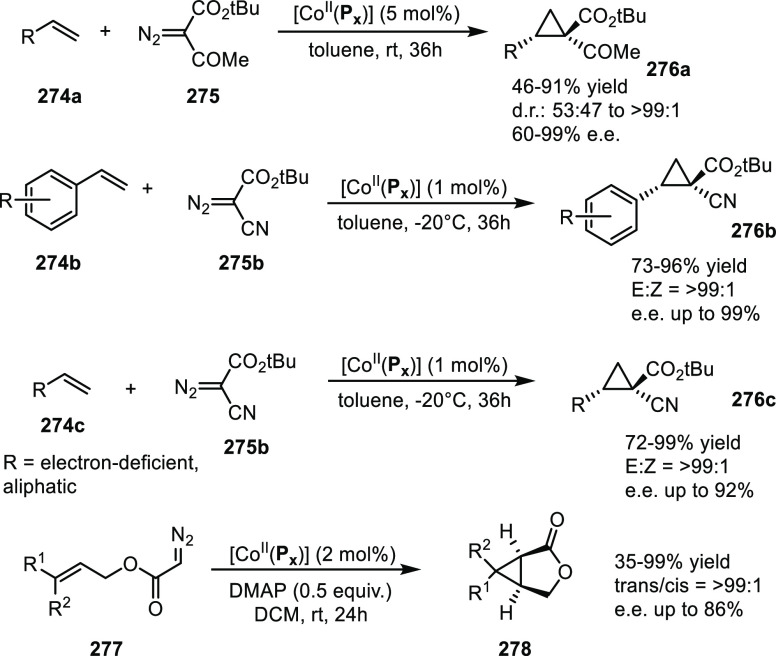
Asymmetric Cyclopropanation of Alkenes Catalyzed by Catalysts of the Types [Co^II^(P_2_)] (**271**) and [Co^II^(P_3_)] (**272**) and Analogs (See Also Scheme 52)

Scheme 53 shows examples of asymmetric cyclopropanation of aromatic, aliphatic, electron-rich, and electron-deficient olefins under mild reaction conditions.^415,418,433−444^ These cyclopropanation reactions are dominated by reactions involving mostly styrenes and alkenes bearing electron withdrawing and radical-stabilizing substituents. With some exceptions,^434,438,445−447^ most of these reactions involve diazo compounds (or their tosylhydrazone precursors) containing a single substituent at the carbenoid carbon atom. Catalyst [Co^II^(P_2_)] (**271**) and closely related analogs were also successfully applied in the asymmetric cyclopropanation of allyl α-diazoacetates and α-formyldiazoacetates, which were obtained in high yields and with good e.e.’s.^447−449^ Complex [Co^II^(P_3_)] (**272**) provides a more rigid structure due to intramolecular O···H–N hydrogen bonding interactions in the ligand backbone (indicated in red), which enables *trans*-cyclopropanation of styrene with HC(N_2_)(*p*-toluenesulfonyl) with excellent enantioselectivity.^415^

**Scheme 54:**
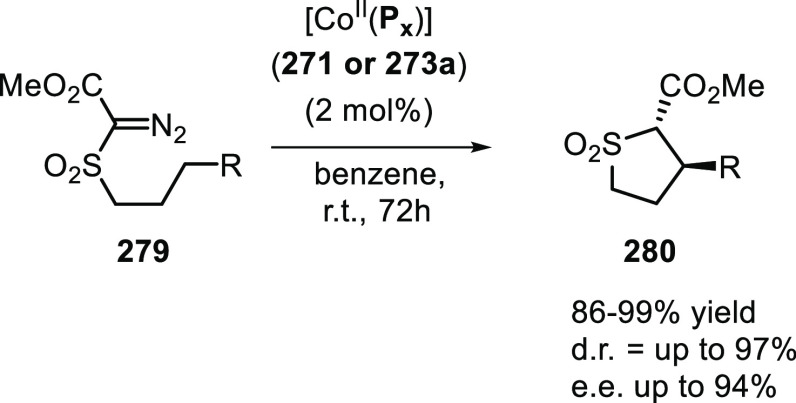
[Co^II^(P_*x*_)] Catalyzed (See Also Scheme 52) Asymmetric C–H Alkylation Leading to Cyclization of α-Methoxycarbonyl-α-Diazosulfones R = aryl, triazole, alkenes, and allene.

Next to cyclopropanation, several (enantioselective) cyclization reactions proceeding via carbene insertion into (activated) C–H bonds have been disclosed. Hydrogen bonding interactions between the substrate and the catalyst again play a crucial role in some of these reactions, both to facilitate activation of the carbene precursor and, in particular, to control the enantioselectivity. For example, as depicted in Scheme 54, the chiral amidoporphyrin analogs of [Co^II^(P_2_)] (i.e., [Co^II^(P_4_)]) are also capable of asymmetric intramolecular 1,5-C–H alkylation/cyclization of α-methoxycarbonyl-α-diazosulfones to form sulfolanes in high yields and with good diastereo- and enantioselectivities.^450^

**Scheme 55:**
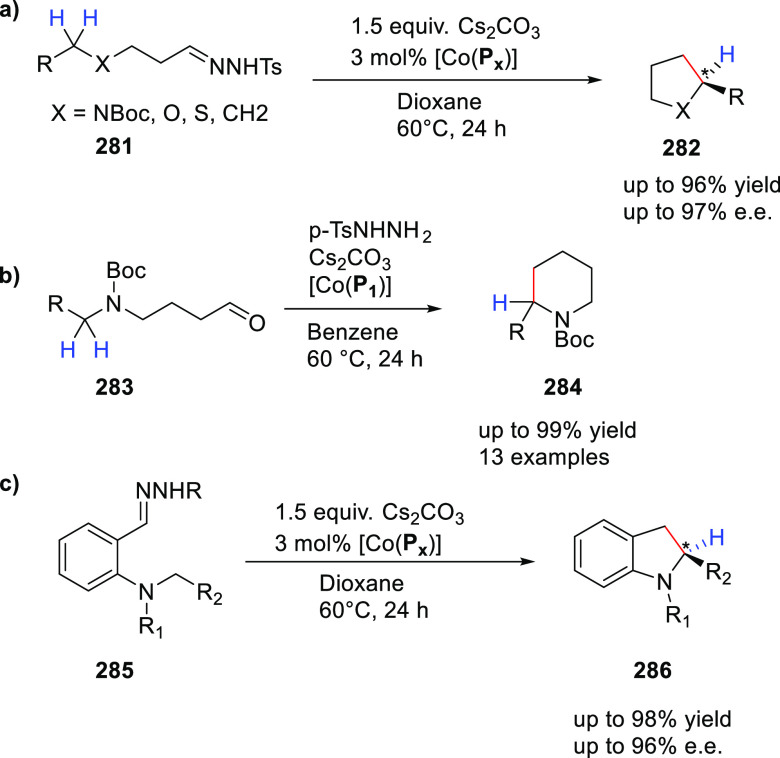
[Co^II^(P_*x*_)] Catalyzed (Asymmetric) C–H Alkylation Cyclization Reactions for the Synthesis of (Chiral) Pyrrolidines (a), Piperidines (b), and Indolines (c) Catalysts [Co(P_*x*_)] are of the type [Co(P_1_)] (**270**), [Co(P_2_)] (**271**), [Co(P_3_)] (**272**), and analogs.

Related protocols have also been developed for the synthesis of pyrrolidines (Scheme 55a, top),^447^ piperidines (Scheme 55b),^426^ and indolines (Scheme 55c).^451,452^

**Scheme 56:**
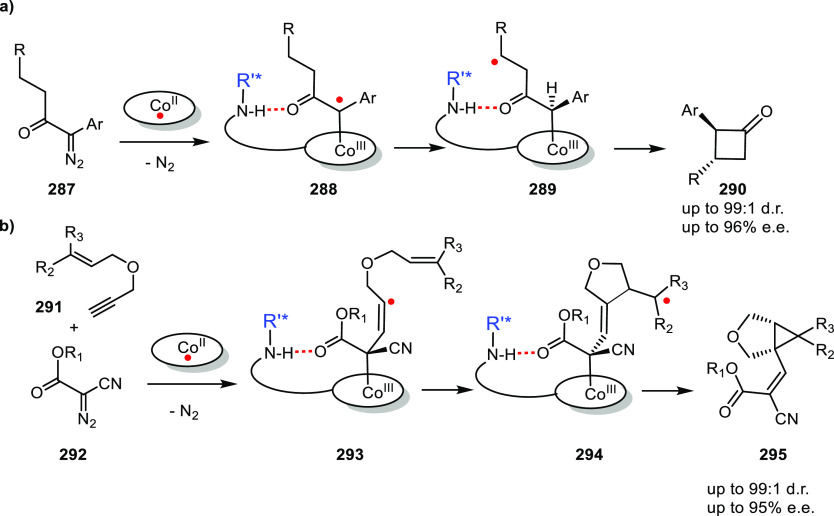
(a) [Co^II^(P_*x*_)] Catalyzed Asymmetric Cyclization Reactions Leading to Chiral Cyclobutanones (b) and Bicyclic Compounds via Cascade Radical Catalysis Catalysts [Co^II^(P_*x*_)] are of the type [Co^II^(P_2_)] (**271**), [Co^II^(P_4_)] (**273a**), [Co^II^(P_5_)] (**273b**), and analogs.

More recent examples from the Zhang group revealed that four-membered cyclic ketones also can be constructed in an enantioselective manner by a metalloradical catalyzed HAA and rebound sequence as shown in Scheme 56a.^453^ The related radical cascade reaction also allows for the enantioselective synthesis of bicyclic compounds illustrated in Scheme 56b.^454^ The enantioselectivities of these reactions again seem to be largely controlled by the hydrogen bonding interactions between the substrate(s) and chiral HB donors in the second coordination sphere of the catalyst.

**Scheme 57:**
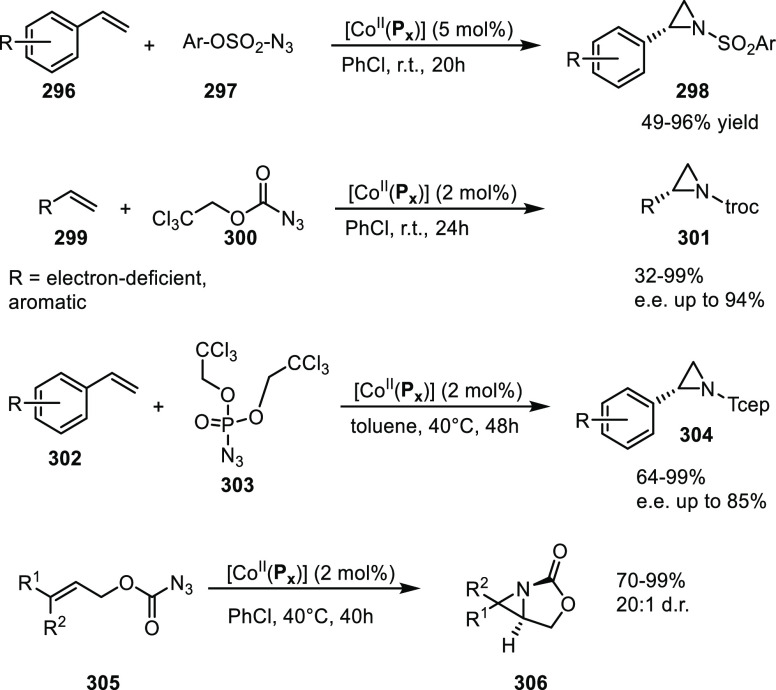
Selected Examples of [Co^II^(P_*x*_)] Catalyzed HB-Assisted (Asymmetric) Aziridination Reactions Catalysts [Co^II^(P_*x*_)] are of the type [Co^II^(P_2_)] (**271**), [Co^II^(P_3_)] (**272**), [Co^II^(P_4_)] (**273a**), [Co^II^(P_5_)] (**273b**), and analogs.

Just like radical-type carbene transfer reactions, radical-type nitrene transfer reactions can also be effectively controlled by hydrogen bonding interactions in the periphery of the catalyst binding site. As such, several (enantioselective) cobalt catalyzed aziridination reactions have been reported, as exemplified by those shown in Scheme 57. With few exceptions,^455−457^ the amide moiety of the macrocyclic ligand in the catalyst acts as the HB donor, while the substrate is a HB acceptor in the form of a (nitrene radical generated from) reactive, preactivated organic azide such as RSO_2_N_3_, (RO)_2_P(=O)N_3_, or ROC(=O)CN_3_, as shown in Scheme 57.^411,443,458−468^

**Scheme 58:**
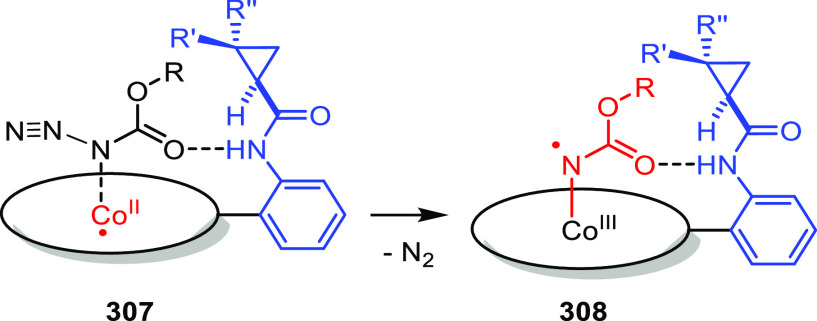
Hydrogen Bonding Interaction between the Chiral Amide Moiety of the Ligand, the Preactivated Azide, and the Reactive Nitrene-Radical Substrate

The nitrene radical species illustrated in Scheme 58 were unambiguously detected and characterized with ESI-MS spectrometry and EPR, UV/vis, IR, and XAS spectroscopy (supported by DFT), and IR and VCD studies clearly revealed hydrogen bonding interactions between the amide moieties of the catalyst and the substrate for these types of substrates.^469^ DFT studies showed that the barriers for activation of the azide substrate are lowered by these same hydrogen bonding interactions.^411^

**Scheme 59:**
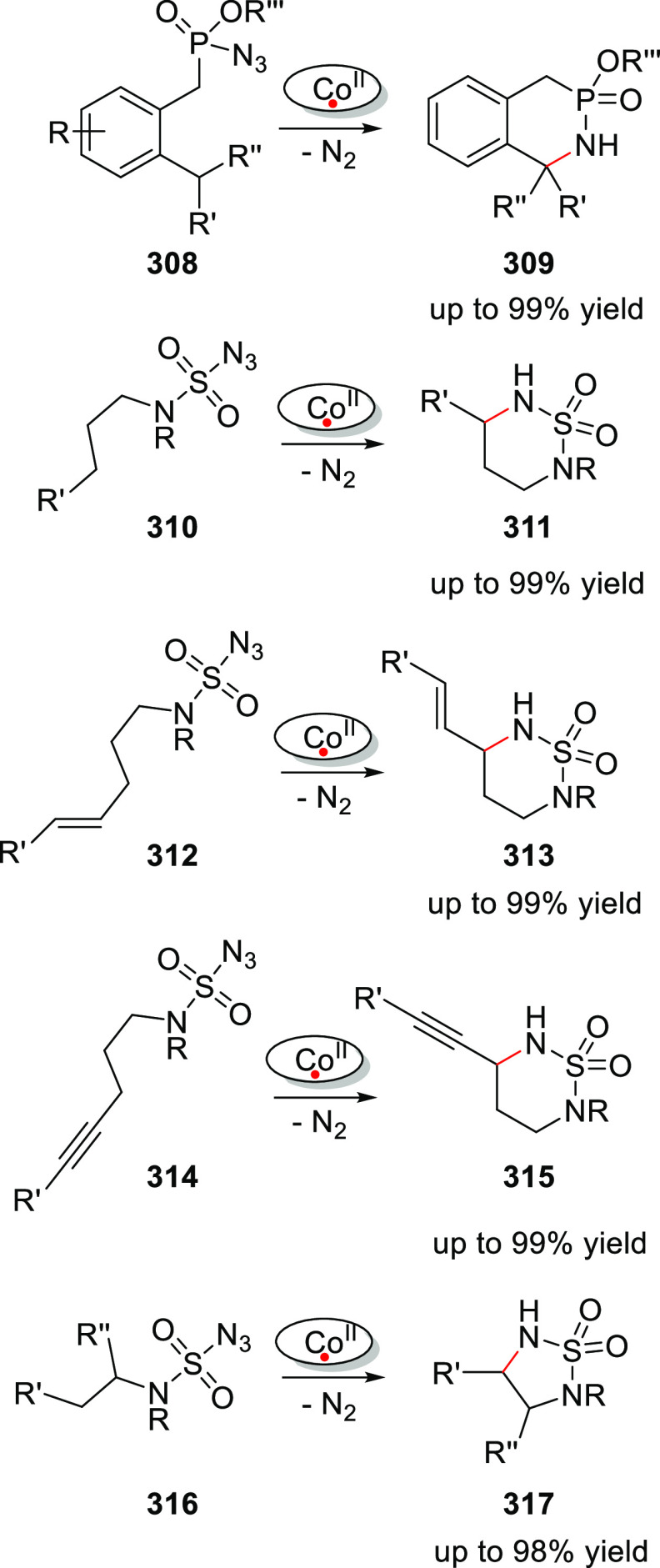
[Co^II^(P_*x*_)] Catalyzed Cyclization Reaction via Formal Nitrene Insertion into a C–H Bond of the Substrate Catalysts [Co^II^(P_*x*_)] are of the type [Co^II^(P_2_)] (**271**), [Co^II^(P_3_)] (**272**), [Co^II^(P_4_)] (**273a**), [Co^II^(P_5_)] (**273b**), and (nonchiral) analogs.

Several cyclization reactions to form five- and six-membered ring compounds proceeding via nitrene insertion into C–H bonds have also been developed (Scheme 59), with most reactions involving preactivated HB acceptor containing organic azides as the nitrene (radical) source.^465,470−473^

HB interactions between the catalyst and the substrate seem to play an essential role in most of these reactions, but conversions are mostly nonenantioselective. However, some of the more recently developed *D*_2_-symmetric catalysts bearing HB donors give surprisingly high e.e.’s,^474−476^ in particular for catalysts bearing tethered side groups of the types [Co^II^(P_4_)], [Co^II^(P_5_)], and their analogs (see Scheme 52).

**Scheme 60:**
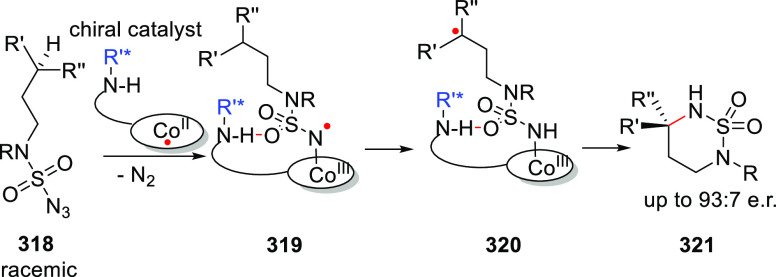
Enantioconvergent Cyclization of Racemic Alkylsulfamoyl Azides with Complexes of Type [Co^II^(P_2_)], Proceeding via Formal Nitrene Insertion into a C–H Bond of the Substrate

A recent report by the Zhang group is particularly noteworthy.^477^ In that study the authors have shown that *racemic* alkylsulfamoyl azide substrates can be converted with cobalt(II) catalysts of the type [Co^II^(P_2_)] in an enantioconvergent manner in order to produce chiral six-membered ring products. As is shown in Scheme 60, the reactions proceed via a HAA step converting the chiral center of the racemic substrate into a planar carbon radical, followed by an enantioselective radical-rebound step controlled by the chiral catalyst to produce the product.

**Scheme 61:**
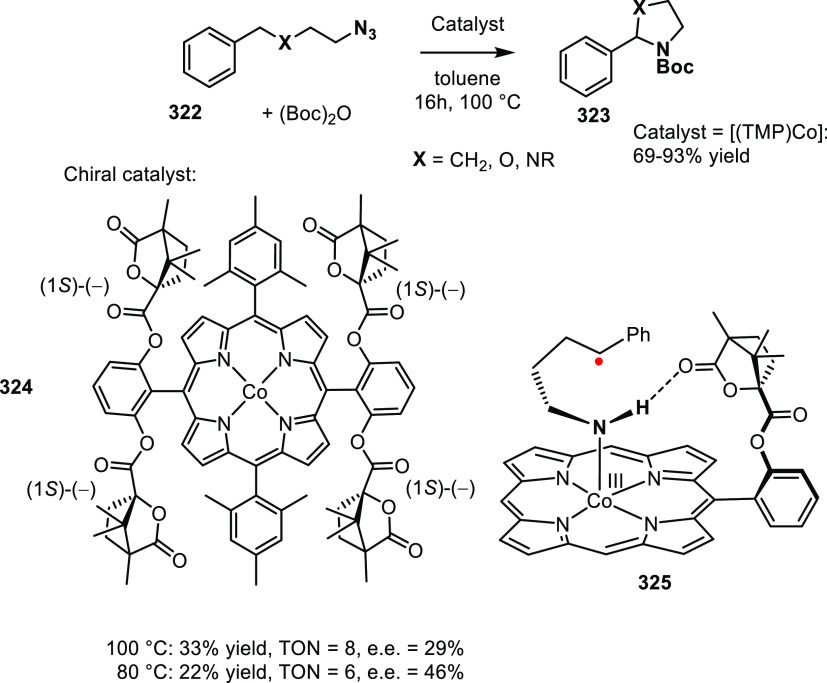
Synthesis of Pyrrolidines and Related Ring Compounds from Unprotected, Non-preactivated Aliphatic Azides

In addition to the examples given above where substrate orientation is achieved by a HB, this principle can also be combined with the effects of steric interactions and van der Waals forces in the second coordination sphere. For example, as illustrated in Scheme 61, enantioselective intramolecular C–H amination can be steered to produce pyrrolidines and related five- and six-membered ring compounds from unprotected and non-preactivated aliphatic azides.^478^

Some intermolecular nitrene insertion reactions into benzylic C–H bonds have also been disclosed,^479,480^ although thus far, only a few examples involve HB assistance from the ligand.^481^ To our best knowledge, only a single example of a HB-assisted enantioselective intermolecular nitrene C–H insertion reaction has been disclosed.^482^

### 4.5 Hydrogen Bonding in Oxidation Catalysis

**Figure 20:**
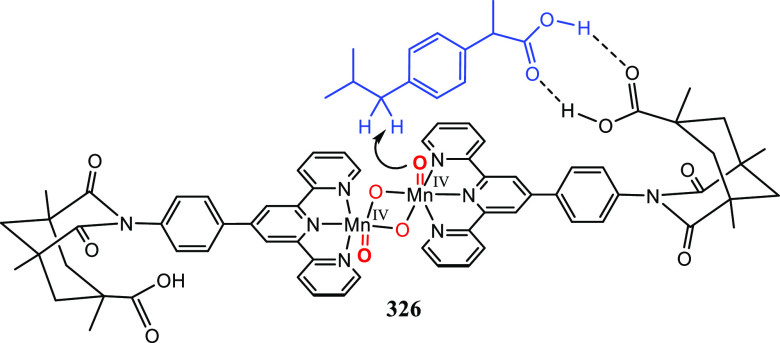
Crabtree’s dinuclear Mn oxidation catalyst containing a H-bond recognition site.

Hydrogen bonding interactions have also been shown to be useful in enabling site-specific reactivity in oxidation catalysis. An illustrative example is the supramolecular manganese catalyst developed by Crabtree and Brudvig (Figure 20), which is equipped with a carboxylic acid that forms hydrogen bonding interactions with the substrate to position the targeted benzylic position close to the reactive dinuclear Mn-oxo site.^483^ The terpyridine ligand of the Mn catalyst contains a rigid U-shaped motif with a carboxylic acid moiety acting as the H-bonding recognition site for substrates containing complementary carboxylic acids for H-bonding. The resulting supramolecular catalytic systems are capable of regioselective oxidation of the benzylic C–H bonds of ibuprofen with oxone (∼70% conversion, with 97% selectivity). The selectivity for the remote benzylic position is enforced by H-bonding interactions between the substrate and the catalyst, which was proven by comparison with the results with a similar complex without the substrate recognition site producing oxidized product at both benzylic positions in only a 3:1 ratio. Other control experiments using the ester variant of the substrate and performing the reaction in the presence of acetic acid also led to a loss in selectivity. The C–H bonds of the distant tertiary carbon atoms of *trans*-4-methylcyclohexyl acetic acid can also be selectively oxidized with this catalyst, but the conversions and yields are lower for this substrate. A mixture of *cis*- and *trans*-4-methylcyclohexyl acetic acid leads to selective conversion of the *trans* isomer, which has been proposed to be the result of steric blocking of the reactive site from access to the unbound substrate.^484^

**Figure 21:**
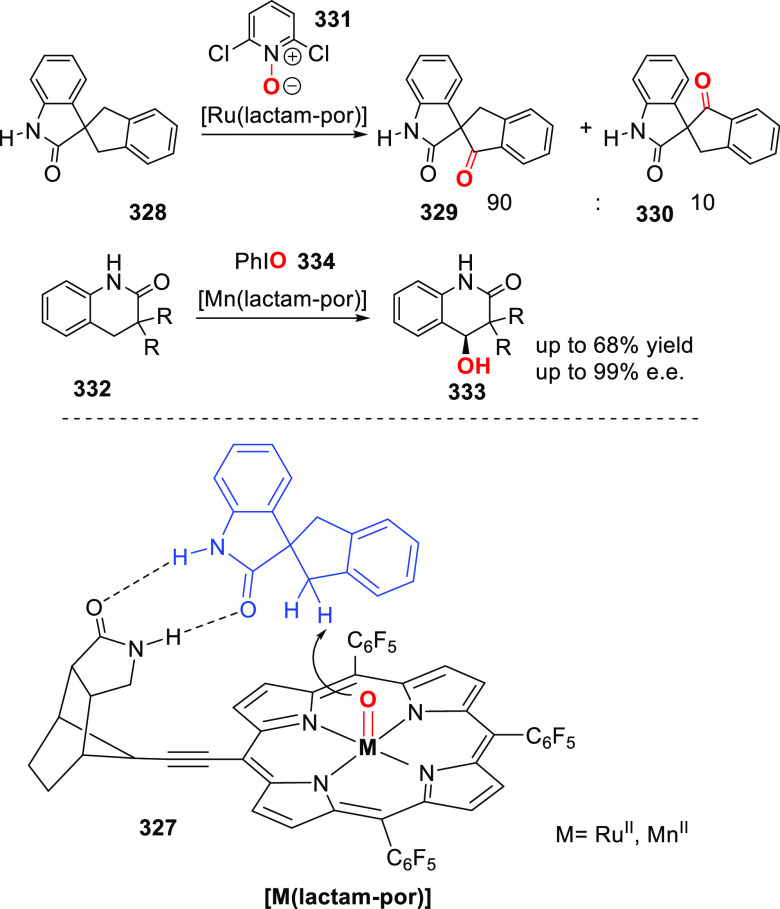
Stereoselective C–H oxidation mediated by directional substrate recognition in the second coordination sphere as reported by Bach and co-workers.

Similar approaches were explored by Bach and co-workers (Figure 21). They used a [Ru(porphyrin)] complex as the oxidation catalyst, equipped with a lactam binding motif as the DA HB array. A series of substrates containing a complementary DA HB array could be oxidized stereoselectively.^485^ As such a high enantiomeric ratio was achieved (95:5 e.r.; 20% yield), the yield could be improved to 70% by addition of an auxiliary oxidant. Interestingly, alkylation of the N–H bond of the substrate repressed the yields substantially, thus showing the importance of H-bonding recognition during catalysis.

**Figure 22:**
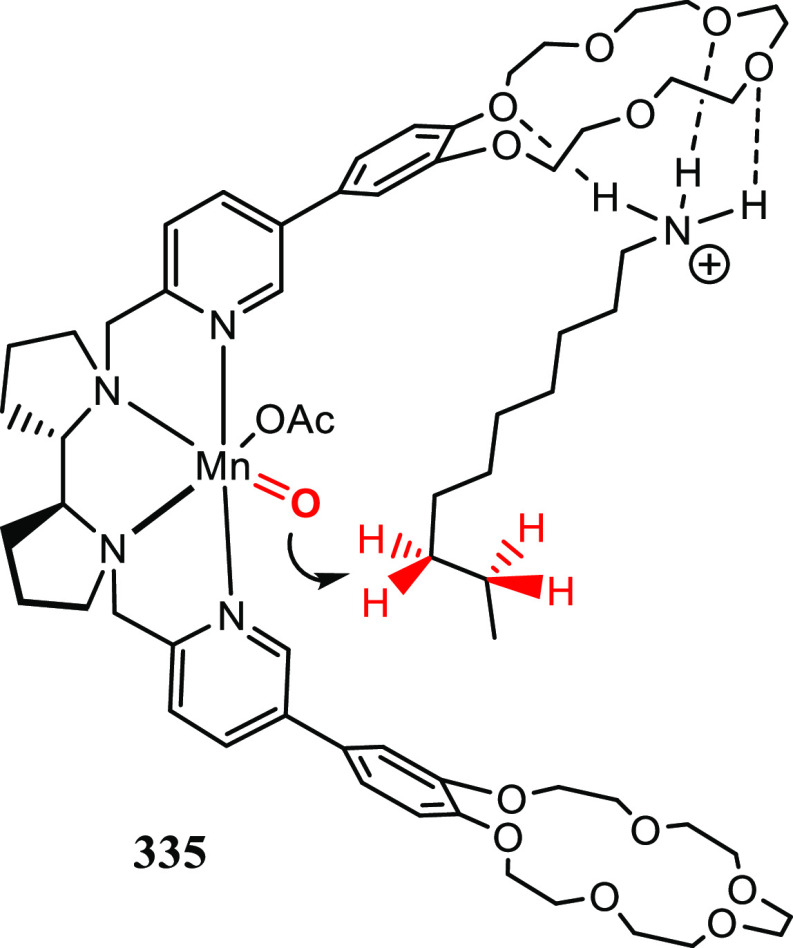
Site-selective oxidation of the C8 and C9 positions of linear ammonium salts directed by H-bonding interactions with a crown ether in the second coordination sphere of the Mn catalyst reported by Costas and co-workers.

Enantioselective oxidation of the benzylic C–H bonds of 3,4-dihydroquinolones was also successful.^486^ For this purpose Ru was replaced by Mn, resulting in a more efficient catalyst with a decreased tendency for overoxidation. High enantioselectivities (up to 99% e.e.) with preference for the (*S*)-alcohol were obtained for a broad range of substituents. Again, the importance of H-bonding was shown by alkylation of the N–H bond, leading to a close to racemic mixture. This work was extended to dirhodium complexes utilized with the same binding motif for substrate orientation for the enantioselective C–H amination reaction.^487,488^ In a related approach, the group of Costas designed a supramolecular (White-type) aminopyridine Mn catalyst for site-selective oxidation of ammonium salts, using H_2_O_2_ as the oxidant.^489^ The catalyst is equipped with an 18-benzocrown-6 ether in the second coordination sphere that interacts with the ammonium ion functionality of the substrate and, as such, positions the C(8)–H and C(9)–H bonds close to the Mn=O site for hydroxylation (Figure 22). A selectivity of 81% for site-selective C–H hydroxylation of the C8 and C9 position in a series of linear alkyl ammonium salts with different chain lengths (C6 to C14) could be achieved. Control experiments revealed the importance of H-bonding between the ammonium group and the crown ether moiety: Addition of blocking agents to the crown ether, such as Ba(II), or alkylation of the N–H bonds of the substrate leads to loss of selectivity for oxidation of the C8/C9 position.

The above examples clearly show that hydrogen bonding is a broad and effective tool to achieve selectivity in C–H bond oxidation reactions, and future studies are likely to reveal many more applications.

### 4.6 Photocatalysis

Photochemical reactions such as photoinduced electron transfer (PET)^490^ and, more recently, also triplet energy transfer (EnT)^491^ have gained increasing interest in organic synthesis in the past years. Enantioselective photocatalysis is especially intriguing, as photochemical reactions such as photocycloadditions may generate multiple stereocenters in one step. A major challenge in this respect is that photocatalysis involves highly reactive intermediates, which typically follow unimolecular relaxation pathways, leading to rapid deactivation. In the past, photocatalysts have been combined with a second chiral catalyst in order to control the stereochemistry of the reaction.^492^ The short lifetime of excited states limits enantioselective catalysis in biomolecular systems, since deactivation is often faster than diffusion, and thus, chirality transfer from the chiral auxiliary is not compatible. In order to achieve better enantioselectivity by chiral transfer reagents, novel concepts are required that are able to cope with this challenge. Meggers has demonstrated that chiral-at-metal photocatalysts can serve as bifunctional catalysts providing high enantioselectivity due to “enantioface separation”.^493,494^ As was detailed in the previous sections, hydrogen bonding serves as a general, highly potent tool to preorganize substrates and catalysts. In the following, recent advances will be discussed where enantioselective photocatalysis is achieved by hydrogen bonding strategies with a focus on transition metal-based catalysts.

Research on hydrogen bonding in photocatalysis has initially focused on organic dyes as photocatalysts. In the first proof-of-principle study, Krische and co-workers combined a hydrogen bonding motif for substrate binding with benzoquinone photosensitizer to catalyze intramolecular [2 + 2] photocycloaddition reactions of quinolones.^495^ Even though the enantioselectivity was relatively low in this system (22% e.e.), this study inspired further research in this direction. After this, hydrogen bonding as a strategy in photocatalysis has been mainly developed by the groups of Bach and Yoon. Several recent reviews on the topic have been published;^490,496,497^ therefore, we will only briefly mention selected examples of organic photocatalysts and focus our discussion on transition metal-based photocatalysts.

**Scheme 62:**
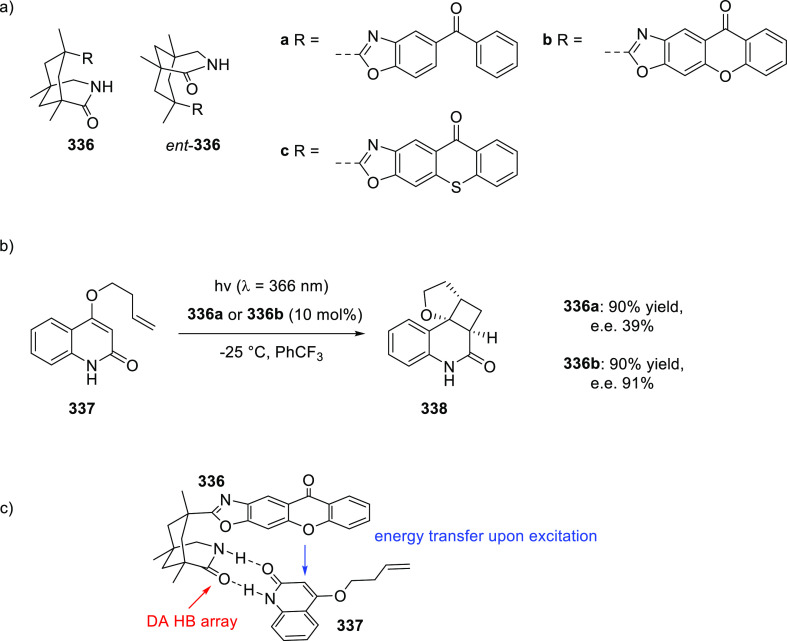
(a) Organic Dyes with a Hydrogen Bonding Site (**336a**–**c**) Developed in the Lab of Bach; (b) Intramolecular [2 + 2] Photocycloaddition of Quinoline (**337**) Proceeds with Higher Enantioselectivity with the More Rigid Xanthone (**336b**) Compared to Benzoquinone Dye (**336a**); (c) Substrates Bind to the Lactam Binding Motif by the DA HB Array

Bach developed a lactam-binding motif attached via a 1,5,7-trimethyl-3-azabicyclo[3.3.1]nonan-2-one backbone to a series of organic dyes (**336a**–**c**) shown in Scheme 62a.^496^ Substrates containing lactam units bind in a complementary fashion to this site via a DA HB array (see also Figure 2) as illustrated in Scheme 62c. For intramolecular [2 + 2] photocycloaddition of quinolones such as **337**, benzoquinone-based photocatalyst **336a** showed lower enantioselectivity compared to xanthone-based catalyst **336b** because it is not planar and substrates do not bind sufficiently strongly.^498^ More rigid and planar xanthone (**336b**) and thioxanthone (**336c**)-based catalysts yield overall better enantioselectivities in such reactions. Both catalysts (**336b** and **336c**), however, feature low stability in solvents which are prone to hydrogen abstraction.^499^ Therefore, low reaction temperature and relatively nonpolar solvents, such as trifluorotoluene (PhCF_3_), are required. Under these optimized conditions, a variety of reactions are catalyzed by **336b** and **336c**, featuring high yields and excellent enantioselectivities (Scheme 62b), including intra- and intermolecular [2 + 2] photocycloadditions^500−502^ as well as deracemization reactions.^503^

In comparison to organic dyes, many transition metal-based photosensitizers feature superior chemical stability and longer excited state lifetimes. For instance, iridium(III)- and ruthenium(II)-based photosensitizers display high activity and stability at low catalyst loading and, therefore, are both highly potent photocatalysts for PET and photosensitizers for EnT due to their high energy and long-lived triplet states.^491^

**Scheme 63:**
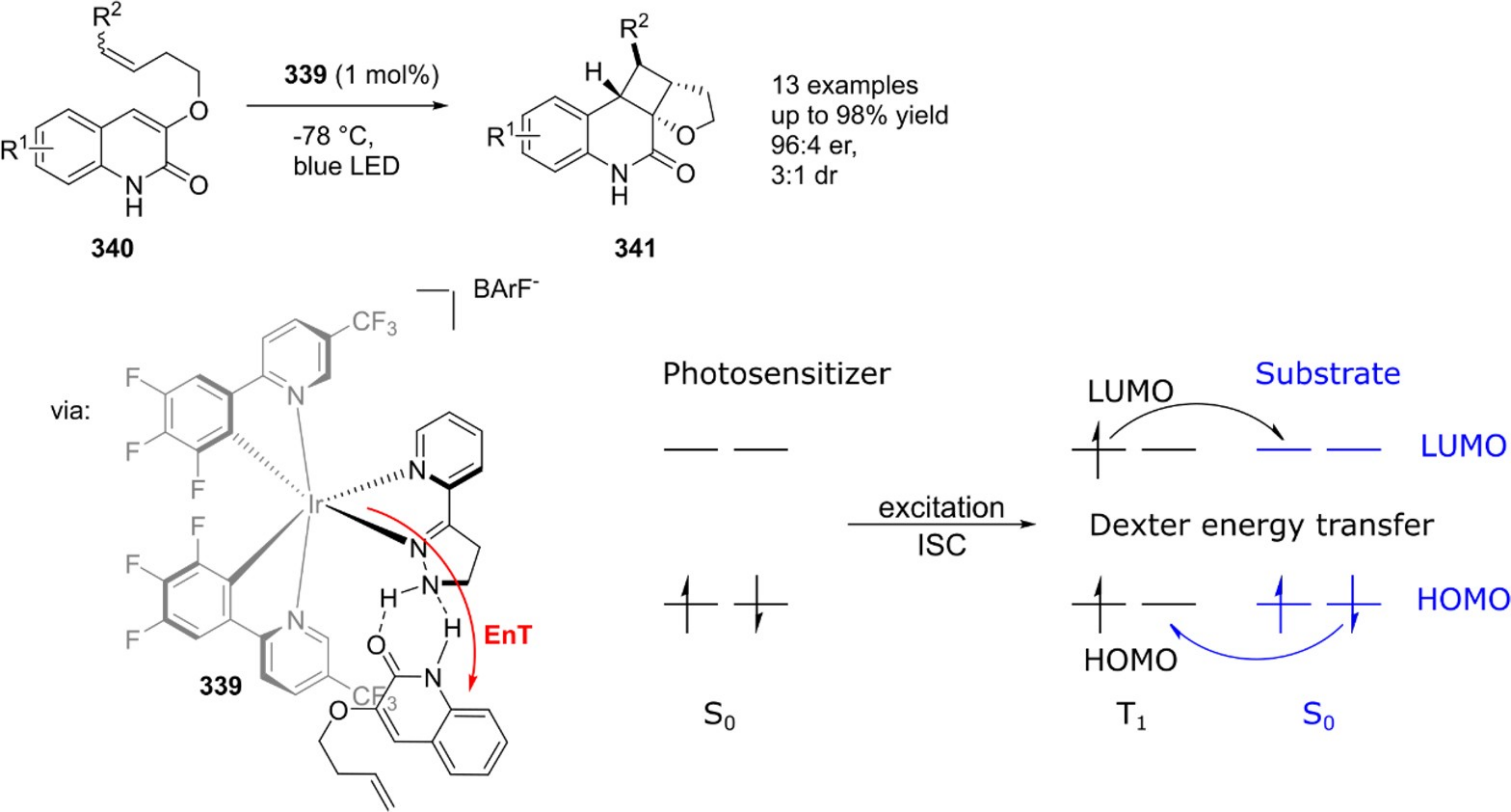
Intramolecular [2 + 2] Photocycloaddition Catalyzed by Iridium(III) Photosensitizer **339** with a Hydrogen Bonding Motif in the Ligand Energy transfer proceeds via the Dexter mechanism.

Yoon and co-workers developed the bifunctional iridium(III) polypyridyl complex (**339**) shown in Scheme 63, bearing a pyridylpyrazole hydrogen bonding moiety for hydrogen bonding in its ligand.^504^ This complex acts as a triplet photosensitizer in the asymmetric intramolecular [2 + 2] photocycloaddition of quinolones. As illustrated in Scheme 63, quinolone (**340**) binds via a DA HB array to the pyrazol ligand. Excitation of the iridium photosensitizer results in triplet energy transfer from the photosensitizer to the bound substrate following a Dexter energy transfer mechanism (e.g., simultaneous transfer of the excited electron from the sensitizer to the LUMO of the substrate and electron transfer from the HOMO of the substrate to the photosensitizer). The product (**341**) contains four new stereocenters, and due to the hydrogen bonding site, the reaction proceeds with high yield and more than 80% e.e. for 13 different substrates. The reaction scope is, however, limited to intramolecular reactions of substrates containing a lactam moiety.

**Scheme 64:**
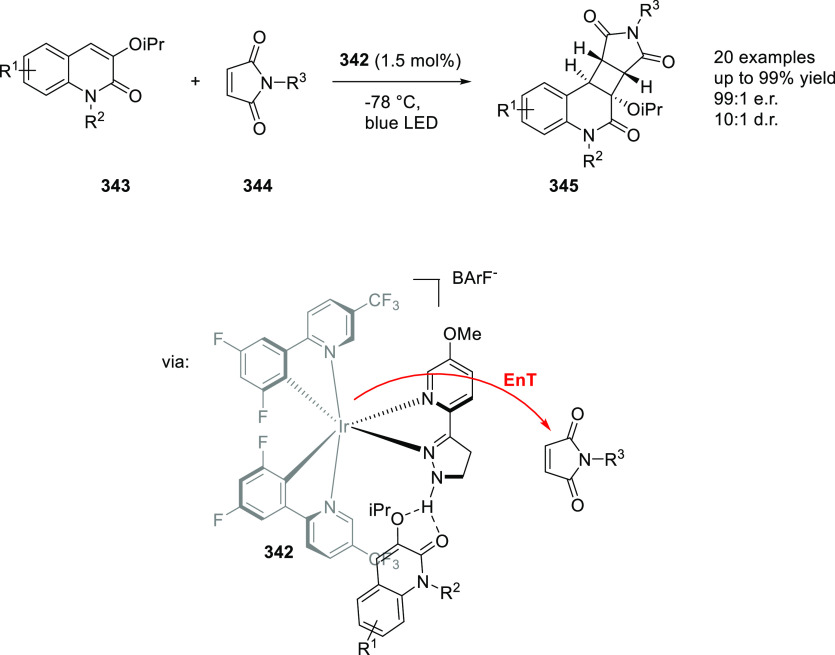
Intermolecular [2 + 2] Photocycloaddition with Modified Iridium(III) Photosensitizer **342** Energy transfer proceeds to the nonbound maleimide **344**.

Slight modification of the photosensitizer to iridium(III) complex **342** shown in Scheme 64 also enabled intermolecular photocycloaddition of 3-alkoxyquinolones **343** and maleimide **344**.^505^ In contrast to the previous example where energy transfer was directed from the sensitizer to the bound substrate, a combination of kinetic, spectroscopic, and computational studies showed that in this case, the reaction proceeds to nonbound maleimide **344**, yielding ^3^maleimide (a triplet). Substrate preorganization of hydrogen-bonded 3-alkoxyquinoline **343** enables rapid intermolecular cycloaddition to the activated maleimide, leading to excellent overall yield and enantioselectivity. This mechanism was supported by a variety of experiments. First, titration experiments monitored by NMR spectroscopy showed that the ground-state hydrogen bonding interaction of the catalyst is negligible with maleimide **344** compared to its interaction with quinolone **343**. Even though **344** does not bind to the photocatalyst, luminescence measurements suggested that it plays a critical role in the photocatalytic mechanism. Stern–Volmer quenching studies with photocatalyst and each of the reaction partners showed significantly larger quenching with maleimide **344** compared to quinolone **343**. Transient absorption spectroscopy further supported the assumption that the reaction proceeds via energy transfer to **344**: When iridium(III) complex **342** was excited in the presence of **343**, the lifetime of the excited state was not significantly altered. However, in the presence of **344**, the excited state lifetime was significantly reduced from 4.3 to 0.5 μs. Interestingly, variation of the structure of the photosensitizer showed that the ppy ligand has a larger effect on the reaction than the hydrogen bonding ligand. In addition, the identity of the 3-alkoxy substituent on substrate **343** has a large impact on the stereoselectivity.

**Scheme 65:**
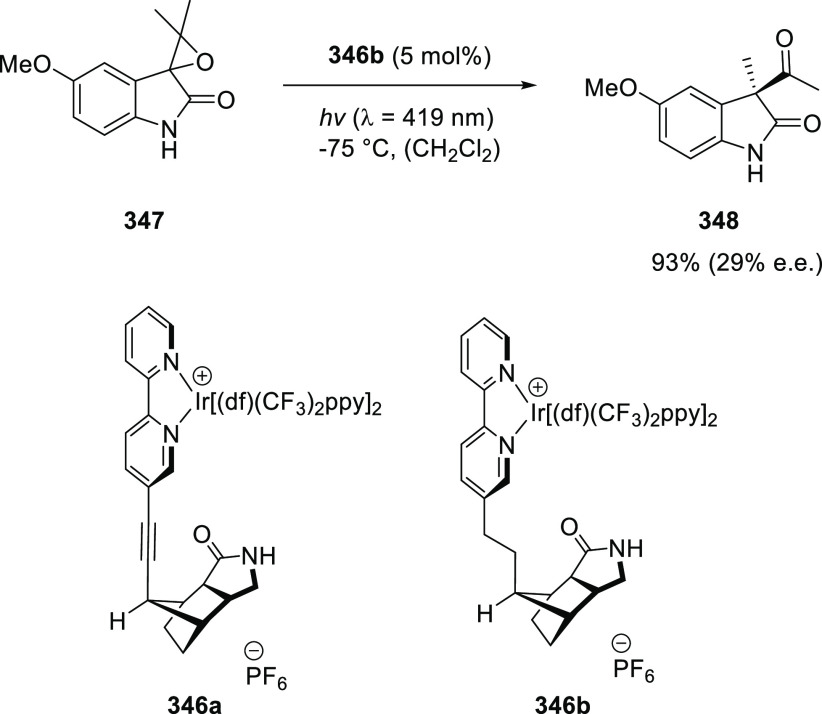
Epoxide rearrangement catalyzed by iridium(III) photosensitizer **346b** containing a lactam binding motif proceeds in high yields and moderate enantioselectivity.

Following up on their work on organic photosensitizers, Bach and co-workers developed transition metal-based chiral supramolecular catalysts where the metal center and chiral unit are covalently linked but spatially separated. In analogy to their organic photosensitizers containing the lactam unit for a DA HB array of interaction complementary substrates, they synthesized iridium(III) catalyst **346** shown in Scheme 65 with the same motif linked to the bipyridine ligand of the complex. As before, the lactam DA HB array enables hydrogen bonding with other lactams. The resulting complexes are kinetically labile, with lifetimes of 10–100 ns. Two different linkers between the photosensitizer and the hydrogen bonding motif were examined: alkynyl (**346a**) and enthano (**346b**). The investigation of the substrate and reaction scope was started with prochiral halide substrates, which were expected to form radicals upon reduction and C–X cleavage, that should then rapidly perform C–C coupling to form the cyclization product. For these substrates, instead of the desired cyclization product, only hydrodebromination products were observed. The authors suggested that the problem might lie in the reaction pathway of photoredox reactions: many photoredox mechanisms proceed via radical chain processes instead of via closed reaction cycles. Therefore, even substrates that are not kept close to the metal center (via hydrogen bonding) can be reduced eventually. The authors, therefore, turned their focus to reactions involving a triplet energy transfer (EnT) mechanism from the sensitizer to the substrate. Epoxide rearrangement of spirooxindole substrate **347** was studied. The ethano version of the iridium photocatalyst (**346b**) showed better conversion and overall yield (to both isomers) compared to the alkynyl-linked catalyst (**346a**). However, the enantioselectivity remained low (29% e.e. at best). The low enantioselectivity was explained by the fact that the enantioselectivity does not only depend on the steric bias of the respective catalyst but is also influenced by the rate of intermediate dissociation from the catalyst. In the case that the complex dissociation is faster than the selectivity determining step (epoxide rearrangement), the e.e. remains low. A similar observation has been made for the organic xanthone photocatalyst (**336b**) in previous studies.^506^

**Scheme 66:**
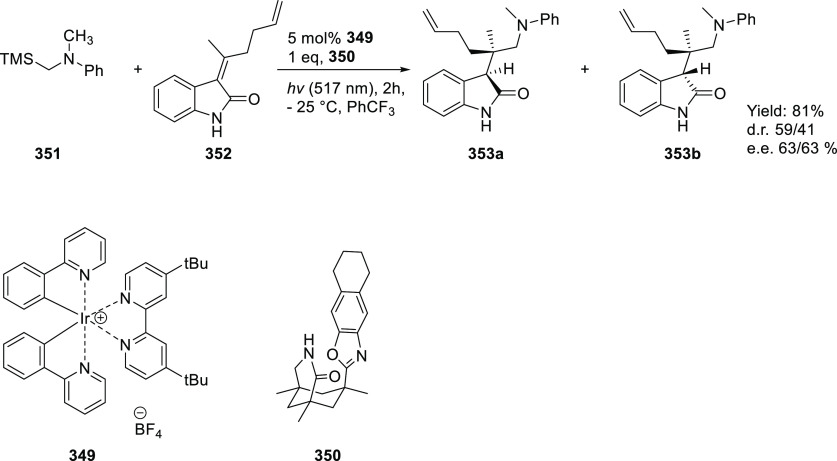
Dual System by Bach with Photocatalyst **349** and Chiral Template **350** for Radical Addition Reactions Here, the hydrogen bonding motif is not in the second coordination sphere of the metal complex but in the organic cocatalyst.

Turning back to bimolecular catalyst systems, Bach and co-workers merged an achiral photoredox catalyst (**349**) and chiral hydrogen bonding template (**350**) as shown in Scheme 66, which works very well as a chiral auxiliary in nonpolar solvent at low temperatures. It should be noted that in this system, the hydrogen bonding motif is not included in the second coordination sphere of the metal complex but as part of the organic cocatalyst. In analogy to previous studies, substrates containing a lactam binding motif bind to the template using the DA HB array provided by the lactam. In this study, radicals were generated via PET from the excited photosensitizer to trimethylsilyl (TMS) methyl-substituted amines (**351**). 3-Alkylidene indolin-2-ones such as **352** are readily bound to chiral template **350** and, thus, shielded from one side by the tetrahydro-1-oxa-3-azacyclopenta[*b*]naphthalene motif. This hydrogen-bonded complex is attacked by radicals that are formed via photoinduced electron transfer from photosensitizer **349** to nonbound substrate **351**. Overall moderate to good enantioselectivities were observed.

### 4.7 Allylic Substitution

**Scheme 67:**
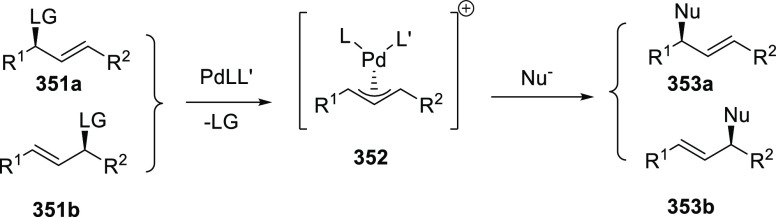
General Scheme for the Allylic Substitution Reaction

The palladium catalyzed allylic substitution reaction is a key reaction for organic synthesis and has been explored for decades, leading to many different protocols using a large variety of ligand scaffolds.^507,508^ In a typical reaction, illustrated in Scheme 67, the palladium allyl complex (**352**) is generated from a palladium(0) complex and an alkene susbstrate (**351**) with a proper leaving group. Subsequent outer sphere nucleophilic attack leads to the formation of the substituted product (**353**). The application of a chiral ligand in this reaction can result in the formation of the product with very high enansioselectivity, making it a versatile tool for organic synthesis. The outer sphere nucleophilic attack typically plays an important role in controlling the enantioselectivity. If unsymmetric 1,3-disubstituted substrates are used, that is when R1 and R2 are different (Scheme 67); the nucleophilic attack also determines the regioselectivity of the reaction. It has been demonstrated that hydrogen bonding between the functional groups of the ligand and the nucleophile can orient the nucleophile and with that a better control of the selectivity can be achieved.

**Scheme 68:**
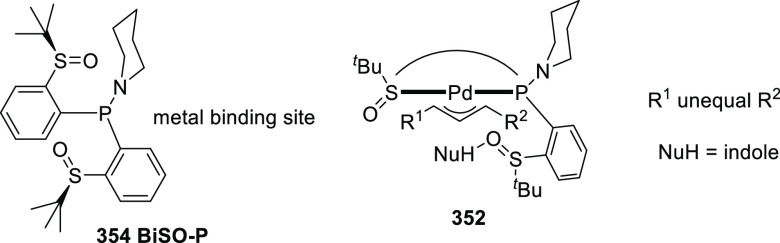
BiSO-P Ligand That Forms a Bidentate Chelated Palladium Complex, with a Dangling Sulfoxide That Can HB to the Indole Nucleophile

The bis(sulfoxide)phosphine ligand BiSO-P (**354**)^509^ shown in Scheme 68 forms palladium complexes in which the ligand coordinates in a P–S bidentate mode with a dangling sulfoxide. The complex was explored in the Pd catalyzed dynamic kinetic resolution of racemic unsymmetrically 1,3-disubstituted allylic acetates with indoles, providing a high level of stereocontrol. The high selectivity of this catalyst was explained by the presence the sulfoxide as a HB acceptor, directing the indole as a nucleophile by hydrogen bonding. NMR studies and Job plot analysis show that the indole indeed HBs with the complex, leading to supramolecular complexes with a 1:1 stochiometry.

**Figure 23:**
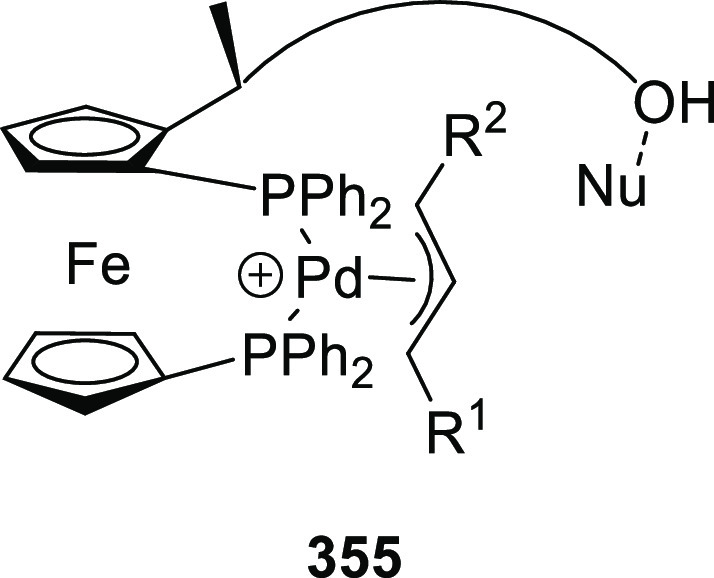
DPPF-based ligand with a dangling hydroxyl group proposed to direct the nucleophile by HB formation.

In an early paper by Hayashi et al., a diphosphine ferrocene-based ligand was reported with a dangling hydroxy group for nucleophile preorganization (**355**, Figure 23).^510^ This ligand provided a palladium complex that induced significant enantioselectivity (81% e.e.) in the allylation of 1,3-dicarbonyl compounds, which was proposed to be a result of hydrogen bonding between the OH of the ligand and the nucleophile. In a few other papers, hydrogen bonding between the ligand and the nucleophile has been proposed to play a role in controlling allylic substitution reactions.^511,512^

## 5 Summary and Outlook

Traditionally, the development of homogeneous catalysis involves the preparation of novel transition metal complexes that can be used for a certain chemical transformation. The activity and selectivity that such complexes possess are a result of the interplay between the ligand and metal properties in the complex. A large focus has, therefore, been on ligand development and descriptors for ligands to facilitate a more rational approach to catalyst development. High throughput experimentation and combinatorial techniques can speed up the search for catalysts for specific conversions, provided that libraries of sufficient size and diversity can be generated for the specific conversion at stake. More recently, additional strategies to control catalyst properties have been explored that involve the second coordination sphere, which is beyond the direct coordination sphere of the metal center. HBs appear to be very useful interactions in this context, as they typically have sufficient strength and directionality (section 2). In this review we have summarized the use of HBs to bridge two ligands that are coordinated to a metal center to effectively lead to supramolecular bidentate ligands (section 3), as well as the use of HBs to preorganize a substrate (section 4).

Supramolecular bidentate ligands have typical bidentate behavior, leading to larger control over the first coordination sphere. Concurrently, the ligand building blocks are generally easier to prepare. This is particularly the case for heterobidentate ligands (i.e., two different donor atoms), where the number of possible bidentates grows exponentially with the number of monodentate building block, thus enabling the generation of larger libraries of bidentates.

Utilizing HBs between ligand systems and the substrate has allowed for a more precise orientation of the substrate with respect to a metal center, which was shown to be of great benefit for the preparation of selective catalysts. In particular, HBs can be used to block (altered selectivity) or facilitate (higher activity) certain reaction pathways that a substrate could naturally undergo as determined by the electronic and/or steric biases of the substrate. Indeed, such reprogramming of selectivity and reactivity could be demonstrated in asymmetric hydrogenations (section 4.1), hydroformylations (section 4.2), C–H activations (section 4.3), radical-type reactions (section 4.4), oxidations (section 4.5), photochemical reactions (section 4.6), and allylic substitutions (section 4.7). The utilization of HBs in the second coordination sphere provide many examples of selectivities that are not reached with traditional catalysts. Moreover, the large variety of conditions that have been deployed, such as elevated temperatures and polar solvents, show that using HBs in this manner is a more powerful strategy than one may intuitively anticipate.

These advances notwithstanding, a more rational and systematic design approach of catalysts that make use of the full plethora of tricks to manipulate hydrogen bonding effects outlined in section 2.2 is an obvious next step. For example, very strong HB arrays with optimal secondary interactions (e.g., AAA/DDD or AAAA/DDDD) are relatively rare and have not been explored in the context of second coordination sphere control for catalysis. Moreover, the design of a catalyst from scratch addressing an unsolved selectivity issue in catalysis would truly demonstrate the power of using HBs in the second coordination sphere. Finally, the extension of the concepts reviewed here to include the deployment of other noncovalent interactions^71,345,513^ besides HBs (e.g., π–π stacking and halogen- or tetrel-bonding interactions) would broaden the scope of the approach. We are convinced that these types of approaches will become more standard in the design of the next generation of efficient catalysts with unique selectivities.
